# Microseismic monitoring with the quake neural operator

**DOI:** 10.1038/s41467-026-73965-6

**Published:** 2026-07-06

**Authors:** Hongyu Sun

**Affiliations:** 1 Department of Earth, Environmental and Resource Sciences, The University of Texas at El Paso, El Paso, TX USA; 2 School of Earth Sciences, The Ohio State University, Columbus, OH USA

**Keywords:** Seismology, Geophysics

## Abstract

Accurate monitoring of small-scale seismic events is essential for seismological studies. Traditional techniques rely on manual or automated seismic phase picking, which often leads to inaccuracies in microseismic monitoring due to unclear phase onsets. Here we introduce the Quake Neural Operator (QNO), a deep learning algorithm that builds earthquake catalogs directly from continuous data without explicit phase picking. As a multi-task operator, QNO utilizes classification and regression to detect and locate events across arbitrary seismic network geometries. We show that QNO successfully characterizes events where state-of-the-art phase picking fails. Applying QNO to the Geysers geothermal field, we identify nearly an order of magnitude more seismic detections than reported in routine catalogs. These results are validated through comparison with the Phase Neural Operator and visual inspection. QNO holds the potential to reveal undetected seismic activity, enhancing our understanding of subsurface processes critical to both natural phenomena and industry applications.

## Abstract (web summary)

This study introduces the Quake Neural Operator (QNO), a deep learning algorithm that builds earthquake catalogs directly from seismic data without phase picking. QNO increases the detection of small events lacking clear onsets, thereby enhancing our understanding of subsurface processes.

## Introduction

Microseismic monitoring offers valuable insights into subsurface processes, enhancing our understanding of fault mechanics, rock behavior, and fluid dynamics^1–3^. Beyond the study of natural earthquakes, the monitoring of seismicity induced by human activities has critical applications in geothermal energy development, mining, and carbon capture and storage^4–6^. While microearthquakes are typically too weak to be felt, their small vibrations are recorded in continuous seismic data streams, and are typically identified with a sequential workflow: seismic phases are picked from continuous data, picks are associated with seismic events, and event locations are determined using associated arrival times^7^. This process, however, often relies on manual or automated phase picking techniques, which are time-consuming and susceptible to human bias, particularly when handling large datasets with low signal-to-noise ratios (SNRs).

The advancement of deep learning has created opportunities to automate and enhance seismic monitoring^7–10^. Building on the sequential workflow of seismic monitoring, deep learning techniques have been used to replace steps in this procedure^11–19^. As an example of a deep learning-based phase picker, EQTransformer achieved more than a twofold increase in the number of detected events over the same time period, using only 18 stations, compared to a traditional catalog built from manually picked phases using 57 stations^13^. In addition to enhanced detection capability and efficiency, deep learning models also achieve arrival-time precision comparable to that of human analysts. Specifically, the mean absolute errors (MAEs) in P- and S-phase arrival times between human picks and the Phase Neural Operator (PhaseNO) are 0.05 s and 0.09 s, respectively^19^. With a sampling rate of 100 Hz, which is commonly used for regional seismic networks, an error of 0.05 s corresponds to only five samples, which is very small relative to the total waveform duration. Additionally, waveform denoising with neural networks before phase picking may enhance detection performance^20,21^. Zhou et al.^22^ perform event detection using full-waveform data before phase picking, with both tasks implemented using deep neural networks. Following phase picking, passive-source locations have been estimated from picked arrival times using machine-learning methods like random forests^23^ or graph neural networks^24,25^.

Recently, an increasing number of seismic monitoring and earthquake catalog construction workflows have been proposed^7,26,27^. Moving from single-task to multi-task learning, deep neural networks have demonstrated the potential to integrate multiple processing steps into a unified AI framework for seismic monitoring^28–30^. Other studies have developed customized seismic monitoring workflows by incorporating innovative AI techniques. For example, Tan et al.^31^ proposed an earthquake catalog construction workflow that detects events using waveform-based source imaging^32^ and AI-based semantic segmentation of 3D source images, followed by phase picking and source location. Alternatively, approaches that combine phase picking for detection with waveform-based source imaging for location have been proposed to automate the entire procedure, using either a classic short-term average/long-term average (STA/LTA) picker^33^ or deep-learning-based phase pickers^34–37^. Nonetheless, the overall detection capability and location accuracy may still be constrained by the initial phase-picking step.

Despite the advancements in seismic monitoring with deep learning, microearthquake monitoring still faces significant challenges due to the typically very low amplitudes of these events compared to the high levels of environmental noise. Many stations record waveforms that lack distinct phase onsets, complicating the process of phase picking. The inherently low SNR of microseismic events not only makes phase picking more difficult but also increases the potential for errors in earthquake location estimates. In many cases, template matching^38–40^ remains necessary to improve the detection of low-amplitude events or weak seismic signals after an initial earthquake catalog is built using either conventional^26^ or deep learning-based phase pickers^41,42^. However, template matching is known to have drawbacks, including being time-consuming and reliant on predefined templates^43^.

In addition to location approaches based on picked arrival times, seismic waveforms of detected events have been directly mapped to earthquake locations using deep learning techniques^8,44–48^. For instance, Zhang et al.^49^ applied a convolutional neural network to locate induced earthquakes in Oklahoma using a fixed seismic network. In contrast, flexibility with respect to station geometry can be achieved by incorporating station locations into the input of graph neural networks^50,51^, transformer-based models^52^, 3-D U-Net network^53,54^, or neural operators^55^. These methods often focus on relatively large earthquakes and may not perform well for microseismic events. Furthermore, these approaches typically assume that the waveforms of seismic events have already been extracted from continuous recordings. As such, they are not designed to operate directly on raw continuous data streams in which events have not yet been detected.

In this work, we introduce the Quake Neural Operator (QNO), a multi-task learning framework for end-to-end microseismic monitoring that directly detects and locates earthquakes from continuous waveform data recorded by seismic networks with arbitrary geometry. Moving beyond the traditional sequential workflow of phase picking, association, and source location, QNO jointly learns earthquake detection and source characterization within a unified framework. Because detection and location are intrinsically linked tasks, QNO exploits shared information between them to improve overall performance. We show that coherent spatio-temporal waveform patterns across multiple stations enable reliable earthquake detection and direct source location even when clear phase onsets are unavailable. By leveraging neural operator architectures (see Supplementary Note 1), QNO naturally accommodates irregular seismic network geometries and varying station configurations. Applications to microseismic monitoring demonstrate that QNO improves the detection of low-magnitude events and enables the construction of more comprehensive earthquake catalogs directly from continuous seismic data without explicit phase picking or phase association.

## Results

We begin by briefly summarizing the Quake Neural Operator (QNO), as its overall architecture constitutes a central methodological contribution underlying all subsequent experiments; full technical details are provided in the Methods section.

### Quake neural operator

The QNO is a multi-task operator learning model for automatic earthquake detection and location across multiple stations in a seismic network with arbitrary geometry (see Methods). It consists of a classification task and a regression task. The classification task ${{{\mathcal{Q}}}}_{{{\rm{class}}}}$ learns a probability function *p*(*x*, *y*, *z*) representing the likelihood of earthquake signals and noise in the spatial domain. For a given sample, this function is evaluated at discrete station locations (*x*_*i*_, *y*_*i*_, *z*_*i*_), yielding the probabilities ${{\bf{p}}}={\{{{{\bf{p}}}}_{i}\}}_{i=1}^{n}$, where *i* ∈ {1, 2, …, *n*} and *n* is the number of stations in the input. The regression task ${{{\mathcal{Q}}}}_{{{\rm{reg}}}}$ determines the earthquake location and origin time $(\widehat{x},\widehat{y},\widehat{z},\widehat{t})$. QNO operates flexibly with different seismic network configurations (see examples in Supplementary Figs. 1 and 2).

**Fig. 1:**
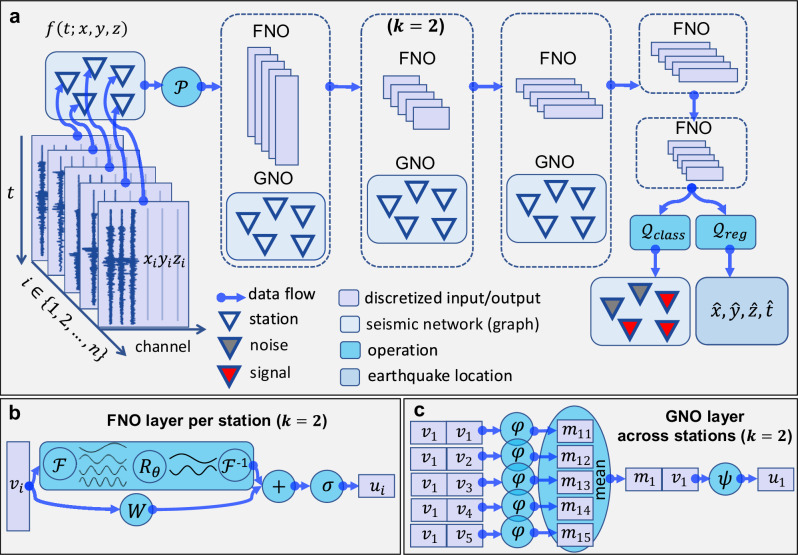
Quake neural operator (QNO). **a** Overview of the QNO architecture. An example with five stations (*n* = 5) is illustrated. **b** Operations in the second (*k* = 2) Fourier Neural Operator (FNO) layer. **c** Operations in the second Graph Neural Operator (GNO) layer. Only the feature updates at one station (*i* = 1) are shown. The architecture uses a multi-task learning framework consisting of a classification task ${{{\mathcal{Q}}}}_{{{\rm{class}}}}$ for earthquake detection and a regression task ${{{\mathcal{Q}}}}_{{{\rm{reg}}}}$ for earthquake location. The seismic wavefield *f*(*t*; *x*, *y*, *z*), recorded by multiple stations together with their arbitrary locations (*x*_*i*_, *y*_*i*_, *z*_*i*_), is encoded as the input. The outputs of ${{{\mathcal{Q}}}}_{{{\rm{class}}}}$ are earthquake and noise probabilities at each station, while the outputs of ${{{\mathcal{Q}}}}_{{{\rm{reg}}}}$ are the earthquake location and origin time $(\widehat{x},\widehat{y},\widehat{z},\widehat{t})$. ${{\mathcal{P}}}$: up-projection, ${{\mathcal{F}}}$: Fourier transform, ${{{\mathcal{F}}}}^{-1}$: inverse Fourier transform, *R*_*θ*_: learnable spectral kernel, *W*: point-wise linear transformation, *σ*: nonlinear activation function, *φ*: message generation function, *ψ*: node feature update function, *v*_*i*_: input feature at station *i* to a layer, *u*_*i*_: output feature at station *i* from a layer, *m*_*ij*_: edge feature between stations *i* and *j*, *m*_*i*_: message at station *i* obtained by mean aggregation. See Methods and Supplementary Note 1 for full technical descriptions.

Figure 1 depicts the architecture of the QNO model. The discretized input is represented as a three-dimensional tensor with axes for time, station, and channel. Each input tensor contains six channels: three corresponding to the three-component waveforms and three corresponding to the station locations (*x*_*i*_, *y*_*i*_, *z*_*i*_). The maximum duration for each input waveform segment is 15 s. The station axis dimension varies depending on the number of input stations sampling the spatial domain of the seismic wavefields, and the order of stations is arbitrary. The operation shared by both classification and regression tasks consists of multiple blocks of operator layers, where each block contains Fourier Neural Operator (FNO) layers^56^ and Graph Neural Operators (GNO)^57^ layers connected and repeated several times. FNO extracts temporal information locally at each station, while GNO enables communication among stations. Together, these components enhance robust spatiotemporal communication across the input seismic network. The resulting tensor from the shared component is flattened along the time and channel axes before being fed into separate tasks. Both classification and regression operators are down-projection layers composed of two fully connected layers. The output of ${{{\mathcal{Q}}}}_{{{\rm{class}}}}$ contains two channels, one for earthquakes and one for noise, for all stations. The regression output $(\widehat{x},\widehat{y},\widehat{z},\widehat{t})$ is obtained by applying mean pooling across the station dimension to the predicted location features generated at each station (see Methods).

### Benchmark with baselines

**Fig. 2:**
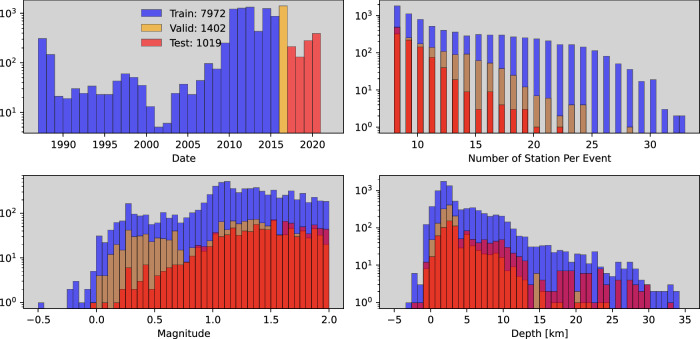
NCEDC seismic dataset for QNO training and evaluation. The dataset is split chronologically: earthquakes before 2016, in 2016, and from 2017 to 2021 form the training, validation, and test datasets, respectively. We select earthquakes with a magnitude of less than two and recorded by at least eight stations. Both training and test datasets cover a wide range of depth and magnitude.

We compare the performance of QNO with classic baselines for earthquake detection, location, and catalog building, respectively. Each baseline model was evaluated using the checkpoint that achieved the best validation performance during training. Both QNO and baseline models are trained with the Northern California Earthquake Data Center (NCEDC) dataset (Fig. 2). We select events with a maximum magnitude of two and split the dataset for training, validation, and test sets based on the year of occurrence, reflecting prospective deployment on future data (see Method). For completeness, random split results regardless of year are provided in Supplementary Figs. 4 and 5. As expected, performance under the random split exceeded that under the temporal split (Fig. 4), consistent with a smaller generalization gap when distribution shift is minimized.

**Fig. 3:**
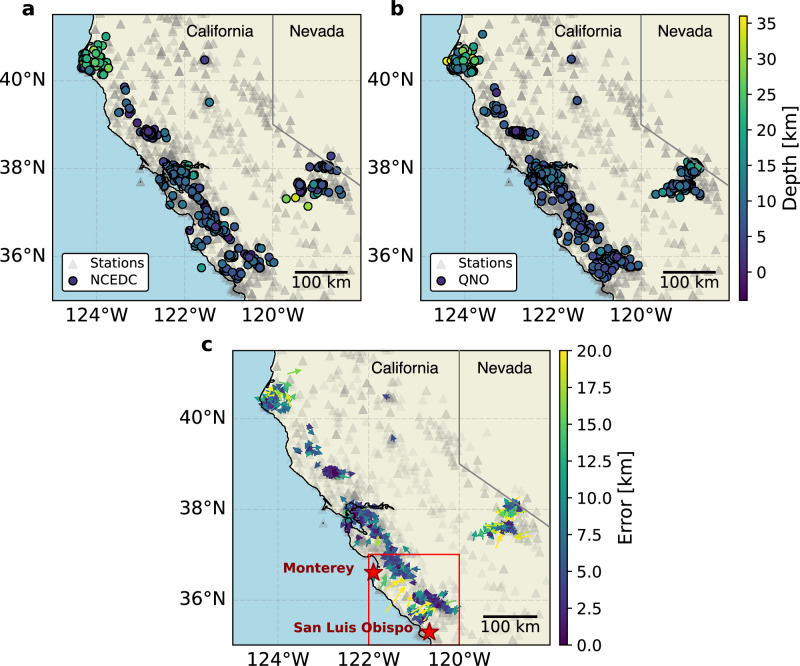
Comparison of earthquake locations between QNO and NCEDC catalog in the test dataset. **a** NCEDC catalog. **b** QNO prediction. **c** Arrows indicate the location errors for each event, pointing from the catalog epicenter to the corresponding epicenter predicted by QNO. A zoom-in view of the region bounded by San Luis Obispo and Monterey is provided in Supplementary Fig. 7.

**Fig. 4:**
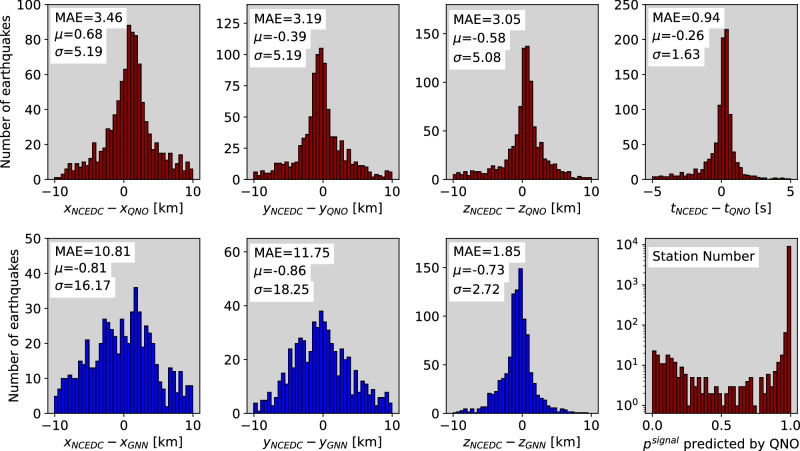
Performance comparison between QNO and SeismicGNN on the NCEDC test dataset. The red histograms represent QNO, and the blue ones represent SeismicGNN. The mean absolute error (MAE), mean (μ), and standard deviation (σ) of the histogram values are labeled in the corresponding histograms. QNO reports a signal probability of at least 0.8 on approximately 98.23% of 9416 recordings at each station of the total 1019 events.

For earthquake location, we compare QNO with SeismicGNN^50^. SeismicGNN incorporates spatial information in seismic source characterization by leveraging a graph neural network (GNN) to exchange information among stations. Node features at individual stations with SeismicGNN were extracted and processed with a convolutional neural network and multi-layer perceptron (MLP). As a result, QNO successfully determines the earthquake locations on all of the 1019 events in the test set across a wide range of areas in Northern California (Fig. 3 and Supplementary Fig. 6), although regions with higher seismicity rate and therefore more training samples exhibit better location accuracy (Supplementary Fig. 7). Compared with SeismicGNN, QNO provides more accurate geographical locations with lower mean absolute error (MAE), mean (*μ*), and standard deviation (*σ*) between predicted and catalog locations (Fig. 4). Although both methods share a similar distribution of depth prediction, the depth error predicted by QNO is slightly larger than SeismicGNN. In addition, QNO estimates the occurrence time of the input event relative to the starting time of the input waveforms, which enables us to build an earthquake catalog directly from continuous waveforms.

For the baseline for earthquake detection, we use PhaseNO^19^, a multi-station phase picking method built with neural operators. PhaseNO shares a slightly similar architecture with QNO, which uses both FNO and GNO together to extract features from multi-station seismic data, leading to better performance than single-station detection and picking models, such as PhaseNet^11^ and EQTransformer^13^. We first compare the performance of QNO and PhaseNO with decreasing signal-to-noise ratio (SNR) and show the advantage of QNO for detecting microseismicity in strong noise. Then we compare the performance of both methods on earthquake catalog building.

**Fig. 5:**
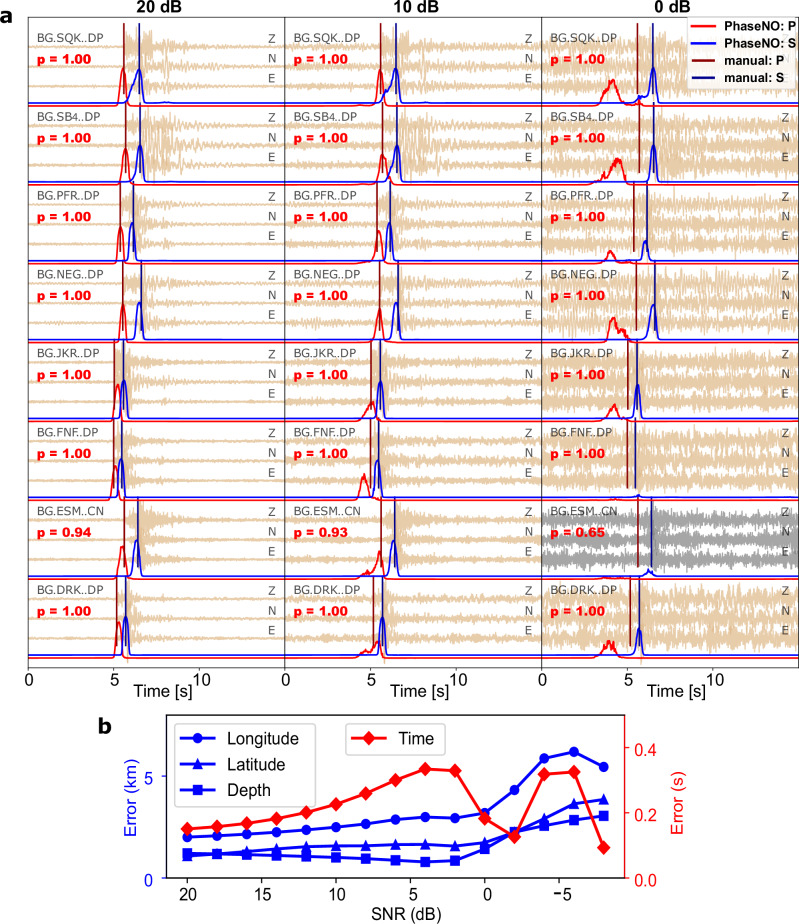
Example of detection and location performance under varying noise levels. We evaluate the impact of noise on detection and location performance by adding real noise waveforms to event nc73384250 while controlling the SNR from 20 dB to -8 dB. **a** Comparison of probabilities predicted by QNO and PhaseNO at SNR levels of 20, 10, and 0 dB. The red and blue curves show the pick probabilities predicted by PhaseNO. The two vertical lines mark the P- and S-wave arrival times from the NCEDC catalog, manually determined by seismologists. The probabilities (p) predicted by QNO for each input station are displayed in red beneath the corresponding station names. “Z'', “N'', and “E” indicate the vertical, north-south, and east-west components of the three-component seismic waveform at each station. **b** Location error of QNO across different noise levels. Additional examples are available in the Supplementary Figs. 8-17.

We compare the detection performance between QNO and PhaseNO using event nc73384250 from the test set as an example (Fig. 5). We introduce real noise waveforms to the raw waveform of this event, adjusting the SNR of the resulting waveform from 20 dB to -8 dB. At an SNR of 20 dB, QNO successfully detects all earthquake signals, outputting a probability close to one for all stations, while PhaseNO accurately identifies both P- and S-wave phases. However, as the SNR decreases, the performance of PhaseNO degrades, failing to detect P phases entirely at an SNR of zero. In contrast, QNO continues to detect the event at most stations even when the SNR reaches zero and consistently determines the event’s location. Meanwhile, the location errors of QNO increase quickly with decreasing SNR when SNR is below zero. This experiment demonstrates that, despite the inability to pick phases in strong noise, the waveform coherence among stations remains instructive, providing enough information to detect and locate the earthquake. QNO’s ability to detect events under high noise conditions enables us to monitor microseismicity where noise is consistently strong. More examples can be found in the Supplementary Figs. 8–17.

Interestingly, the predicted time and location errors do not always exhibit a strictly monotonic increase as the SNR decreases (Fig. 5). This non-monotonic behavior is likely due to the use of real noise recordings, which may contain microseisms, site effects, or instrument-related transients that introduce structured perturbations into the resulting waveforms. Since noise is added independently at each station and the QNO model integrates information nonlinearly across multiple stations, certain noise realizations may interfere less destructively with feature extraction, resulting in locally smaller errors. To further investigate this effect, we repeated the experiment using idealized Gaussian noise with the same SNR levels. The results in the Supplementary Fig. 17 show a more monotonic increase in errors as the SNR decreases, confirming that structured real noise can introduce irregular, non-monotonic effects on performance. This observation highlights QNO’s robustness to diverse noise structures and its ability to leverage spatiotemporal features to characterize seismic sources under complex real-world conditions.

### Generalization across seismic network configurations

In the dataset, each sample corresponds to a unique seismic network geometry, as each earthquake is recorded by a different subset of 8-32 stations drawn from the full seismic network (Fig. 3). These station configurations vary significantly across events, and we further enhance this variability during training by randomly adding noise-only stations with real noise waveforms from the STEAD dataset^58^. As a result, the model encounters a wide range of spatial sampling patterns during training, where each sample can be viewed as a different discretization of the spatial domain. In the ideal case of spatial data sampling that is sufficiently globally dense in the sense of Kovachki et al.^59^ Definition 4, QNO would be expected to generalize to unseen station layouts without requiring retraining. In our numerical results, the evaluation of the trained QNO performs well on unseen station layouts, as shown by its consistent performance across diverse station configurations during testing (Fig. 4).

In addition to spatial generalization, QNO is also capable of handling test inputs with different temporal resolutions. While the FNO layers require uniform sampling in time for each sample due to the use of the Fast Fourier Transform (FFT), the actual sampling rate of input waveforms can vary among samples. To demonstrate this, we upsample all waveforms in the test dataset from 1500 to 3000 data points (corresponding to increasing the sampling rate from 100 to 200 Hz) and directly evaluate the QNO model trained on the original 100 Hz inputs on the upsampled dataset. Supplementary Fig. 18 shows that the model maintains good performance under higher temporal resolution, highlighting its generalization capability across both spatial and temporal domains.

### Application to microseismic monitoring at the Geysers geothermal field

We apply the same trained QNO model evaluated in Figs. 4,5 to monitor microseismicity at the Geysers geothermal field and compare its performance in earthquake catalog building with that of PhaseNO + GaMMA^60^ using the same continuous seismic data. Our analysis begins with raw seismic data from HH, EP, and DP sensors (Supplementary Table 1), covering the period from June 8, 2024 (00:00:00) to June 18, 2024 (00:00:00) in a geographical region centered at (-122.78, 38.79). The monitoring range spans 1.2 degrees in both longitude and latitude. This monitoring period includes the *M*_*w*_ 4.5 mainshock on 2024-06-08 at 19:34:29, which was one of the largest earthquakes in the Geysers region in the past decade. We process the data using the same procedure as for the training dataset, bandpassing between 1 and 10 Hz and normalizing by dividing by the maximum value. Each input waveform is 15 s long, with a 5-s overlap between consecutive inputs, resulting in 360 predictions for every hour of data. With a sampling rate of 100 Hz, each input contains 1500 data points.

Several key hyperparameters are used to construct the final catalogs with either QNO or PhaseNO + GaMMA, ensuring low-quality results are filtered out. QNO predicts the signal and noise probabilities for all input stations. Based on these predictions, we first determine whether a recording is considered to contain an earthquake signal with a threshold ${T}_{{{\rm{QNO}}}}^{signal}\in [0,1]$ on the probability of an earthquake signal at an individual station. The other is the minimum number of stations on which an earthquake signal has been detected, denoted as ${N}_{{{\rm{QNO}}}}^{signal}$. An event is added to the catalog only when the number of stations with a signal probability greater than ${T}_{{{\rm{QNO}}}}^{signal}$ exceeds ${N}_{{{\rm{QNO}}}}^{signal}$. This approach allows for controlling the quality of the resulting catalog. Similarly, for PhaseNO, there is a threshold for determining phase picks for both P- and S-waves, denoted as ${T}_{\,{{\rm{PhaseNO}}}}^{pick}\in [0,1]$. These detected phases are then associated with events using GaMMA by requiring a minimum of ${N}_{\,{{\rm{PhaseNO}}}}^{pick}$ picks per event to ensure a high-quality association result. Note that an earthquake signal contains both P and S phases, although not all of them can be detected with PhaseNO. Ideally, PhaseNO detects seismic phases, whereas QNO detects the earthquake signal. Thus, the number of stations required to record an earthquake signal for its detection is typically smaller for PhaseNO compared to QNO when using ${N}_{{{\rm{QNO}}}}^{signal}={N}_{\,{{\rm{PhaseNO}}}}^{pick}$.

**Fig. 6:**
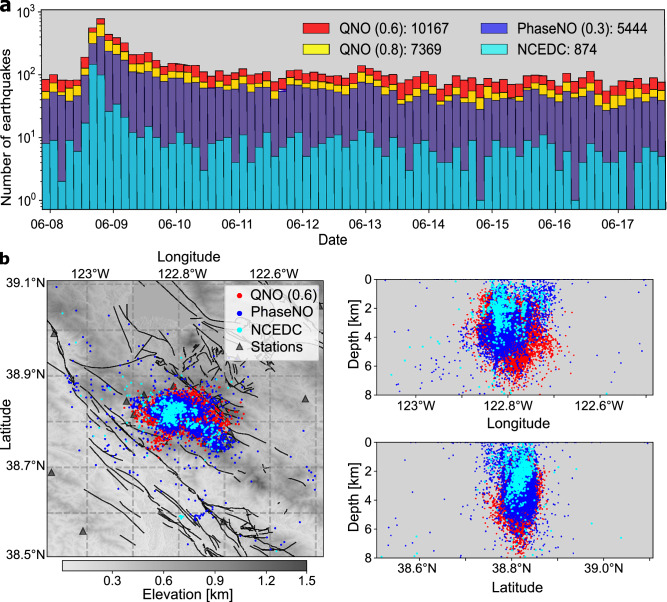
Comparison of detection performance between QNO and PhaseNO+GaMMA using ten days of continuous data from the Geysers geothermal field. **a** Number of earthquakes over time; **b** Earthquake locations. The thresholds for detecting a pick in PhaseNO and a signal in QNO at each station are indicated in parentheses in the histogram legends. Here, both QNO and PhaseNO+GaMMA require a minimum threshold of ${N}_{{{\rm{QNO}}}}^{signal}={N}_{\,{{\rm{PhaseNO}}}}^{pick}=16$ to identify a detection based on the number of detected signals (QNO) or picks (PhaseNO). The earthquake detection trends are consistent between QNO and PhaseNO+GaMMA, suggesting the effectiveness of QNO in earthquake detection. With a threshold of 0.6, QNO yields 10,167 detections, nearly one order of magnitude more than the NCEDC catalog for the same period. Although increasing the QNO threshold from 0.6 to 0.8 reduces the number of detections, QNO still consistently detects more events than PhaseNO, which uses a probability threshold of 0.3. Note that the criteria for declaring a detection in QNO are significantly stricter than those used in PhaseNO in this example.

We compare the resulting catalogs using a minimum threshold of ${N}_{{{\rm{QNO}}}}^{signal}={N}_{\,{{\rm{PhaseNO}}}}^{pick}=16$ for both QNO and PhaseNO (Fig. 6 and Supplementary Fig. 19). Additionally, we use ${T}_{{{\rm{QNO}}}}^{signal}=0.6$ or ${T}_{{{\rm{QNO}}}}^{signal}=0.8$ for QNO, and ${T}_{\,{{\rm{PhaseNO}}}}^{pick}=0.3$ for PhaseNO when determining the existence of an earthquake signal or phase. These hyperparameter settings indicate that QNO applies much stricter criteria than PhaseNO. The detection trends over time are consistent between the QNO and PhaseNO catalogs, with the QNO catalog consistently containing more detections, suggesting that most earthquakes in the PhaseNO catalog were also detected by the QNO method. This consistency highlights the effectiveness of QNO in constructing accurate earthquake catalogs. Reducing QNO’s threshold from 0.8 to 0.6 results in 10,167 detections, nearly one order of magnitude higher than the 874 earthquakes listed in the NCEDC catalog for the same period. When an event is detected based on the classification results and predefined thresholds, we retain the corresponding regression results as the location for that event. The locations of these earthquakes detected by QNO align with those detected by PhaseNO + GaMMA and in the NCEDC catalog (Fig. 6 and Supplementary Fig. 20), suggesting that most of these additional detections are meaningful rather than false positives. This comprehensive catalog, built with QNO, has great potential to reveal rich subsurface information about the status of the geothermal system.

We further assess the consistency among catalogs with a cross-validation test, treating one catalog as the ground truth and evaluating the relative performance of the others (Table 1). Since the true number of events within these continuous waveforms is unknown and each catalog may contain false positives and false negatives due to differences in algorithms, hyperparameters, and thresholds, accurately comparing detection performance across catalogs is challenging. To facilitate comparison, earthquake events were considered true positives if their origin times fell within 3 s of an event in the benchmark catalog. With recall scores of 0.96 or 0.97, we observe that almost all NCEDC events were detected by both methods. The F1 score (which balances recall and precision) between QNO and PhaseNO+GaMMA is 0.80, and is similarly high for QNO at different thresholds. This indicates strong consistency between PhaseNO and QNO, even with QNO applying a much stricter standard for catalog building.

**Table 1:** Cross-validation among earthquake catalogs for the Geysers geothermal field

Test catalog	Metric	Benchmark catalog			
		NCEDC	PhaseNO +GaMMA	QNO (0.8)	QNO (0.6)
**NCEDC**	**Recall**	1.00	0.16	0.14	0.10
	**Precision**	1.00	0.96	0.97	0.97
	**F1 score**	1.00	0.27	0.24	0.18
**PhaseNO+GaMMA**	**Recall**	0.96	1.00	0.74	0.59
	**Precision**	0.16	1.00	0.86	0.93
	**F1 score**	0.27	1.00	0.80	0.72
**QNO (0.8)**	**Recall**	0.97	0.86	1.00	0.73
	**Precision**	0.14	0.74	1.00	1.00
	**F1 score**	0.24	0.80	1.00	0.84
**QNO (0.6)**	**Recall**	0.97	0.93	1.00	1.00
	**Precision**	0.10	0.59	0.73	1.00
	**F1 score**	0.18	0.72	0.84	1.00

Parenthetic values indicate the probability threshold used to classify a recording at one station as containing an earthquake signal.

**Fig. 7:**
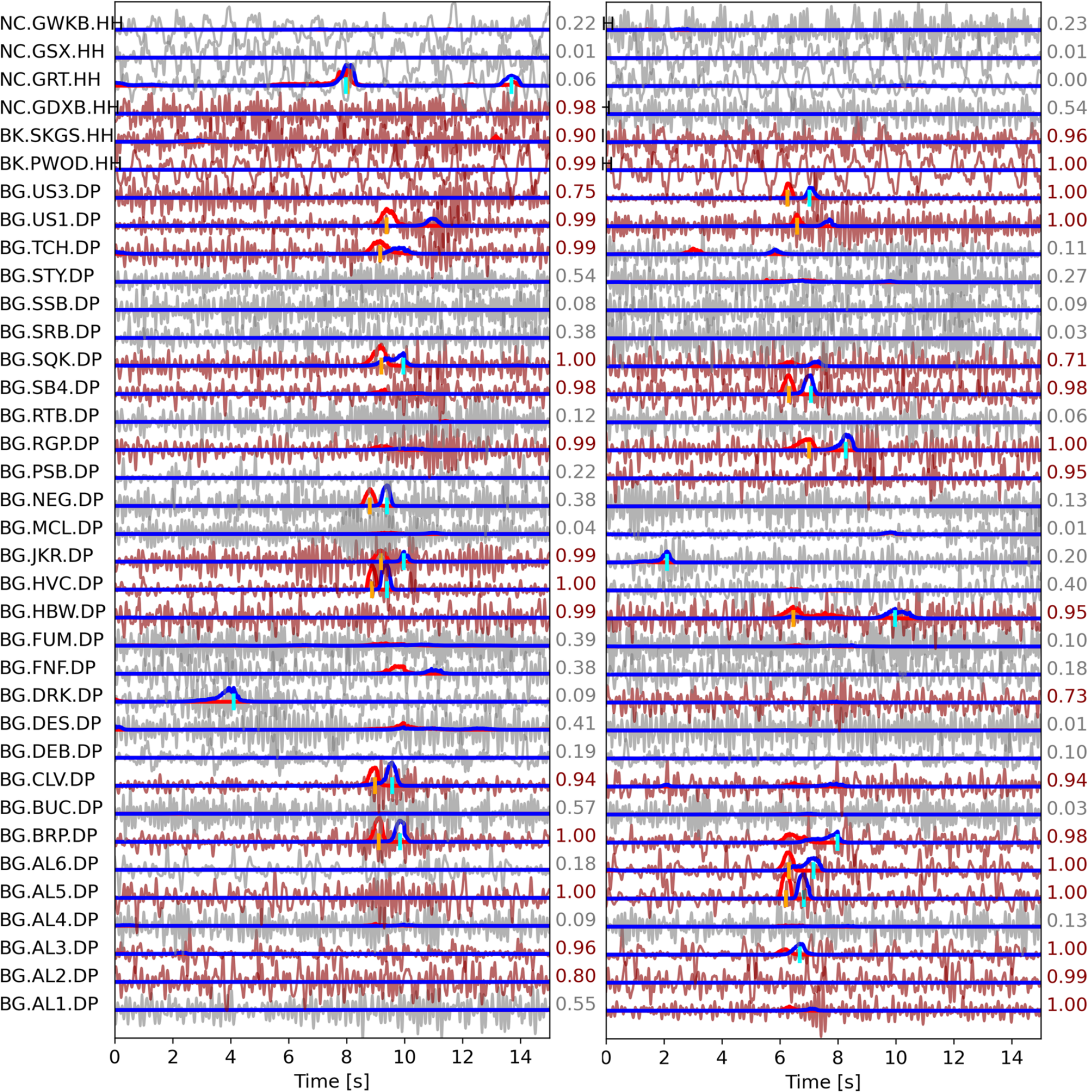
Comparison of detection performance between QNO and PhaseNO+GaMMA for two microseismic events. Waveforms of all input stations are plotted. The signal probability ${p}_{i}^{\,{{\rm{signal}}}}$ predicted by QNO is shown at the end of each waveform. Waveforms are highlighted in red if ${p}_{i}^{\,{{\rm{signal}}}} > 0.7$, indicating detection of an earthquake signal at that threshold. The probability curves predicted by PhaseNO are plotted in red for P-phases and in blue for S-phases. Vertical bars indicate seismic phases determined when the probabilities are larger than 0.3. In the left and right panels, PhaseNO did not find picks on 10 and 8 stations, respectively, whose waveforms were classified by QNO as containing earthquake signals. If we require both QNO and PhaseNO+GaMMA to have a minimum threshold of ${N}_{{{\rm{QNO}}}}^{signal}={N}_{\,{{\rm{PhaseNO}}}}^{pick}=16$ for a valid detection, then these events are overlooked by PhaseNO+GaMMA. In contrast, QNO detected these events and directly determined their locations from these input waveforms. More examples can be found in the Supplementary Figs. 21–29.

**Fig. 8:**
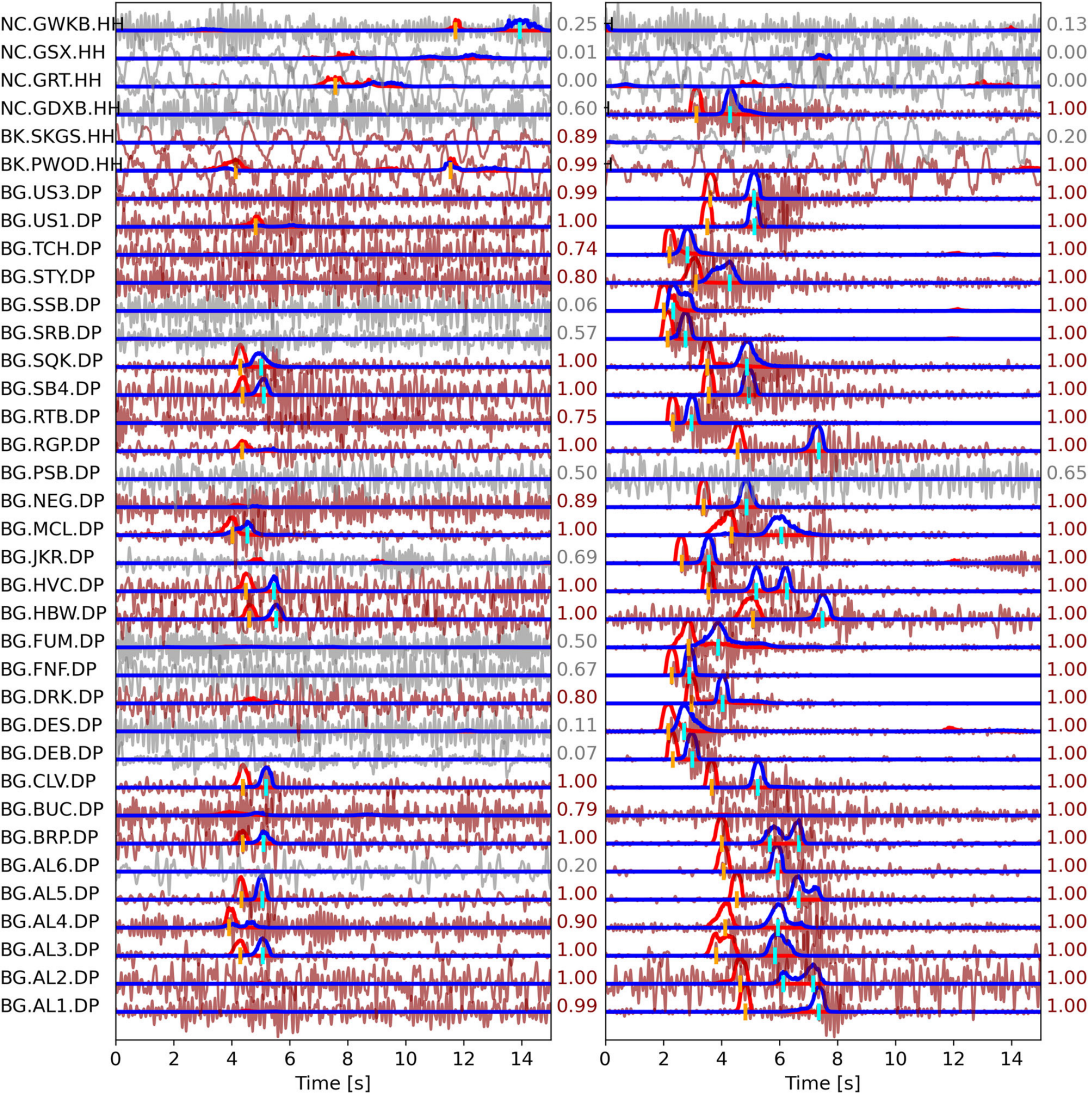
Two seismic events detected by both the QNO and PhaseNO+GaMMA methods. For the event with a relatively high SNR (right panel), QNO and PhaseNO produce consistent detection results. In contrast, for the event with a lower SNR (left panel), QNO detects signals on more stations compared to PhaseNO.

The comparison of probability functions predicted by QNO and PhaseNO further highlights QNO’s advantage in microseismic monitoring (see Figs. 7,8 and Supplementary Figs. 21–29). Figure 7 presents two representative microseismic events detected by QNO with thresholds of ${T}_{{{\rm{QNO}}}}^{signal}=0.7$ and ${N}_{{{\rm{QNO}}}}^{signal}=16$. Waveforms are plotted at all input stations, showing that the environmental noise is very strong, leaving few clear onsets of the seismic phase. QNO successfully identified 17 earthquake signals out of 36 input stations for both events. In contrast, PhaseNO only made picks on a few input waveforms with ${T}_{\,{{\rm{PhaseNO}}}}^{pick}=0.3$. These low-quality events detected by PhaseNO are filtered out during association using a threshold of ${N}_{\,{{\rm{PhaseNO}}}}^{pick}=16$. Even if the detection could be recovered by lowering the association threshold, the uncertainties in phase picking caused by strong noise would still propagate to the subsequent location step.

Figure 8 presents two additional seismic events detected by both the QNO and PhaseNO methods. For the event with a relatively high SNR, QNO achieves detection results consistent with PhaseNO. Conversely, for the event with a lower SNR, QNO identifies signals on more stations than PhaseNO. Additional examples are provided in the Supplementary Figs. 21–29.

### Computational Cost of QNO

**Fig. 9:**
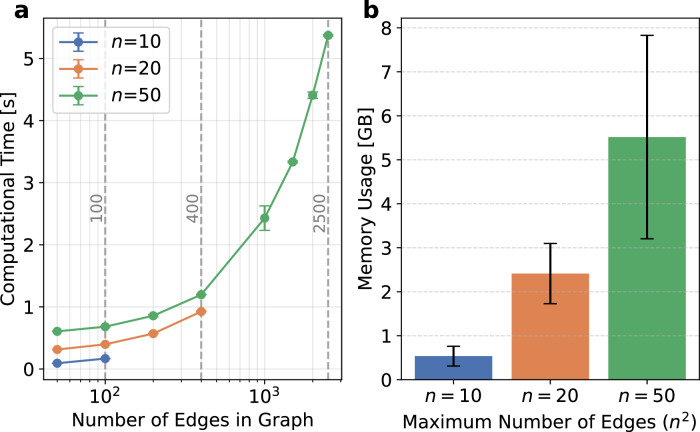
QNO’s computational cost of one prediction during testing, evaluated on an Apple M3 Max using CPU with two threads, with results averaged over six runs. **a** Computational time (mean  ± standard deviation) as a function of the number of edges in the graph for different numbers of stations *n*. Each curve corresponds to a fixed *n*, and dashed vertical lines indicate the maximum possible number of edges *n*^2^ for that configuration. **b** Memory usage (mean  ± standard deviation) for graphs at the maximum number of edges *n*^2^ for *n* = 10, 20, and 50, reported in gigabytes.

We investigate the capability of using QNO for real-time monitoring. Although training QNO is time-consuming, once the model is well-trained, it can be directly applied to monitor the area of interest. We evaluate the computational cost of one prediction during testing on an Apple M3 Max with results averaged over six runs (Fig. 9). The computational cost is reported for different numbers of stations and graph configurations using only the CPU with two threads. The computational time and memory usage largely depend on the number of edges and stations in a single input sample. For example, a network with *n* = 20 stations forming a fully connected directed graph with self-loops (20 × 20 = 400 edges) requires  ≈ 1 s per 15 s input sample and about 2.4 GB of memory. This represents the maximum computational cost for the 20-station case, which can be reduced by decreasing the number of edges. Even with 50 stations, the maximum computational cost is around 5.5 s per 15 s input sample with a memory usage of about 5.5 GB. For a real-time data stream where the system waits 10 s before processing the next prediction (with 5 s overlap between two predictions), this 5.5 s computational time allows QNO to be deployed directly for real-time monitoring using a CPU with two threads.

For a given seismic network with a fixed number of stations and geometry, the number of edges is determined by the graph configuration in the GNO layer, which is constructed based on the physical distance between stations (see Supplementary Note 1 and Supplementary Fig. 3). For a given station, edges are created between that station and any other station (including itself) if the distance between the two is less than or equal to a physical distance threshold *D*. A *D* value larger than the maximum distance among stations results in a fully connected graph with a total of *n*^2^ edges, which requires the highest computational cost. In practice, for large networks one can choose a relatively small *D* to reduce computational cost while preserving effective inter-station communication, as evidenced by generalization when training with *D* = 40 km and testing with *D* = 20 km and *D* = 60 km (Supplementary Fig. 30). For the 50-station network evaluated in Fig. 9, the computational cost decreases substantially when the number of edges is reduced, which further enables the deployment of a trained QNO model for real-time monitoring.

## Discussion

QNO is a multi-task operator learning framework that integrates one classification task for earthquake detection and one regression task for earthquake location. Both tasks are jointly addressed by sharing the underlying neural operator structures, effectively solving the seismic monitoring problem, where earthquake detection and location are closely related. By combining FNO for temporal feature extraction and GNO for spatial information exchange, QNO efficiently handles the complex structure of seismic network data. Furthermore, it can process seismic data from networks with varying geometries while maintaining a fixed model architecture.

Additionally, unlike other multi-station algorithms that fully encode seismic waveforms at each individual station before exchanging information among stations^50^, QNO facilitates communication among stations throughout the entire data flow within the neural operator. The sequential connection of FNO and GNO, along with the repeated application of these layers, ensures a comprehensive exchange of spatiotemporal information, enhancing earthquake detection and location accuracy.

Picking-based seismic monitoring workflows identify seismic arrivals from continuous data and then associate these picks with seismic events. Typically, a minimum number of picks is set as a hyperparameter, and any association results with fewer picks than this threshold are filtered out. In cases of microseismicity, where only a few clear picks are detected across the seismic network, these events can easily be excluded by such filtering criteria. By contrast, QNO searches for the waveform information of a seismic event across multiple stations without picking and thus can detect small-magnitude events effectively. At the same time, the location of these events is determined directly from waveforms, reducing potential location errors due to post-processing steps.

Here, we clarify how QNO differs from operator-learning approaches for phase picking, i.e., PhaseNO^19^. PhaseNO is formulated as a per-station picking task that outputs pseudo-probability distributions of P/S arrival times and relies on labeled picks for supervision; its outputs are station-level and are typically passed to a downstream associator or locator. In contrast, QNO is an end-to-end model that operates directly on multi-station waveforms without picking, jointly learning station-level detection (classification) and event-level location (regression) under a single multi-task objective. Architecturally, PhaseNO employs a U-shaped operator tailored to produce time-aligned station outputs, whereas QNO adopts a simpler sequential backbone with a lower memory footprint and shorter runtime, enabling near-real-time monitoring even on CPUs. We view QNO as complementary to pick-based monitoring pipelines: where high-quality picks are available, they remain useful; where onsets are emergent or noisy, and picks are unreliable, QNO enables direct cataloging from continuous waveform data.

Again, PhaseNO and QNO are not necessarily replacements for one another. Using them jointly can enhance both the detection rate and the reliability of detected events. The picked arrivals from PhaseNO, combined with the initial location results from QNO, allow for further optimization of event locations using the double-difference location algorithm^61^. PhaseNO excels in handling very early aftershock periods following large mainshocks where multiple events occur in a short time window and earthquake signals have relatively large amplitudes for PhaseNO to pick arrivals. In contrast, QNO is more effective for microseismicity, where phase onsets are unclear, and seismicity rates are relatively low. A small fraction (5.36%) of PhaseNO-catalog events in the Geysers geothermal dataset occur within 10 s of a preceding event and may be missed by QNO (Supplementary Fig. 31); however, this trade-off may be acceptable given QNO’s improved overall microseismic detection performance. Additionally, while the deep-learning-based picking algorithm is highly generalizable across different regions for picking seismic events^62,63^, location algorithms typically require local events in the training dataset to account for regional velocity structures.

Ideally, QNO is designed to process one event per input time window. To investigate QNO’s behavior when multiple earthquakes occur within the same input window, we constructed synthetic two-event cases by stacking real events from the NCEDC test dataset and varying both the amplitude ratio *r* and the inter-event delay time Δ between them (Supplementary Note 3). The delay time was defined as the difference between the earliest P-wave arrival times across all stations for the two events. We find that QNO still produces high signal probabilities at nearly all stations and returns a single location estimate for the input window, even when some stations lack clear phase picks for both events. We then compared this predicted location with catalog locations of both events to determine which was actually located (Supplementary Figs. 32 and 33). Across tested combinations of *r* and Δ, QNO consistently located the earlier-occurring event (Event 1), with location errors for Event 1 being smaller and less sensitive to *r* and Δ than those for Event 2. In contrast, Event 2’s errors increased as Δ decreased or as its relative amplitude weakened, likely due to reduced visibility of its signals on the input waveforms. In addition, the azimuthal gap of Event 2 in Supplementary Fig. 32 is larger than that of Event 1, which may also contribute to the increased errors.

Although all numerical examples in this study use 15 s waveforms, this input length is not fixed; QNO can be trained for any waveform duration by adjusting the input layer size. However, for a trained model, the application-stage input length must match the training length, while the input sampling rate may differ. In practice, the waveform window length can be chosen based on the distribution of inter-event times derived from a high-resolution earthquake catalog of historical seismicity. Then separate QNO models could be trained for different time windows, for example, using a shorter window when seismicity rates are high to minimize the likelihood of multiple events occurring within the same input window. Moreover, when applied to continuous data, using overlapping predictions between successive time windows can help prevent missing events, as demonstrated in our case study of the Geysers geothermal field.

While shorter time windows are effective in minimizing the occurrence of multiple events within a single window, which is a key consideration for microseismic monitoring, they may lead to repeated detections of long-duration seismic events. When the rupture duration or significant energy release of an earthquake exceeds the predefined time window, the algorithm may trigger detections in consecutive windows. In the Geysers case study, out of 10,167 raw detections, approximately 18% occurred within 5 s of a preceding detection. These detections may include both temporally close natural events and repeated detections of the same event. While such repeated detections reflect the robustness of the QNO classification and regression operators in capturing different stages of the same event (e.g., codas or late-arriving phases), they may introduce duplicate entries in the resulting catalog. For practical applications, these duplicates could be addressed through standard post-processing clustering. For example, by simply merging detections within a temporal proximity of 5 s, we obtained a refined catalog of 8346 events from the QNO detections (with a probability threshold of 0.6), which still represents a nearly ten-fold increase compared to routine monitoring results.

## Method

We propose QNO, a multi-task neural operator designed for earthquake detection and location directly on continuous seismic waveform data without explicit phase picking or phase association. This section provides an overview of the QNO, including its architecture, loss function, and training dataset.

### Quake neural operator in multi-task learning

QNO performs a nonlinear mapping from the input function *f*(*t*; *x*, *y*, *z*) to the output in a multi-task learning framework. It consists of a shared feature extractor ${{\mathcal{F}}}$ followed by separate classification ${{{\mathcal{Q}}}}_{{{\rm{class}}}}$ and regression ${{{\mathcal{Q}}}}_{{{\rm{reg}}}}$ branches:

1 $${{\bf{h}}}={{\mathcal{F}}}(f(t;x,y,z)),$$

2 $${{\mathcal{G}}}(f(t;x,y,z))=\left({{{\mathcal{Q}}}}_{{{\rm{class}}}}({{\bf{h}}}),{{{\mathcal{Q}}}}_{{{\rm{reg}}}}({{\bf{h}}})\right),$$

where **h** represents the shared feature extracted from the input function with a nonlinear operator ${{\mathcal{F}}}$. ${{\mathcal{G}}}$ denotes the QNO. For multi-station seismic data, (*x*, *y*, *z*) denotes the spatial coordinates of the seismic stations, while *t* denotes the temporal coordinate. The input samples are the discretized values of this function *f*(*t*; *x*, *y*, *z*) in both space and time. In principle, neural operators ${{\mathcal{F}}}$, ${{\mathcal{G}}}$, ${{{\mathcal{Q}}}}_{{{\rm{class}}}}$ and ${{{\mathcal{Q}}}}_{{{\rm{reg}}}}$ are formulated to operate on inputs defined on arbitrary discretizations^59^. In practice, this allows QNO to be trained and evaluated on varying seismic network geometries.

For earthquake detection, the classification task ${{{\mathcal{Q}}}}_{{{\rm{class}}}}$ processes the shared representation **h** to produce a spatial logits field, which is converted into a probability function *p*(*x*, *y*, *z*) using the softmax function:

3 $$p(x,y,z)=\,{{\rm{softmax}}}\,({{{\mathcal{Q}}}}_{{{\rm{class}}}}({{\bf{h}}})).$$

The probability function is evaluated at the station locations. Here, ${{\bf{p}}}={\{{{{\bf{p}}}}_{i}\}}_{i=1}^{n}\in {{\mathbb{R}}}^{n\times 2}$ denotes the probabilities for earthquake signals and noise at stations located at (*x*_*i*_, *y*_*i*_, *z*_*i*_), where *i* ∈ {1, 2, …, *n*} and *n* is the number of stations in the input. Each ${{{\bf{p}}}}_{i}\in {{\mathbb{R}}}^{2}$ represents the probability distribution over signal and noise for the *i*-th station:

4 $${{{\bf{p}}}}_{i}=\left[{p}_{i}^{\,{{\rm{signal}}}},{p}_{i}^{{{\rm{noise}}}}\right],\,{p}_{i}^{\,{{\rm{signal}}}}+{p}_{i}^{{{\rm{noise}}}}=1.$$

For earthquake location, the regression task outputs the earthquake source location $(\widehat{x},\widehat{y},\widehat{z})$ and origin time $\widehat{t}$:

5 $$\widehat{{{\bf{r}}}}={{{\mathcal{Q}}}}_{{{\rm{reg}}}}({{\bf{h}}}),$$

where $\widehat{{{\bf{r}}}}=(\widehat{x},\widehat{y},\widehat{z},\widehat{t})\in {{\mathbb{R}}}^{4}$ is the predicted result.

### Architecture of quake neural operator

The architecture of QNO (Fig. 1) mainly relies on two types of neural operators, which can efficiently process the spatiotemporal representation of seismic data over time and space (see Supplementary Note 1): One is the Fourier Neural Operator (FNO)^56^ and the other is the Graph Neural Operator (GNO)^57^. FNO learns global correlations in the temporal axis using Fourier transforms, effectively capturing long-range dependencies in the data. GNO operates on graph structures to model relationships among seismic stations, which effectively deals with the irregular sampling of seismic data in the spatial domain. Started from the input function *f*(*t*; *x*, *y*, *z*), the neural operator ${{\mathcal{F}}}$ shared by both the classification and regression tasks can be viewed as a series of mappings through layers of operation:

6 $${{\bf{h}}}={{{\rm{FNO}}}}_{k+2}\circ {{{\rm{FNO}}}}_{k+1}\circ {{{\rm{GNO}}}}_{k}\circ {{{\rm{FNO}}}}_{k}\circ \cdots \circ {{{\rm{GNO}}}}_{1}\circ {{{\rm{FNO}}}}_{1}\circ {{\mathcal{P}}}(f(t;x,y,z)),$$

where ${\circ }$ denotes the mapping between two layers. With *k* = 3, QNO uses three combinations of layers of FNO and GNO. The input function is first passed through an up-projection layer ${{\mathcal{P}}}$, which maps the input function to a high-dimensional representation. Then the up-projected data is passed through three combinations of FNO and GNO layers, allowing sufficient exchange of information between the time and space domains.

Each FNO layer performs a 1-D spectral convolution along the temporal axis at each station: we apply a Fourier transform to the per-station features, multiply the lowest *M*_*k*_ frequency modes by learned complex weights while zeroing higher modes, inverse-transform back to the time domain, and then combine the result with a pointwise (1 × 1) linear projection, followed by a nonlinearity^56^. To reduce boundary artifacts associated with applying Fourier transforms to non-periodic time series, we apply zero-padding (50 samples on each end) prior to the FNO layers. This suppresses spurious high-frequency components induced by discontinuities at the signal boundaries and stabilizes the spectral representation used by the operator layers. In addition, each FNO layer retains only the first *M*_*k*_ lowest-frequency modes, as high-frequency components are more difficult to learn and are truncated during training^56^. The number of modes in each FNO layer is 24, 12, 8, 8, and 8, respectively. The width (or channel number) of the discretized function at each station varies with the dimension. Across the FNO layers, the per-station discretized representation $v(t;{{{\bf{x}}}}_{i})\in {{\mathbb{R}}}^{{C}_{k}\times {T}_{k}}$, with **x**_*i*_ = (*x*_*i*_, *y*_*i*_, *z*_*i*_), takes the following shapes by layer: 48 × 1500, 96 × 500, 192 × 100, 192 × 50, and 24 × 50. The first dimension is the channel width *C*_*k*_; the second is the number of time samples *T*_*k*_. As the network progresses through downsampling, the number of Fourier modes *M*_*k*_ is reduced in proportion to the compressed resolution, while the channel dimensions are increased to enrich feature representations. Nonlinearity is introduced in all FNO layers using the Gaussian Error Linear Unit^64^, which applies a smooth, probabilistic gating mechanism that approximates the input multiplied by the cumulative distribution function of a standard normal distribution. The output of the last FNO layer is flattened into 1200 channels before feeding into ${{{\mathcal{Q}}}}_{{{\rm{class}}}}$ and ${{{\mathcal{Q}}}}_{{{\rm{reg}}}}$.

At each GNO layer, stations are treated as nodes in a graph, and their features are updated through a message-passing framework^65^. The graph is constructed in the input spatial domain using a distance threshold *D* (two stations are connected with an edge if their pairwise distance ≤*D*). Unless otherwise noted, for example, when explicitly analyzing generalization with respect to *D* (see Supplementary Fig. 30), we set *D* = 40 km. Per-station temporal features $v(t;{{{\bf{x}}}}_{i})\in {{\mathbb{R}}}^{{C}_{k}\times T_{k}}$ produced by the preceding FNO layer serve as node features. For each edge (*i*, *j*), an edge message is computed by a differentiable map *φ* that takes the two node features concatenated along the temporal axis as the input, ${m}_{ij}=\varphi \,\left(v({{{\bf{x}}}}_{i}),v({{{\bf{x}}}}_{j})\right)$. Node *i* then performs mean aggregation ${\bar{m}}_{i}=\frac{1}{| {{\mathcal{N}}}({{{\bf{x}}}}_{i})| }{\sum }_{j\in {{\mathcal{N}}}({{{\bf{x}}}}_{i})}{m}_{ij}$ and updates its representation via a second map *ψ*: $u({{{\bf{x}}}}_{i})=\psi \,\left(v({{{\bf{x}}}}_{i}),{\bar{m}}_{i}\right)$. In QNO, both *φ* and *ψ* are two-layer MLPs with hidden width 4*C*_*k*_, where *C*_*k*_ is the channel dimension of the node features output by the *k*-th FNO layer preceding the *k*-th GNO layer. The message-passing framework in the GNO layer is permutation-invariant, accommodates irregular station layouts, and couples spatial communication with the temporal representations learned by the FNO. QNO uses three GNO layers interleaved with FNO layers.

The final output **h** from the shared part branches into two separate parts for classification and regression, respectively. To reduce the dimensionality of the shared feature representation **h**, we use two different down-projection layers for the separated branches of the regression and classification tasks. The classification output is generated by passing **h** through the down projection layer ${{{\mathcal{Q}}}}_{{{\rm{class}}}}$ and applying the softmax function. The regression output is generated by passing **h** through the down-projection layer ${{{\mathcal{Q}}}}_{{{\rm{reg}}}}$, producing the predicted earthquake source location and origin time. Both ${{{\mathcal{Q}}}}_{{{\rm{class}}}}$ and ${{{\mathcal{Q}}}}_{{{\rm{reg}}}}$ are two-layer fully connected neural networks.

### Loss function for the multi-task learning framework

The total loss function of the multi-task neural operator is a weighted sum of the individual task losses:

7 $${{{\mathcal{L}}}}_{{{\rm{total}}}}={{{\mathcal{L}}}}_{{{\rm{class}}}}+\alpha {{{\mathcal{L}}}}_{{{\rm{reg}}}},$$

where *α* is a weight that balances the contribution of the regression task relative to the classification task. ${{{\mathcal{L}}}}_{{{\rm{class}}}}$ is the classification loss; and ${{{\mathcal{L}}}}_{{{\rm{reg}}}}$ is the regression loss. Here we choose *α* = 1.

The cross-entropy loss function for the classification task is an expectation over the joint distribution $(X,Y) \sim {{{\mathcal{D}}}}_{1}$, where *X* denotes evaluations of the seismic wavefield function *f*(*t*; *x*, *y*, *z*) at the station locations of a seismic network, and *Y* = [*Y*^signal^, *Y*^noise^] denotes the corresponding labels:

8 $${{{\mathcal{L}}}}_{{{\rm{class}}}}={{\mathbb{E}}}_{(X,Y) \sim {{{\mathcal{D}}}}_{1}}\left[-{\sum }_{i=1}^{n}\left({Y}_{i}^{\,{{\rm{signal}}}}\log {p}_{i}^{{{\rm{signal}}}}+{Y}_{i}^{{{\rm{noise}}}}\log {p}_{i}^{{{\rm{noise}}}}\right)\right].$$

The regression task uses mean squared error (MSE) to predict the source parameters of an earthquake $\widehat{{{\bf{r}}}}=(\widehat{x},\widehat{y},\widehat{z},\widehat{t})$:

9 $${{{\mathcal{L}}}}_{{{\rm{reg}}}}={{\mathbb{E}}}_{(X,{{{\bf{r}}}}_{{{\rm{true}}}}) \sim {{{\mathcal{D}}}}_{2}}\left[{(\widehat{x}-{x}_{{{\rm{true}}}})}^{2}+{(\widehat{y}-{y}_{{{\rm{true}}}})}^{2}+{(\widehat{z}-{z}_{{{\rm{true}}}})}^{2}+{(\widehat{t}-{t}_{{{\rm{true}}}})}^{2}\right],$$

where **r**_true_ = (*x*_true_, *y*_true_, *z*_true_, *t*_true_) contains the ground-truth earthquake source locations and origin times. These labels usually come from an existing earthquake catalog used to construct the training dataset.

### Training dataset

We use a training dataset previously employed to train two deep neural phase pickers, PhaseNet^11^ and PhaseNO^19^. Using a similar training dataset enables a fair comparison and evaluation of the performance of each method after training. The dataset consists of three-component earthquake waveforms and event catalogs spanning 30 years from the Northern California Earthquake Data Center (NCEDC). To train QNO for microseismic monitoring, we focus on events with magnitudes up to 2. To improve accuracy and minimize uncertainties, we select events recorded by at least eight stations. The event depths range from -4 to 36 km (Fig. 2). Depths are referenced to sea level; negative values indicate earthquakes occurring above sea level in high-elevation regions (e.g., mountains in California).

We adopt a temporal split as the primary setting: events prior to 2016 form the training set, while events from 2016 and 2017-2021 are designated as the validation and test sets, respectively. This selection yields 7972 events in the training set, 1402 events in the validation set, and 1019 events in the test set. This design reflects practical deployment, where models trained on historical earthquakes are applied to future events; following common practice, this temporal split implicitly disregards the question of stationarity. It also introduces temporal distribution shift due to changes in station coverage, noise, and catalog completeness. Unless otherwise specified, all evaluations use the model trained on events before 2016. For a complementary random split and additional analyses, see Supplementary Note 4 and Supplementary Figs. 4 and 5.

We perform data augmentation and pre-processing when preparing the training dataset. In the NCEDC dataset downloaded from the data center, waveforms from all stations associated with an event contain earthquake signals. However, in real-world scenarios, not all stations record earthquake waveforms. To simulate this, we generate up to 16 virtual stations with random locations within the computational domain and assign noise waveforms to these virtual stations. Each station is assigned a classification label: [1, 0] for earthquake signals and [0, 1] for noise, where the first channel indicates ${p}_{i}^{signal}$ and the second channel represents ${p}_{i}^{noise}$. To improve QNO’s ability to detect events in the presence of strong noise, we also added noise to the earthquake waveforms at real stations. The noise waveforms were randomly selected from the 235,000 noise samples available in the STEAD dataset^58^, and the noise varies across different stations. Then these raw waveforms were first preprocessed by removing the trend using a demeaning procedure and applying a bandpass filter with a frequency range of 1 to 10 Hz. After filtering, the waveforms were normalized by dividing each channel by its maximum value.

We use a relatively short time window of 15 s to train QNO. This window length is appropriate for local microseismic monitoring where waves decay fast during propagation. A short time also reduces the possibility of the existence of multiple events in one sample. Since QNO faces the challenge of handling multiple events within a single input time window (see Supplementary Note 3), selecting a short time window is a straightforward way to address this issue and is particularly effective for microseismic monitoring, where inter-event times are generally much longer than those in aftershock sequences of large earthquakes. Moreover, we use overlapping time windows when processing continuous data, which helps to reduce the possibility of missing events.

We construct a computational domain that can cover the locations of the local earthquake and stations for each sample in the dataset. Coordinates on the computational domain have values between 0 and 1, which is favorable for the learning process. The physical lower bounds of longitude *λ*_0_ and latitude *ϕ*_0_ for the computational domain are given by:

10 $${\lambda }_{0}=\frac{{\lambda }_{{{\max }}}+{\lambda }_{\min }}{2}-\frac{d}{2},$$

11 $${\phi }_{0}=\frac{{\phi }_{{{\max }}}+{\phi }_{\min }}{2}-\frac{d}{2},$$

where *λ*_max_ is the maximum longitude, ${\lambda }_{\min }$, the minimum longitude, *ϕ*_max_, the maximum latitude, ${\phi }_{\min }$, the minimum latitude of all stations around an earthquake. Each sample is mapped with a varying center so that all stations in the graph are around the middle of the computational domain. In addition, *d* represents the extent of the computational domain on the Earth’s surface. The chosen range *d* should be large enough to encompass all the stations within the graph. Since the propagation range of small-scale events is typically short, a selection of *d* = 1. 2° is sufficient and appropriate for monitoring local earthquakes in our experiments. After determining the physical lower bounds of the computational domain, we can calculate the relative position of each station within this domain:

12 $${x}_{i}=\frac{{\lambda }_{i}-{\lambda }_{0}}{d},$$

13 $${y}_{i}=\frac{{\phi }_{i}-{\phi }_{0}}{d},$$

14 $${z}_{i}=\frac{{\eta }_{i}-{\eta }_{min}}{{\eta }_{max}-{\eta }_{min}},$$

where *λ*_*i*_, *ϕ*_*i*_, and *η*_*i*_ are respectively the longitude, latitude, and depth of the i-th station. *η*_max_ is the maximum depth and ${\eta }_{\min }$ is the minimum depth of the computational domain. We choose ${\eta }_{\min }=-4$ km and *η*_max_ = 36 km to include the depth of all earthquakes in the dataset. The computational domain and the relative positions (*x*_*i*_, *y*_*i*_, *z*_*i*_) of the stations are computed independently for each data sample during training. For real-world scenarios, the relative positions are computed only once for a given seismic network. These transformed coordinates are treated as node attributes and three additional channels of the input, along with the three-component waveform information.

Similarly, the regression label for earthquake location is the earthquake’s relative location (*x*_*true*_, *y*_*true*_, *z*_*true*_) in the computational domain:

15 $${x}_{true}=\frac{\Lambda -{\lambda }_{0}}{d},$$

16 $${y}_{true}=\frac{\Phi -{\phi }_{0}}{d},$$

17 $${z}_{true}=\frac{H-{\eta }_{min}}{{\eta }_{max}-{\eta }_{min}},$$

where (*Λ*, *Φ*, *H*) denotes the ground-truth earthquake location, which in practice is usually taken from the earthquake catalog. The time predicted by QNO is the occurrence time of an event relative to the start of the input time series. Assuming the origin time *T* is within a range of 10 s earlier (*t*_*min*_ = − 10 s) and 10 s later (*t*_*max*_ = 10 s) than the starting time of an input waveform, the time *t*_*true*_ of the regression label that we train QNO to predict should be:

18 $${t}_{true}=\frac{T-{t}_{min}}{{t}_{max}-{t}_{min}}.$$

We train QNO with the Adaptive Moment Estimation (Adam) optimizer^66^ with a batch size of one and a learning rate of 10^−4^. Adam is a stochastic gradient-based optimizer that computes adaptive learning rates for each parameter based on estimates of first and second moments of the gradients. The training takes around three hours for one epoch on one NVIDIA Tesla V100 GPU. We train the model with 19 epochs and then evaluate it on the test dataset and 10-day continuous data from the Geysers geothermal field.

When evaluating performance on the test dataset (Fig. 4), we first convert the predicted coordinates $(\widehat{x},\widehat{y},\widehat{z},\widehat{t})$ of each sample to physical coordinates corresponding to longitude, latitude, depth, and origin time. We then compute the differences between the converted predictions and the corresponding catalog values (*Λ*, Φ, *H*, *T*). Location errors in degrees are converted to kilometers using a factor of 111 km/° for latitude and approximately 88 km/° for longitude, based on the mean latitude of the study area.

## Supplementary information

Supplementary Information

Transparent Peer Review file

## Source data

Source Data

## Data Availability

All waveform data are publicly available from the Northern California Earthquake Data Center (NCEDC), doi:10.7932/NCEDC^67^. The NCEDC catalog for the Geysers geothermal data is publicly available at NCEDC (last access: August 1, 2025). Source data are provided with this paper.

## Code availability

The code developed in this study for the Quake Neural Operator is publicly available on GitHub at https://github.com/sun-hongyu/QNOand archived on Zenodo at 10.5281/zenodo.20072888^68^. The PhaseNO software is available at https://github.com/sun-hongyu/PhaseNO. The SeismicGNN software is available at https://github.com/martijnende/SeismicGNN.
