# Supplementary material for: Microseismic monitoring with the quake neural operator
Source: Nat Commun. 2026 Jul 6;17:5677. doi: 10.1038/s41467-026-73965-6 (PMC13338072; doi:10.1038/s41467-026-73965-6)
Supplement: Supplementary file 1 — Supplementary Information [file 41467_2026_73965_MOESM1_ESM.pdf]

Supplementary Information for

"Microseismic Monitoring with the Quake Neural Operator"

Hongyu Sun[1,2,*]

[1]Department of Earth, Environmental and Resource Sciences,
The University of Texas at El Paso, El Paso, TX, USA

[2]School of Earth Sciences,
The Ohio State University, Columbus, OH, USA

[*]Corresponding author. Email: hongyu-sun@outlook.com

Contents

Supplementary Note 1 Preliminaries on Neural Operators.

Supplementary Note 2 Additional examples comparing QNO and PhaseNO.

Supplementary Note 3 Multi-event superposition test for QNO

Supplementary Note 4 Comparison of data-splitting strategies for the NCEDC dataset

Supplementary Fig. 1 Example of a training sample (event nc103327).

Supplementary Fig. 2 Example of a training sample (event nc109829).

Supplementary Fig. 3 Illustration of graph construction in the GNO layer based on a geographic distance threshold D.

Supplementary Fig. 4 NCEDC seismic dataset split using randomly shuffled indices.

Supplementary Fig. 5 Performance of QNO on the NCEDC test dataset split using uniformly random indices.

Supplementary Fig. 6 Comparison of kernel density estimates (KDEs) between QNO predictions and the NCEDC catalog on the test dataset.

Supplementary Fig. 7 Zoom-in view of earthquake locations in a selected region of Northern California.

Supplementary Figs. 8–16 QNO monitoring performance for samples in the NCEDC test dataset under different noise levels.

Supplementary Fig. 17 Detection and location performance for event nc73384250 under varying Gaussian noise levels.

Supplementary Fig. 18 Generalization to higher temporal resolution for QNO trained with 1,500 samples per 15 s input on the NCEDC test dataset.

Supplementary Fig. 19 Hourly earthquake occurrence rates from four catalogs of the Geysers geothermal dataset.

Supplementary Fig. 20 Comparison of KDEs between QNO predictions and the NCEDC catalog on the Geysers geothermal dataset.

Supplementary Figs. 21–29 Comparison of detection performance between QNO and PhaseNO for two microseismic events in the Geysers geothermal field.

Supplementary Fig. 30 Generalization performance of QNO evaluated on the NCEDC test dataset with respect to the geographic distance threshold in the GNO layers.

Supplementary Fig. 31 The temporal variation of time intervals between consecutive earthquakes (inter-event times) in the Geysers geothermal catalog constructed using PhaseNO.

Supplementary Figs. 32–33 Performance of QNO for two example events from the test dataset within a 15 s time window under varying amplitude ratios and delay times.

Supplementary Table 1 List of stations used in the Geysers geothermal field dataset.

Supplementary Note 1: Preliminaries on Neural Operators

Neural operators generalize neural networks to learn mappings between functions defined over infinite-dimensional spaces. Unlike standard networks that map finite-dimensional vectors, neural operators approximate nonlinear operators and are grounded in a universal approximation theorem tailored to operator learning [1]. A typical neural operator layer takes the form of an integral operator:

$$u(\mathbf{x}) = (\kappa * v)(\mathbf{x}) = \int \kappa(\mathbf{x}, \mathbf{y}) v(\mathbf{y})\, d\mathbf{y}, \tag{1}$$

where v and u are input and output functions, and κ is a learnable kernel. The coordinates $\mathbf{x}$ and $\mathbf{y}$ represent domain variables.

Although neural operators were originally developed for solving PDEs, we adapt them for seismic monitoring. If the seismic wavefield is used directly as the input function, the domain is space–time; therefore both $\mathbf{x}$ and $\mathbf{y}$ lie in $\mathbb{R}^4$, each comprising three spatial coordinates and one temporal coordinate. The input function is sampled regularly in the time domain, according to the fixed sampling rate of the recording instruments, and irregularly in the spatial domain, reflecting the non-uniform distribution of seismic station locations. Performing fully coupled four-dimensional integration over space and time is computationally expensive and unnecessary due to the sparse spatial sampling inherent in seismic networks. We therefore decouple the spatiotemporal learning process for seismic monitoring. In this setting, we use $\mathbf{x}_i = (x_i, y_i, z_i)$ to denote the location of the i-th seismic station, and we write the input feature as $v_i(t) = v(t; \mathbf{x}_i)$. The corresponding learned feature after one neural operator layer is $u_i(t) = u(t; \mathbf{x}_i)$. The input to the first neural operator layer is $v_i(t) = \mathcal{P}(f_i(t; \mathbf{x}_i))$, where $\mathcal{P}$ is an up-projection operator applied along the channel dimension that lifts the input seismic data from the original number of channels to a high-dimensional feature space before feature extraction via subsequent neural operator layers. The discretized f_i at each station is a multi-channel, one-dimensional time series; the first three channels are the three-component waveforms. The last three channels encode station coordinates and are constant in t: each channel repeats the corresponding coordinate (x_i, y_i, or z_i) along the time axis, allowing the coordinate channels to be concatenated with the three waveform channels along the channel dimension.

Depending on how the kernel κ in Equation 1 is computed, neural operators can be implemented in several forms. In this work, we integrate two primary variants: Fourier Neural Operators (FNOs) [2] and Graph Neural Operators (GNOs) [3] to construct the architecture of QNO. FNOs are used to extract temporal features at individual stations, while GNOs are employed to learn spatial dependencies among stations. In this formulation, the time-series signal $v_i(t)$ at each station serves as the node feature in a graph, and GNO layers enable information exchange across seismic stations. The resulting QNO architecture alternates between FNO and GNO layers, allowing temporal and spatial representations to be progressively fused across layers (Figure 1).

Fourier Neural Operator

The FNO computes the integral kernel κ in the frequency domain using Fast Fourier Transforms (FFT) [2]. This formulation enables efficient learning of global temporal dependencies

in regularly sampled signals, such as seismic waveforms. According to the convolution theorem, FNO performs a global convolution via spectral multiplication:

$$u_i(t) = \mathcal{F}^{-1}\left(R_\theta \cdot \mathcal{F}(v_i)\right)(t), \tag{2}$$

where $\mathcal{F}$ and $\mathcal{F}^{-1}$ denote the Fourier and inverse Fourier transforms, and R_θ is a learnable spectral kernel.

To improve numerical stability and reduce overfitting, only the first M_k lowest-frequency modes are retained, while the high-frequency modes of the k-th FNO layer are truncated, as they are more prone to noise and harder to learn [2]. A local linear transformation W and a nonlinear activation function σ are further applied to enhance model expressiveness and enable the learning of nonlinear relationships between input and output functions:

$$u_i(t) = \sigma\left(W v_i(t) + \mathcal{F}^{-1}\left(R_\theta \cdot \mathcal{F}(v_i)\right)(t)\right). \tag{3}$$

Each FNO layer thus consists of two branches: one performing global convolution in the frequency domain and the other applying a point-wise local transformation. These are combined before the nonlinear activation function. We use the Gaussian Error Linear Unit (GELU) [4] as the activation function σ.

Graph Neural Operator

To learn spatial dependencies among seismic stations, we represent the irregularly distributed stations as a graph within a GNO framework, where nodes correspond to individual stations. In a GNO layer, two nodes are connected if their pairwise spatial distance in the input domain is smaller than a threshold D. For a given node, the neighboring nodes are therefore defined as all discretized locations lying within a radius D centered at that node, which serves as a discrete approximation to kernel integration over the corresponding local region [3]. In spatial domains such as seismic networks, the number of edges associated with a node (station) corresponds to the number of neighboring seismic stations located within a specified geographic distance. Within this distance threshold, each node is fully connected to its neighboring nodes, including itself through a self-loop. The threshold D is a tunable hyperparameter specified before training. Although seismic stations are irregularly distributed in space, increasing D potentially increases the number of edges in the graph, thereby raising the computational cost due to additional node-to-node communications.

The GNO updates node representations through a message-passing framework [5] similar to graph neural networks (GNN). At each station (node), input features $v(t; \mathbf{x}_i)$ extracted by preceding FNO layers are refined by aggregating information from neighboring stations. For a given node $\mathbf{x}_i$, the updated representation is computed by averaging messages from its neighbors $\mathbf{x}_j \in \mathcal{N}(\mathbf{x}_i)$, and passing the result through a neural network along with the node's own features:

$$u(\mathbf{x}_i) = \psi\left(v(\mathbf{x}_i), \frac{1}{|\mathcal{N}(\mathbf{x}_i)|}\sum_{\mathbf{x}_j \in \mathcal{N}(\mathbf{x}_i)} \varphi(v(\mathbf{x}_i), v(\mathbf{x}_j))\right), \tag{4}$$

where φ and ψ are multilayer perceptrons that encode edge interactions and update node states, respectively.

We construct edge embeddings by applying a differentiable map φ to the features of each connected pair of nodes, i.e., $\varphi\big(v(\mathbf{x}_i),\, v(\mathbf{x}_j)\big)$. Here φ takes the two node representations, concatenated along the temporal axis as the input, and produces a latent edge representation as the message between two connected nodes.

Given the edge messages, each node gathers messages from its neighbors via mean aggregation and then concatenates the aggregated message to its own features $v(\mathbf{x}_i)$ along the temporal axis. The concatenated tensor is processed by a second differentiable map ψ yielding the updated node representation $u(\mathbf{x}_i)$. This message-passing scheme propagates information across neighboring nodes and enables the model to capture spatial relations among seismic stations.

Supplementary Note 2: Additional examples comparing QNO and PhaseNO

This supplementary document provides additional examples demonstrating the performance of QNO in seismic monitoring. These examples emphasize QNO's capability to detect and locate microseismic events under conditions of strong noise and low SNR, which pose challenges for phase-picking-based seismic monitoring workflows.

Supplementary Figs. 8–16 show examples in the test dataset. We evaluate the impact of noise on detection and location performance by adding real noise waveforms to these events in the test dataset while controlling the SNR from 20 dB to -8 dB. In each figure, the upper panel compares the probabilities predicted by QNO and PhaseNO [6] at SNR levels of 20 dB, 10 dB, and 0 dB. The probabilities (p) predicted by QNO for each input station are displayed in red beneath the corresponding station names. The lower panel shows the location error of QNO across different noise levels. Overall, performance degrades as SNR decreases. However, QNO successfully detects the event at most stations even at an SNR of 0 dB and consistently estimates its location and origin time. In contrast, PhaseNO fails to detect the event when the SNR drops to around 0 dB.

Supplementary Figs. 21–29 show examples of events detected by QNO from the Geysers geothermal field. Waveforms of all input stations are plotted. The signal probability p_i^{signal} predicted by QNO is shown at the end of each waveform. Waveforms are highlighted in red if $p_i^{\text{signal}} > 0.7$, indicating the detection of an earthquake signal by QNO at that threshold. The probability curves predicted by PhaseNO are plotted in red for P-phases and in blue for S-phases. Vertical bars indicate seismic phases determined when the probabilities are larger than a picking threshold of 0.3. QNO and PhaseNO produce consistent detection results for the event with a relatively high SNR. For the event with a lower SNR, PhaseNO did not find picks at many stations that contain earthquake signals classified by QNO. These low quality events will be easily overlooked by PhaseNO+GaMMA when building earthquake catalogs. In contrast, QNO successfully detected these microseismic events and directly determined their locations and origin time from the input waveforms.

Supplementary Note 3: Multi-event superposition test for QNO

Supplementary Figs. 32 and 33 illustrate the performance of QNO when two events occur within a single 15 s time window under varying amplitude ratios and inter-event delay times.

To generate these examples, we manually superposed waveforms from two events in the NCEDC test dataset while controlling their relative amplitude ratio $r \in [0, 1]$ and inter-event delay time Δ. The delay time is defined as the difference between the earliest P-wave arrivals across all stations for the two events. The resulting superposed signal can be expressed as

$$s(t) = A_1 w_1(t) + r A_1 w_2(t - \Delta), \tag{5}$$

where A_1 is the amplitude of the first event, $w_1(t)$ and $w_2(t)$ are normalized waveforms for Event 1 and Event 2, and r and Δ control the relative strength and timing of Event 2. We find that QNO still produces high signal probabilities at nearly all stations and returns a single location estimate for the input window, even when some stations lack clear phase picks for both events.

We evaluated QNO's predictions by comparing them with the catalog locations and origin times of both events to determine which event was actually located and how the presence of one event affected the location of the other. The results show that QNO consistently located the earlier-occurring event (Event 1) when both were present. Increasing the amplitude ratio r made Event 2 more prominent and reduced its origin-time error, but Event 1's location errors remained relatively stable. Longitude errors for both events were less sensitive to r, likely due to the limited azimuthal coverage of the stations. Varying the delay time Δ showed that larger delays generally increased Event 2's errors, while Event 1 remained largely unaffected. These trends were not strictly linear because of waveform complexity, uneven station distribution, and the fact that some stations did not record both events.

Supplementary Note 4: Comparison of data-splitting strategies for the NCEDC dataset

We considered two strategies for splitting the dataset into training, validation, and test datasets. In the primary setting, we adopted a temporal split: events that occurred before 2016 were used for training, those from 2016 for validation, and those from 2017 to 2021 for testing (Figure 2). This design reflects a practical deployment scenario in seismology, where models are typically trained on historical earthquakes and then applied to detect or locate future events. In this setting, the training and test data may exhibit distributional differences due to changes in station coverage, noise characteristics, or catalog completeness. Rather than assuming stationarity, this setup intentionally introduces temporal distribution shift to evaluate the model's ability to generalize under more realistic and challenging conditions. Unless otherwise specified, all evaluations are conducted using the model trained on events prior to 2016.

To complement this, and to isolate the model's capacity under controlled conditions, we also performed an alternative experiment using randomly shuffled indices. Specifically, we permuted all 10,393 events with a fixed seed and assigned 7,972 events to the training set, 1,402 to the validation set, and 1,019 to the test set, ensuring no overlap (Supplementary Fig. 4). This random split ensures that all subsets are sampled from the same overall distribution, minimizing the effect of distribution shift and providing a more standard evaluation setting.

We trained and evaluated QNO under both splitting strategies. As expected, the model performed better with the random split than with the temporal split, as shown by the comparison between Figure 4 and Supplementary Fig. 5, consistent with a reduced generalization gap. Nevertheless, the performance under the temporally split setting remains competitive, demonstrating the model's robustness even when evaluated on future events with potential distributional changes.

References

[1] Nikola Kovachki, Zongyi Li, Burigede Liu, Kamyar Azizzadenesheli, Kaushik Bhattacharya, Andrew Stuart, and Anima Anandkumar. Neural operator: Learning maps between function spaces with applications to PDEs. *Journal of Machine Learning Research*, 24(89):1–97, 2023.

[2] Zongyi Li, Nikola Kovachki, Kamyar Azizzadenesheli, Burigede Liu, Kaushik Bhattacharya, Andrew Stuart, and Anima Anandkumar. Fourier neural operator for parametric partial differential equations. *arXiv preprint arXiv:2010.08895*, 2020.

[3] Zongyi Li, Nikola Kovachki, Kamyar Azizzadenesheli, Burigede Liu, Kaushik Bhattacharya, Andrew Stuart, and Anima Anandkumar. Neural operator: Graph kernel network for partial differential equations. *arXiv preprint arXiv:2003.03485*, 2020.

[4] Dan Hendrycks and Kevin Gimpel. Gaussian Error Linear Units (GELUs). *arXiv preprint arXiv:1606.08415*, 2016.

[5] Justin Gilmer, Samuel S Schoenholz, Patrick F Riley, Oriol Vinyals, and George E Dahl. Neural message passing for quantum chemistry. In *International Conference on Machine Learning*, pages 1263–1272, 2017.

[6] Hongyu Sun, Zachary E Ross, Weiqiang Zhu, and Kamyar Azizzadenesheli. Phase neural operator for multi-station picking of seismic arrivals. *Geophysical Research Letters*, 50(24):e2023GL106434, 2023.

[7] S. Mostafa Mousavi, Yixiao Sheng, Weiqiang Zhu, and Gregory C. Beroza. STanford EArthquake Dataset (STEAD): A global data set of seismic signals for AI. *IEEE Access*, 7:179464–179476, 2019.

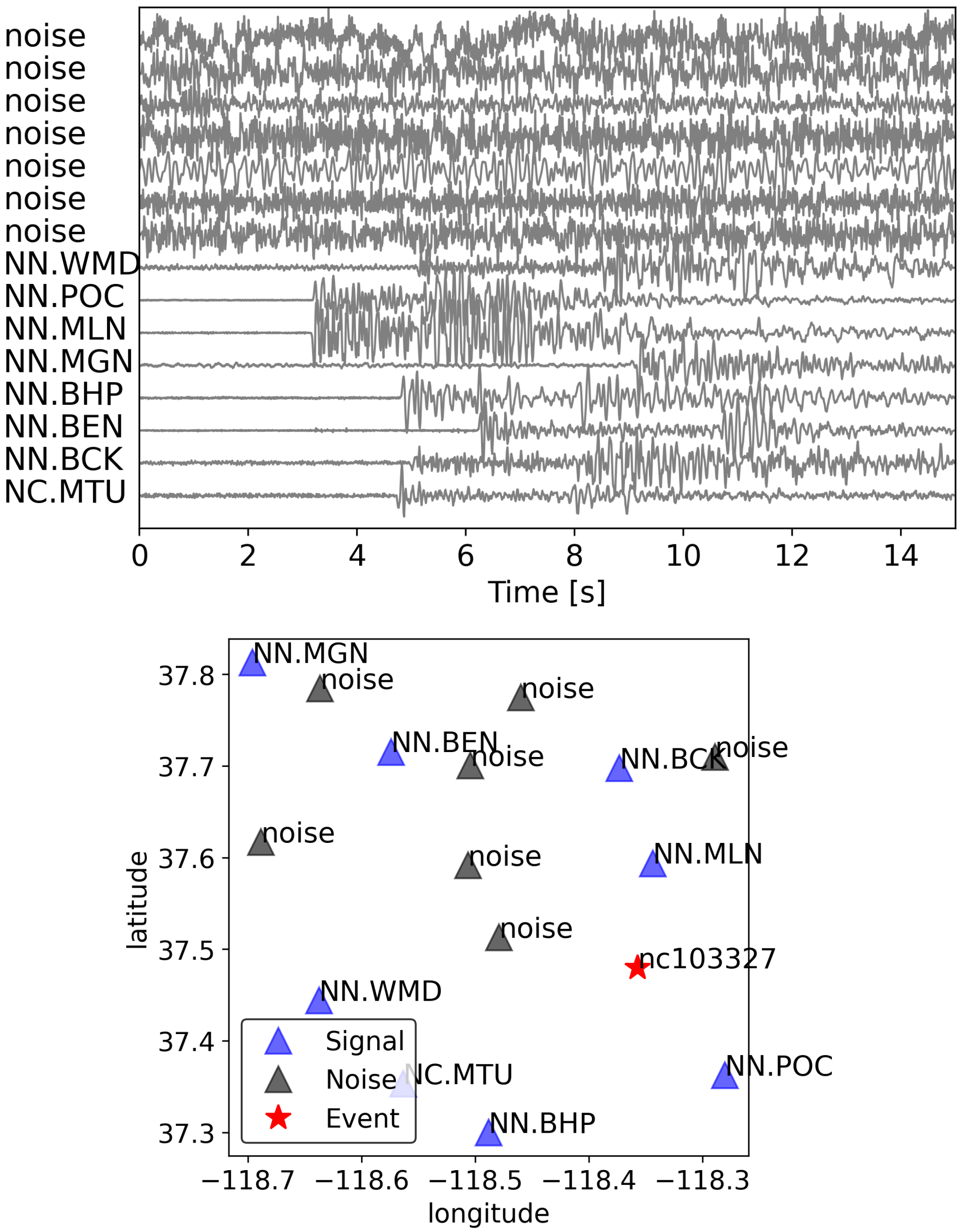

Supplementary Fig. 1: **Example of a training sample (event nc103327).** Seven stations are randomly placed within the seismic network, and real noise from the STEAD dataset [7] is assigned to each station.

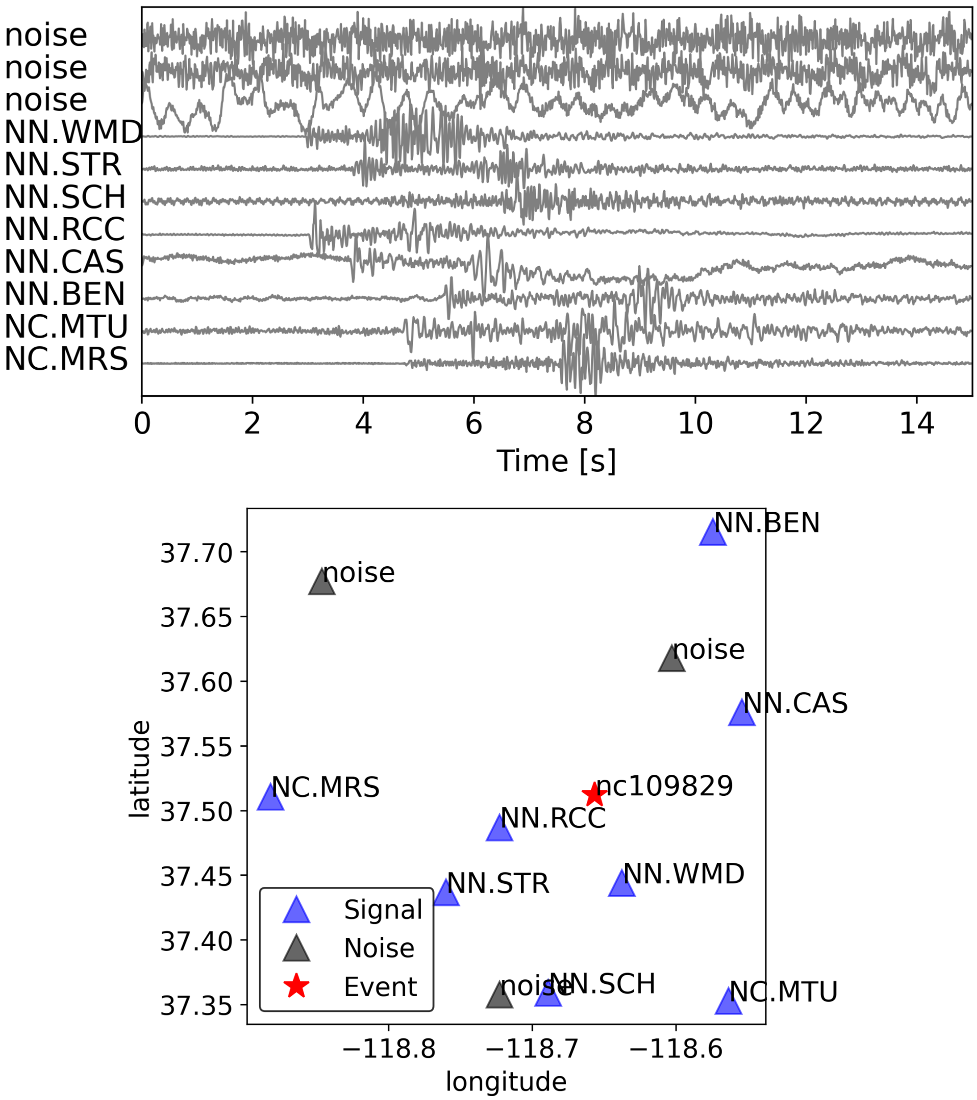

Supplementary Fig. 2: **Example of a training sample (event nc109829).** Three stations are randomly placed within the seismic network, and real noise from the STEAD dataset [7] is assigned to each station.

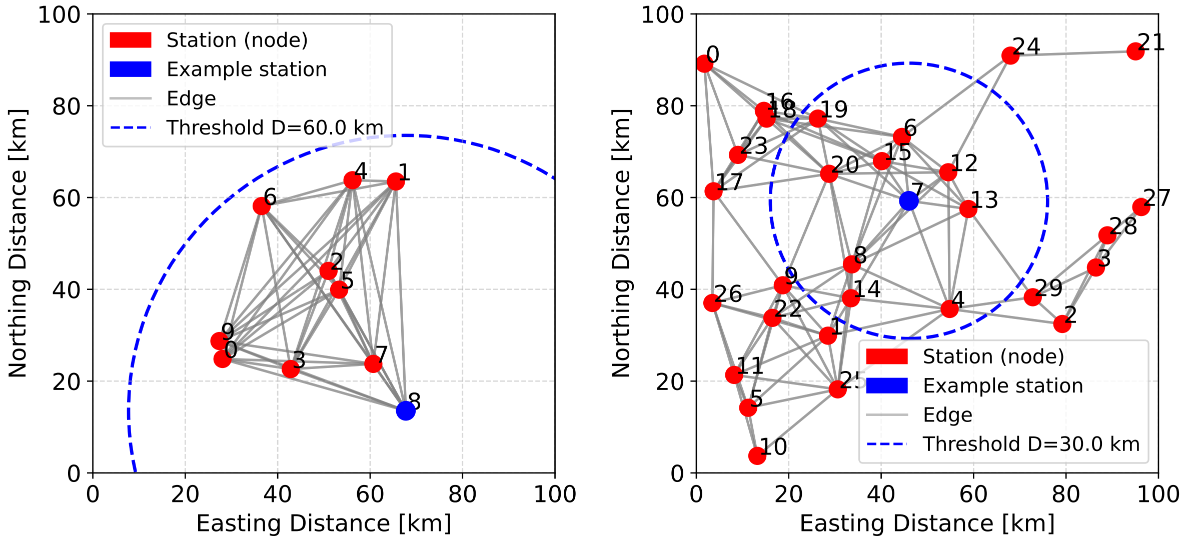

Supplementary Fig. 3: **Illustration of graph construction in the GNO layer based on a geographic distance threshold D.** Each node represents a seismic station, and edges are established between pairs of stations whose geographic distance is less than or equal to D. Self-loops (edges from a node to itself) are included. The computational cost is largely affected by the number of edges in the graph (Figure 9), which is controlled by the relative distance between the distance among stations and the distance threshold D. For the same graph, increasing D increases the number of edges, thereby enlarging the neighborhood of each node and raising the computational cost due to additional node-to-node communications. For example, the 10 stations in the left panel become fully connected (i.e., form a complete graph) when D is set to 60 km. In contrast, when a large number of stations are present, as in the right panel, a relatively small D can be chosen to reduce computational cost while still preserving effective communication among stations, as evidenced by generalization when training with $D = 40\,\text{km}$ and testing with $D = 20\,\text{km}$ and $D = 60\,\text{km}$ (Supplementary Fig. 30).

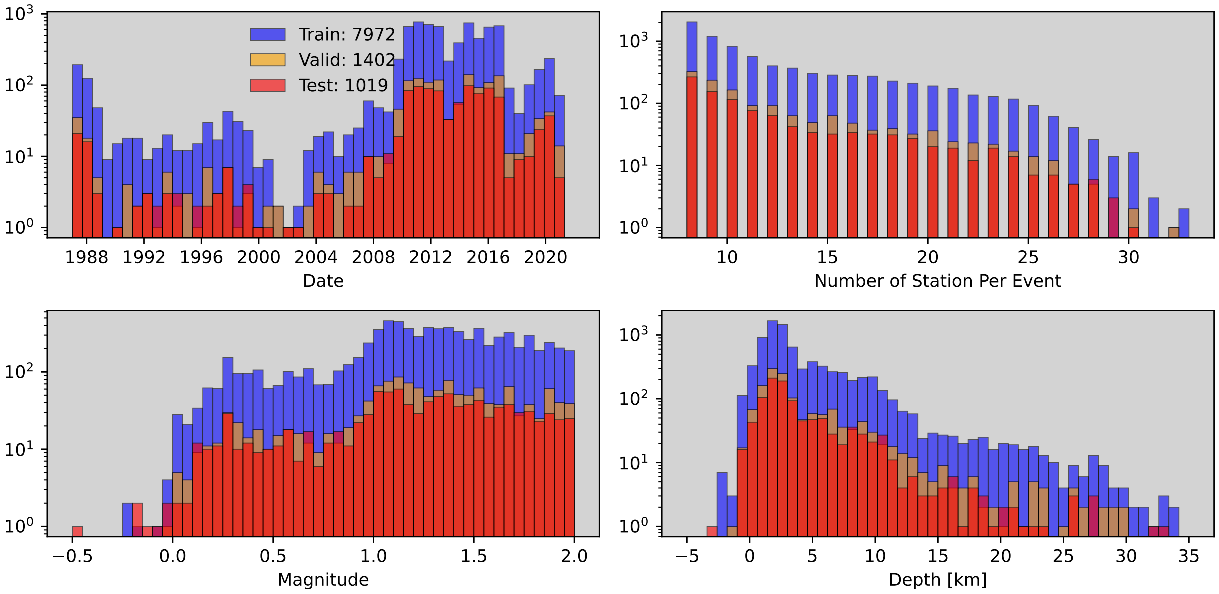

Supplementary Fig. 4: **NCEDC seismic dataset split using randomly shuffled indices.** We adopt a random shuffling strategy to construct the training, validation, and test datasets, ensuring that all three subsets follow the same distribution. Specifically, we randomly permute the indices of all available events and select the number of events in each subset to match those shown in Figure 2 of the main text. Supplementary Fig. 5 shows the performance of QNO on this dataset.

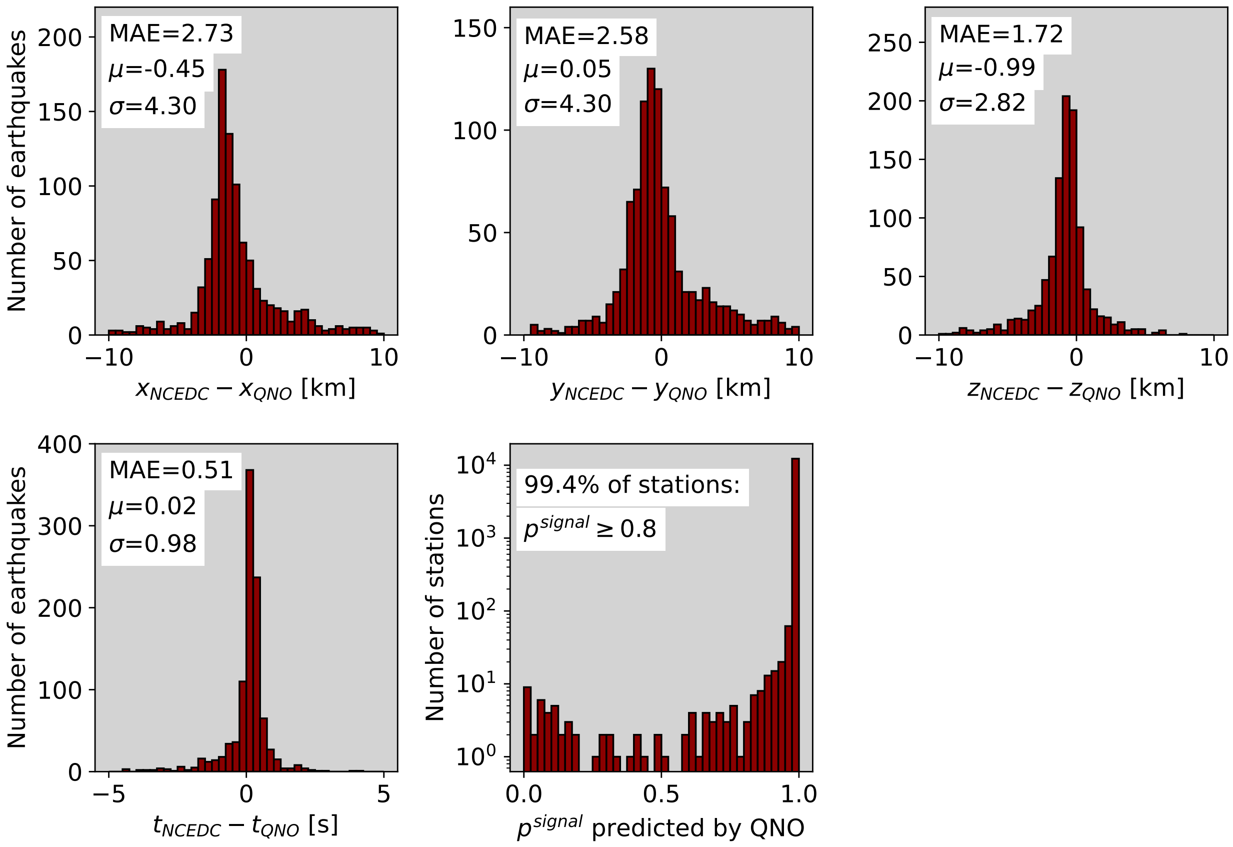

Supplementary Fig. 5: **Performance of QNO on the NCEDC test dataset split using uniformly random indices.** Each 15-s input seismogram contains 1500 sampling points, consistent between the training and test sets. Supplementary Fig. 4 shows the property of the test dataset. The QNO model performed better with the random split than with the temporal split (Figure 4), owing to a reduced generalization gap between the training and test datasets.

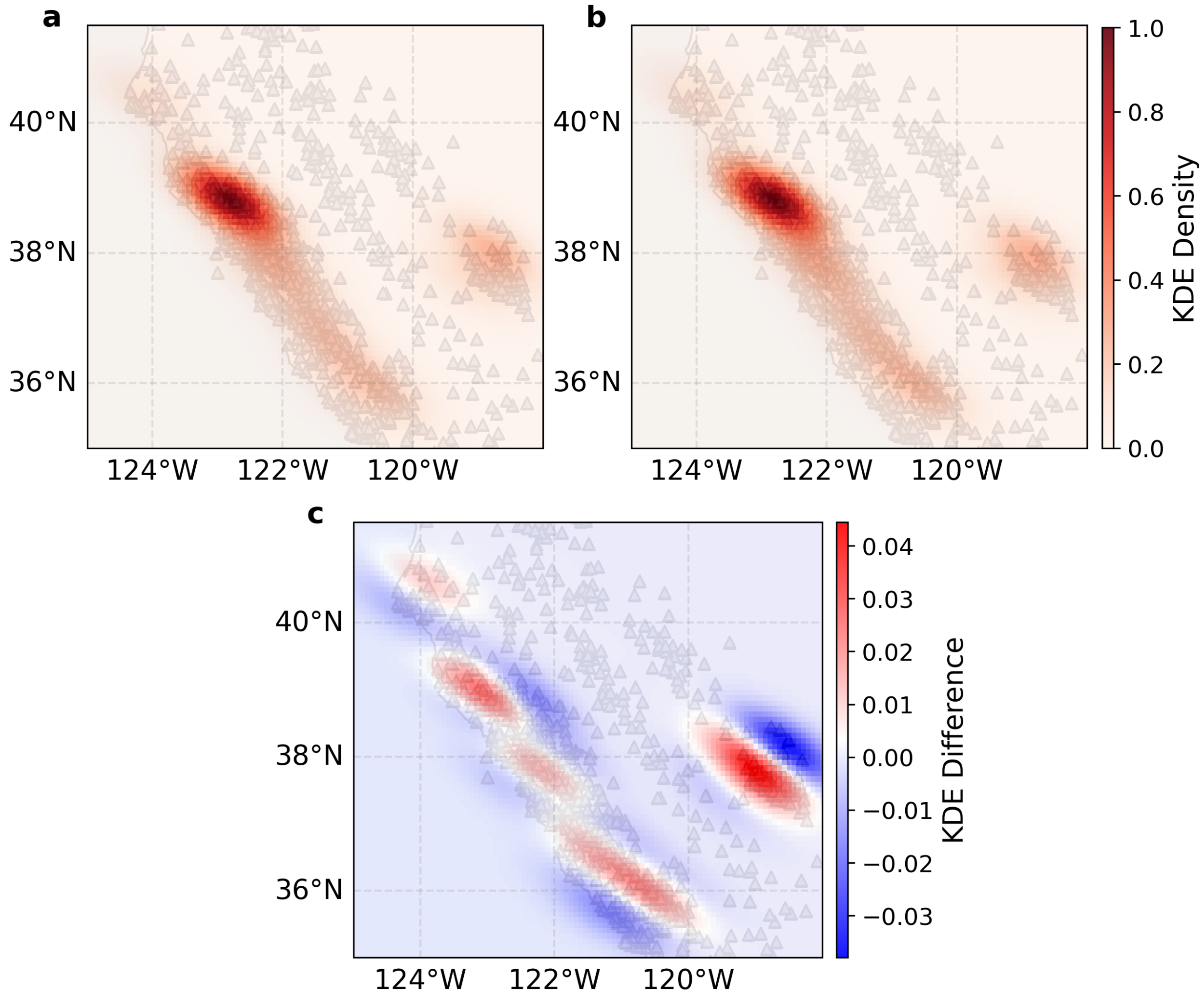

Supplementary Fig. 6: **Comparison of kernel density estimates (KDEs) between QNO predictions and the NCEDC catalog on the test dataset.** **a** NCEDC catalog locations. **b** QNO predicted locations. **c** KDE difference (QNO - NCEDC). This comparison highlights spatial patterns and potential errors in the predicted epicenters.

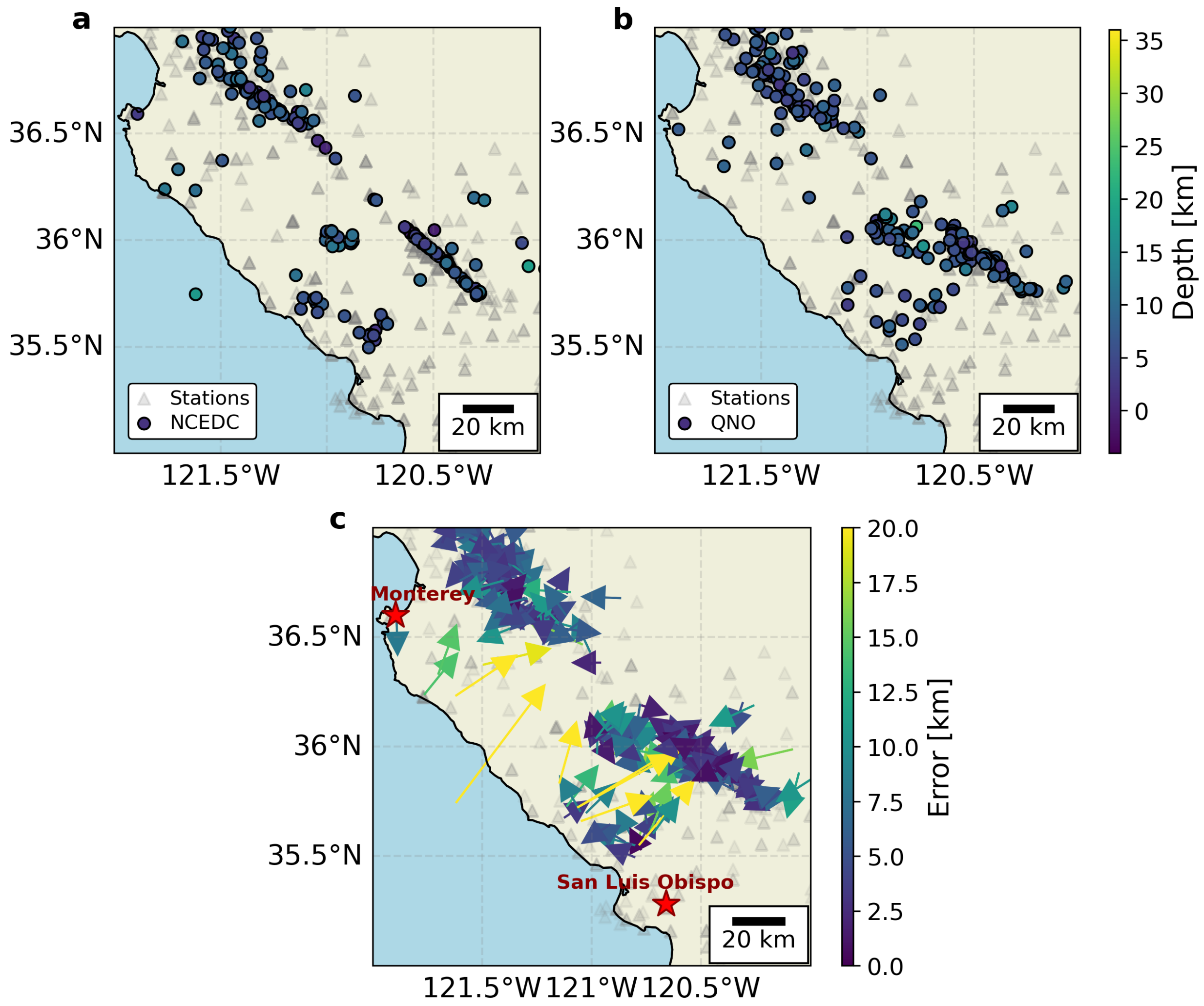

Supplementary Fig. 7: **Zoom-in view of earthquake locations in a selected region of Northern California.** This figure provides a detailed view of the area bounded by San Luis Obispo and Monterey, corresponding to the region highlighted in Figure 3. Both the NCEDC catalog locations and QNO predictions are shown, allowing closer inspection of spatial patterns and local variations in location accuracy.

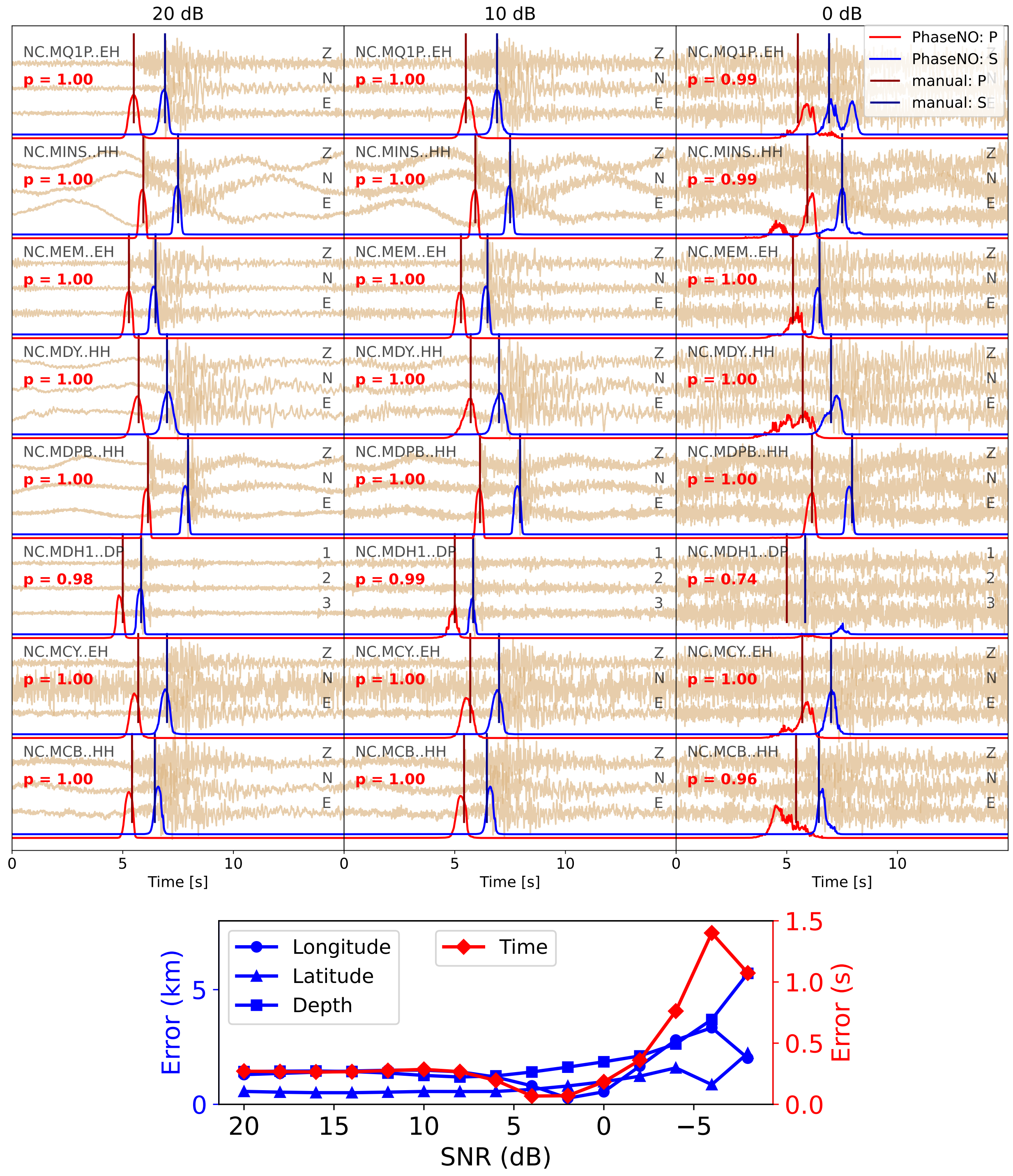

Supplementary Fig. 8: **Detection and location performance for event nc73377575 under varying noise levels.** We add real noise waveforms to event nc73377575 while controlling the SNR from 20 dB to -8 dB. The upper panel compares the probabilities predicted by QNO and PhaseNO at SNR levels of 20 dB, 10 dB, and 0 dB. The probabilities (p) predicted by QNO for each input station are displayed in red beneath the corresponding station names. The lower panel shows the location error of QNO across different noise levels. Overall, performance degrades as SNR decreases. However, QNO successfully detects the event at most stations even at an SNR of 0 dB and consistently estimates its location and origin time. In contrast, PhaseNO fails to pick phases of the event when the SNR drops to 0 dB.

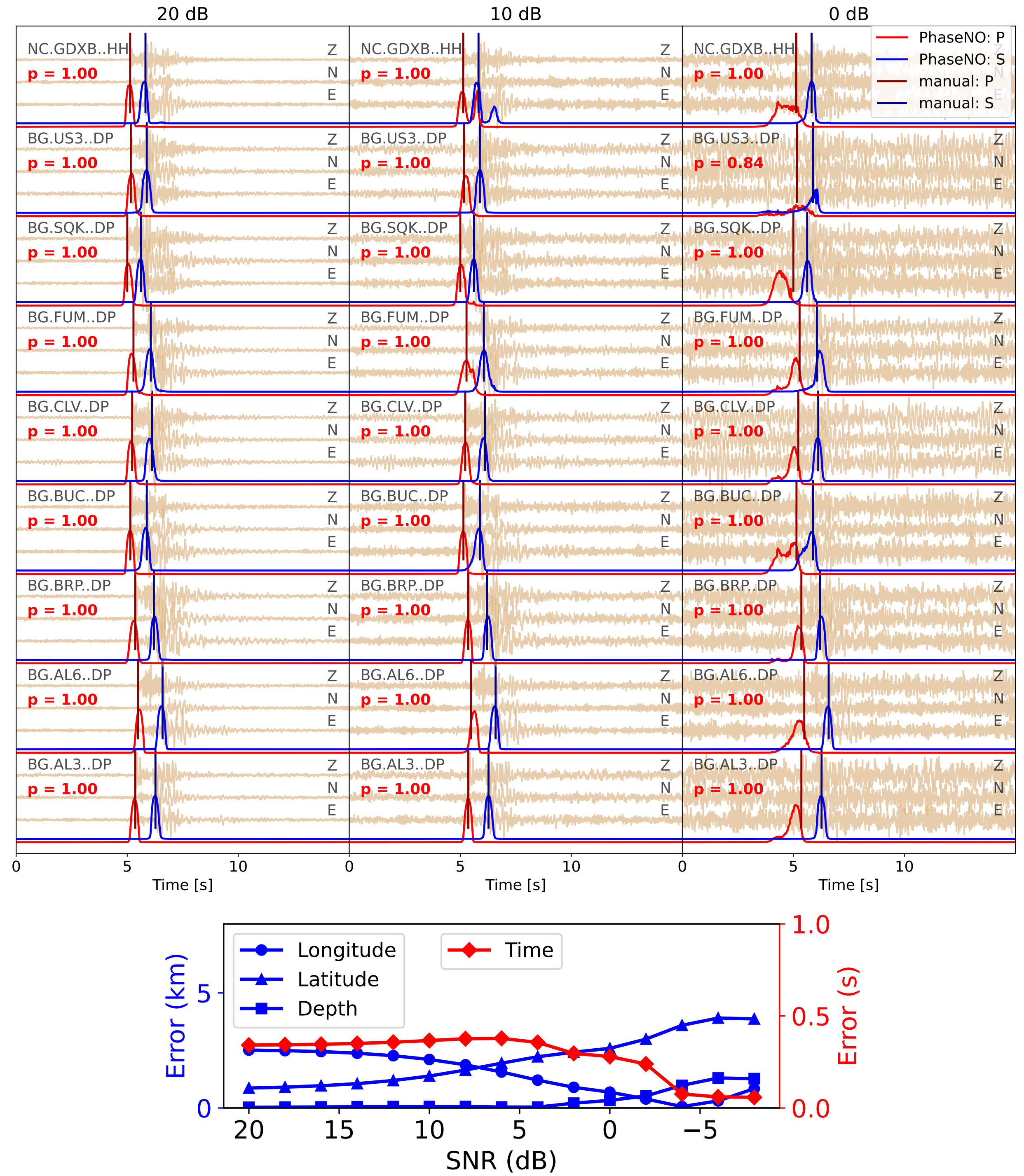

Supplementary Fig. 9: **Detection and location performance for event nc73381225 under varying noise levels.** The annotations on this figure are the same as those in Supplementary Fig. 8.

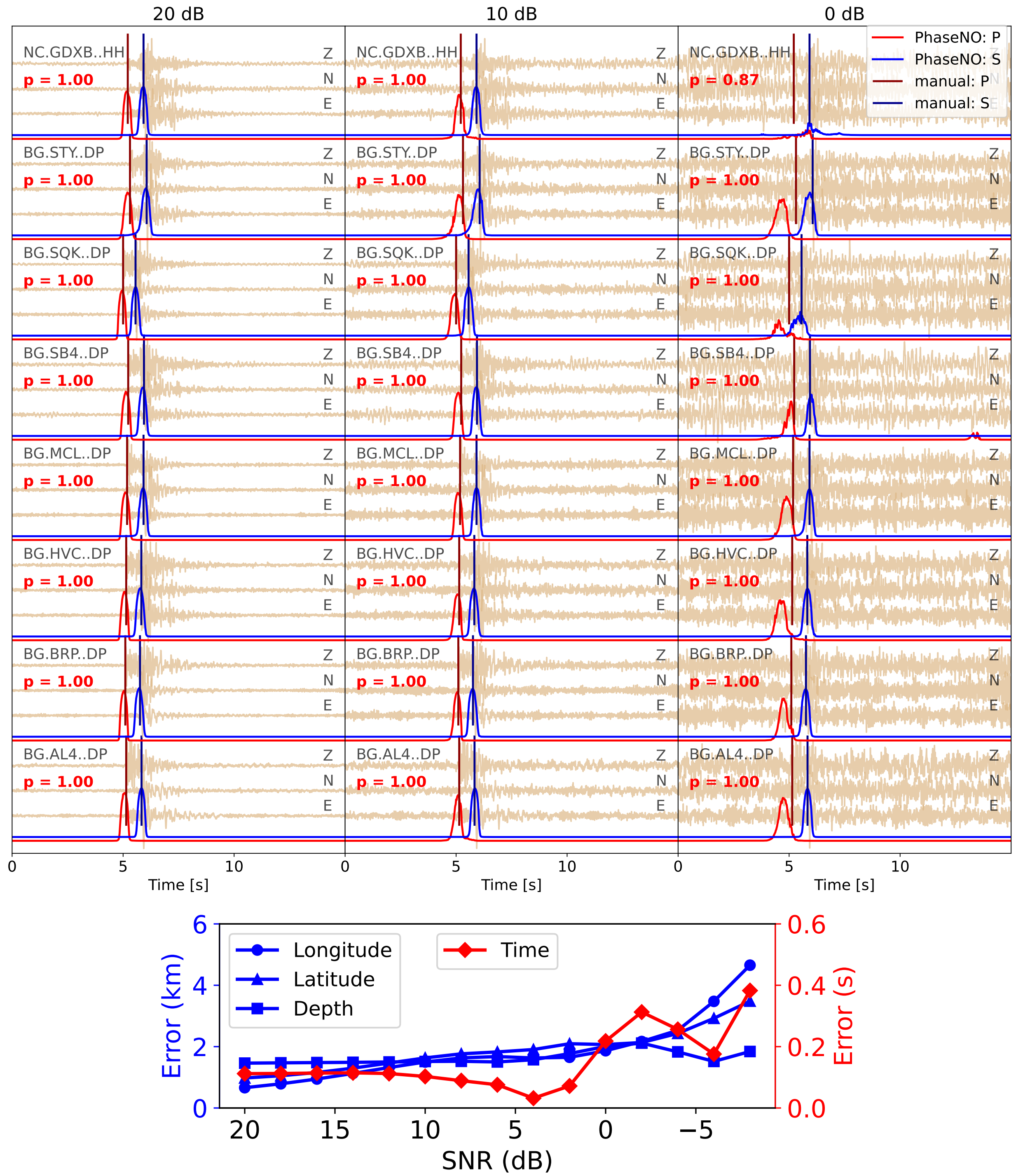

Supplementary Fig. 10: **Detection and location performance for event nc73381495 under varying noise levels.** The annotations on this figure are the same as those in Supplementary Fig. 8.

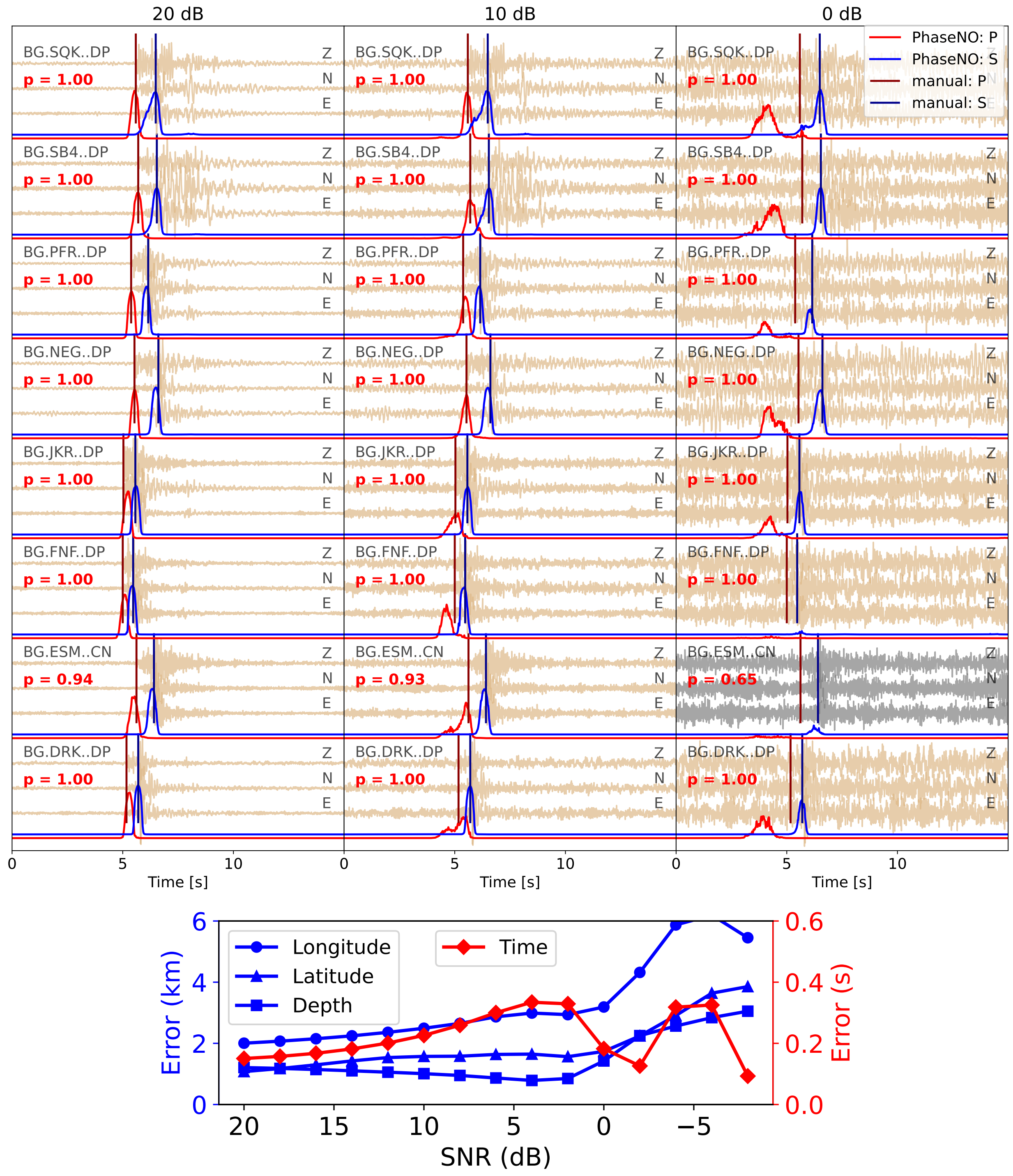

Supplementary Fig. 11: **Detection and location performance for event nc73384250 under varying noise levels.** The annotations on this figure are the same as those in Supplementary Fig. 8.

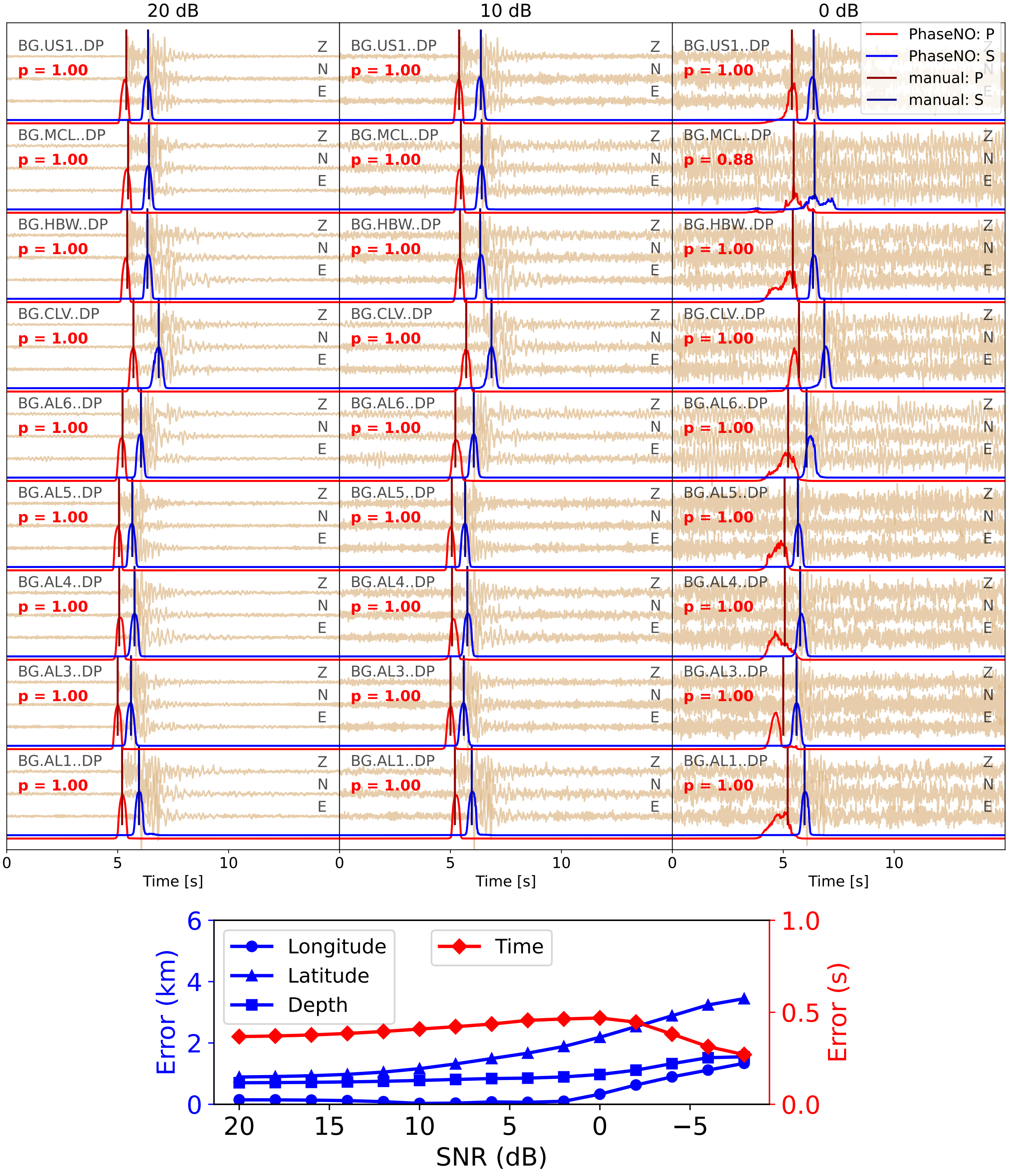

Supplementary Fig. 12: **Detection and location performance for event nc73385040 under varying noise levels.** The annotations on this figure are the same as those in Supplementary Fig. 8.

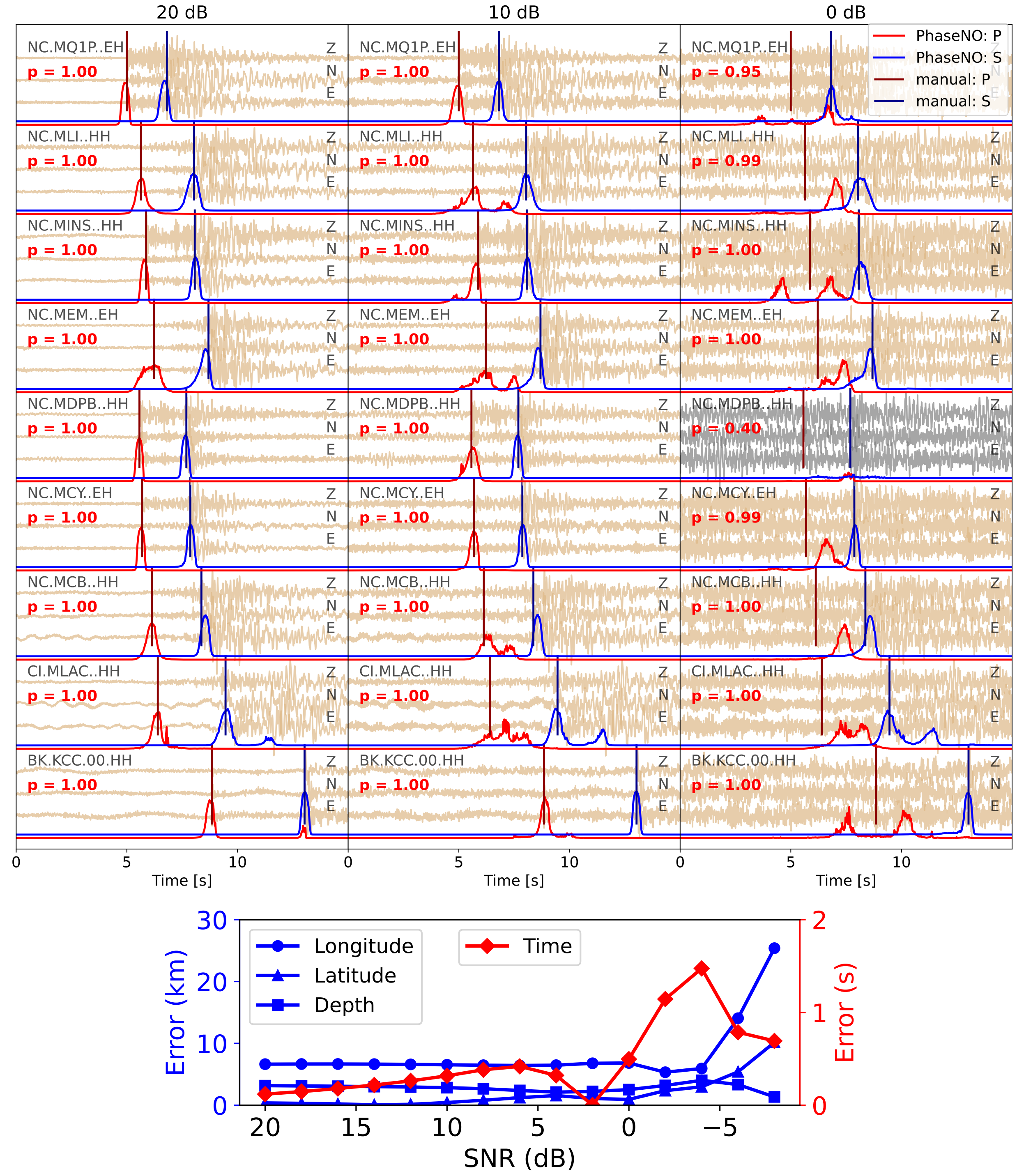

Supplementary Fig. 13: **Detection and location performance for event nc73393926 under varying noise levels.** The annotations on this figure are the same as those in Supplementary Fig. 8.

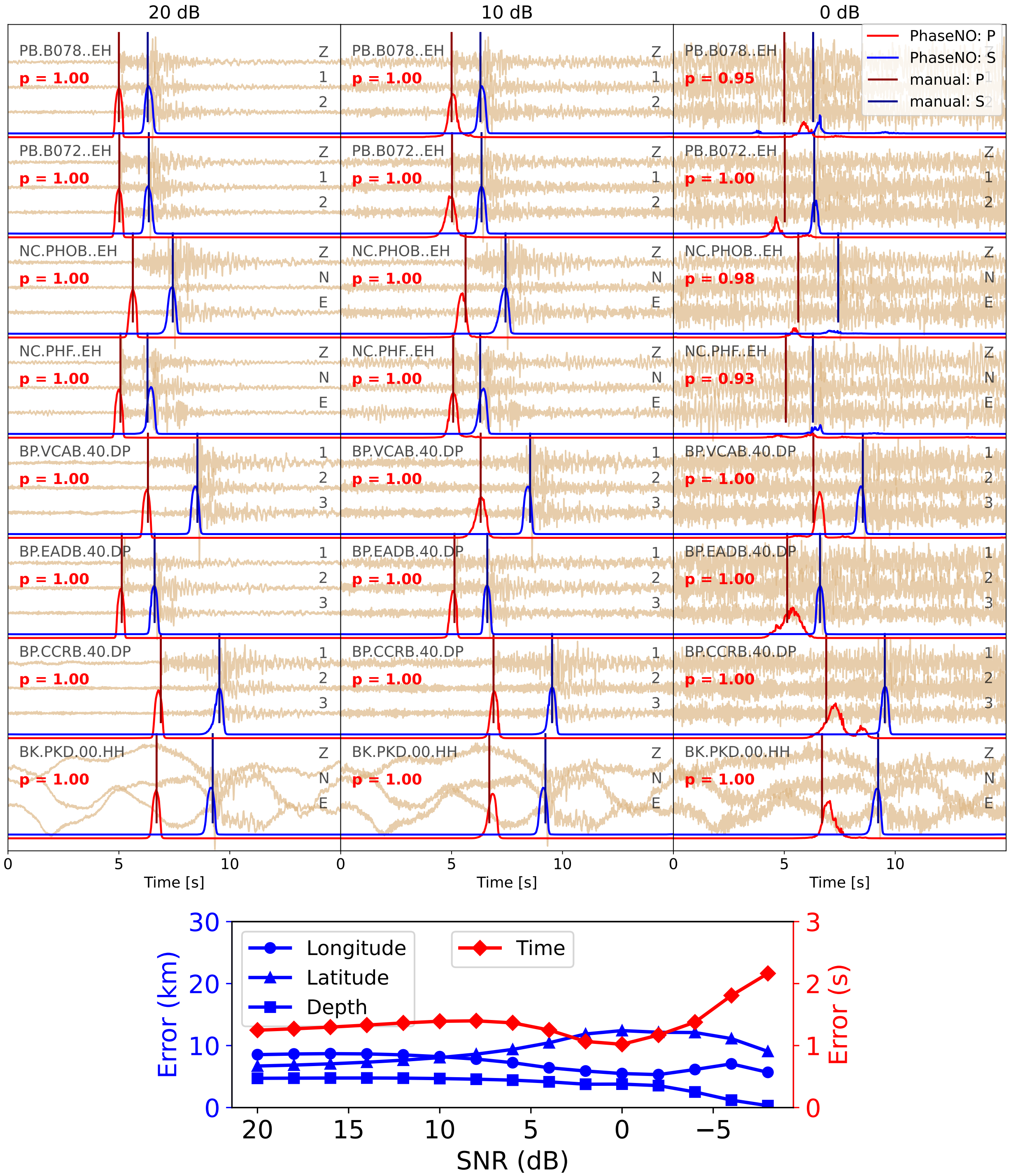

Supplementary Fig. 14: **Detection and location performance for event nc73396946 under varying noise levels.** The annotations on this figure are the same as those in Supplementary Fig. 8.

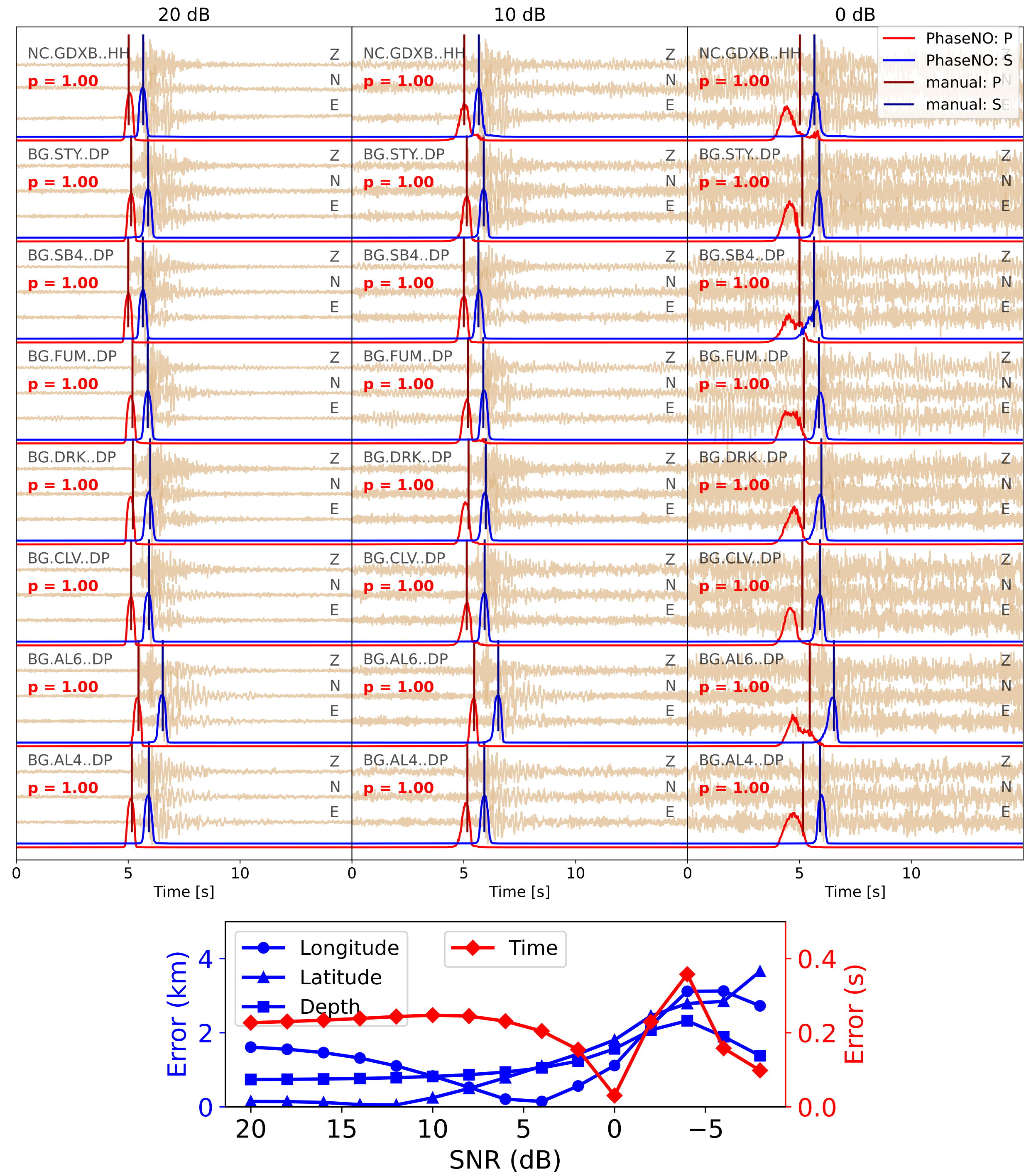

Supplementary Fig. 15: **Detection and location performance for event nc73397346 under varying noise levels.** The annotations on this figure are the same as those in Supplementary Fig. 8.

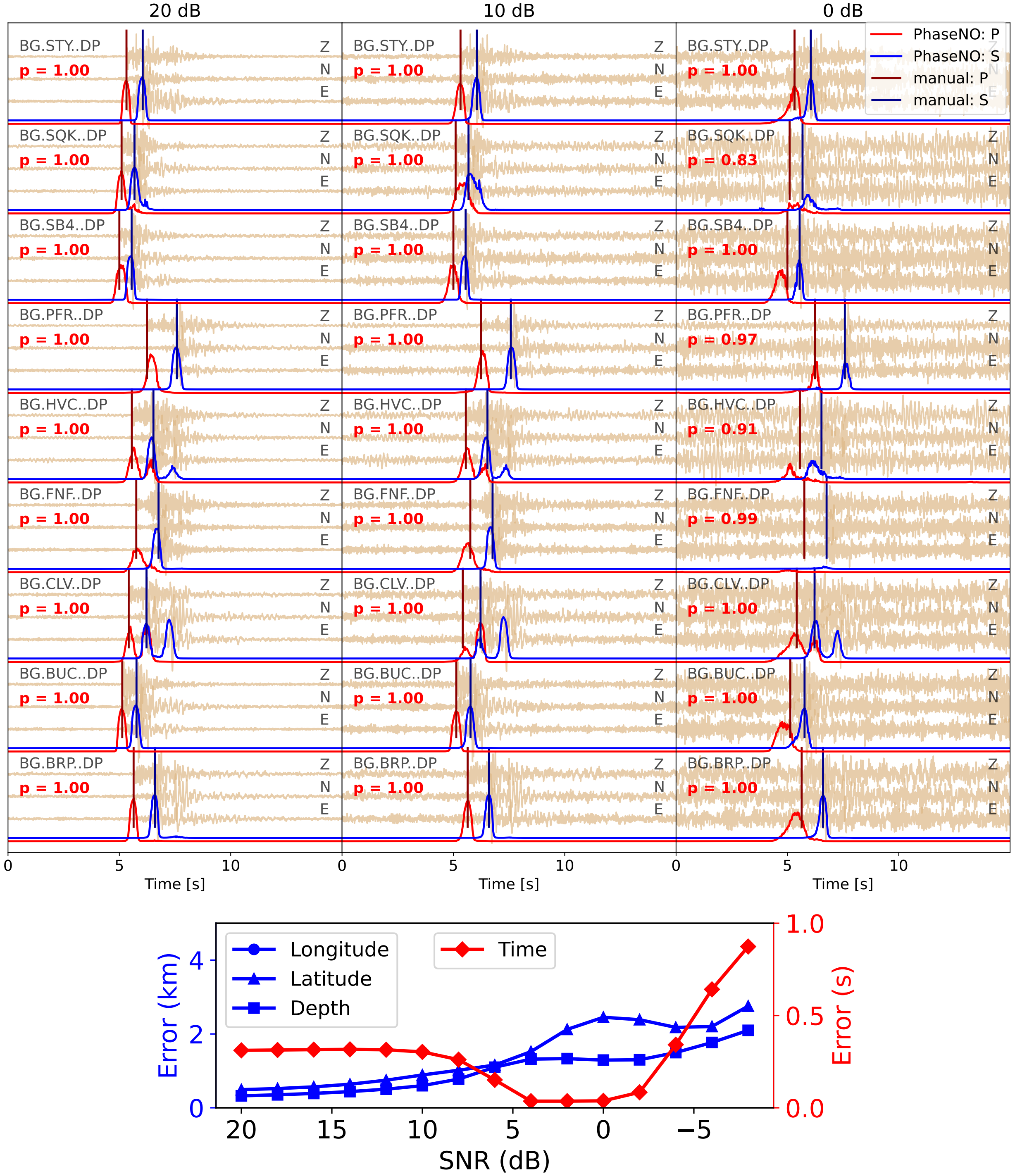

Supplementary Fig. 16: **Detection and location performance for event nc73404441 under varying noise levels.** The annotations on this figure are the same as those in Supplementary Fig. 8.

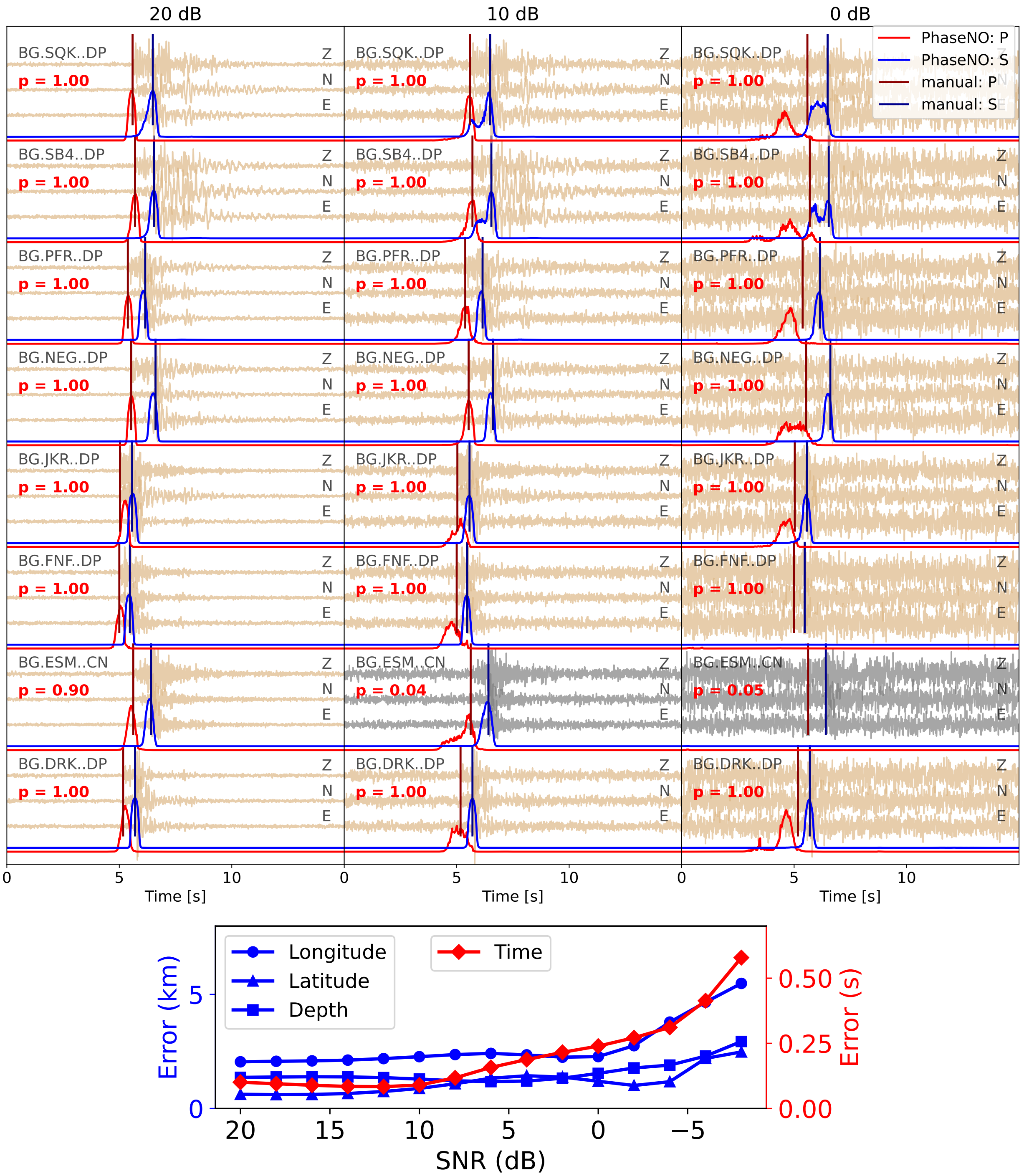

Supplementary Fig. 17: **Detection and location performance for event nc73384250 under varying Gaussian noise levels.** Unlike Figure 5 in the main text, where real-noise waveforms are superimposed on the same event, here we add Gaussian noise into the waveforms. This results in a clearer, approximately monotonic increase in error as the SNR decreases.

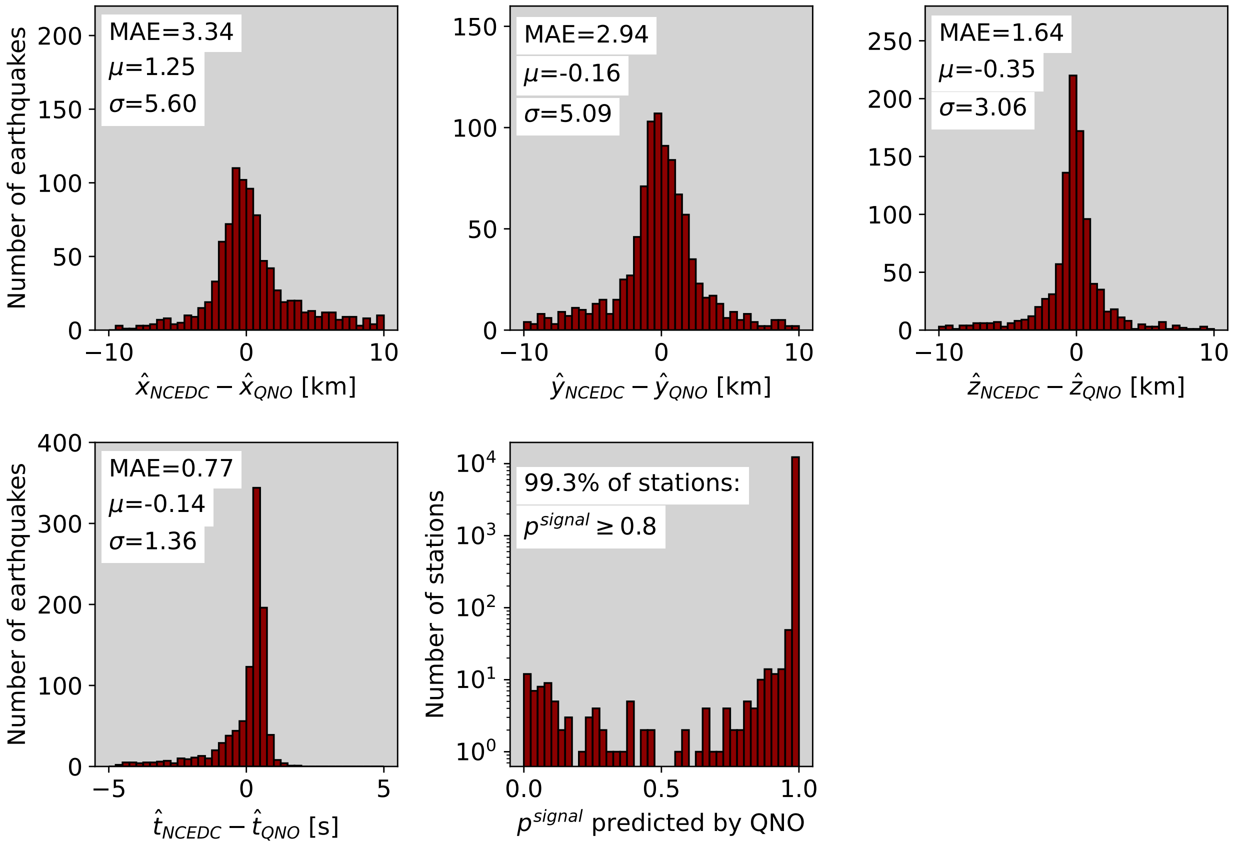

Supplementary Fig. 18: **Generalization to higher temporal resolution for QNO trained with 1,500 samples per 15 s input on the NCEDC test dataset (see Supplementary Fig. 4).** During training, each 15 s seismogram contained 1,500 samples, whereas at test time the seismograms were upsampled to 3,000 samples (i.e., twice the sampling rate) to evaluate the model's ability to generalize to higher temporal resolution.

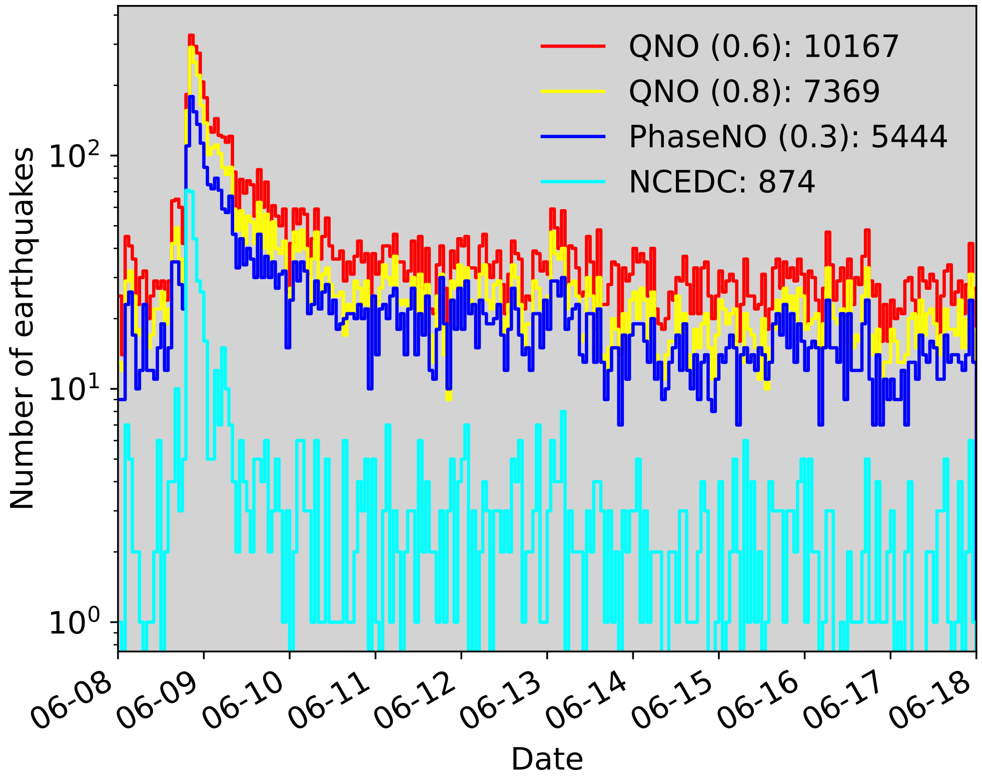

Supplementary Fig. 19: **Hourly earthquake occurrence rates from four catalogs of the Geysers geothermal dataset during the period from 00:00 on June 8, 2024 to 00:00 on June 18, 2024.** Step-line plots show the number of events per hour for QNO with a detection probability threshold of 0.6 (red), QNO with a higher threshold of 0.8 (yellow), PhaseNO with a threshold of 0.3 (blue), and the NCEDC catalog (cyan). Counts are plotted on a logarithmic y-axis to highlight variations across a wide range of seismic activity levels.

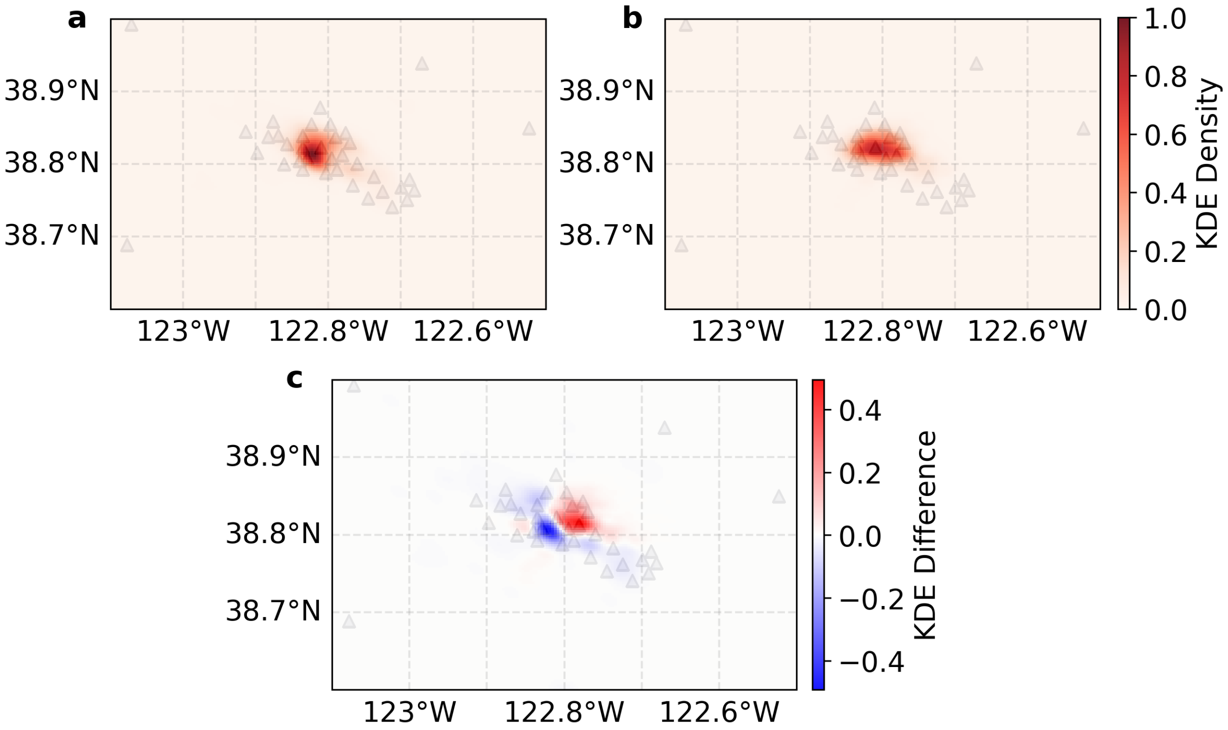

Supplementary Fig. 20: **Comparison of KDEs between QNO predictions and the NCEDC catalog on the Geysers geothermal dataset.** **a** NCEDC catalog. **b** QNO catalog. **c** KDE difference (QNO - NCEDC). Higher KDE densities in the QNO catalog reflect its increased detection rate.

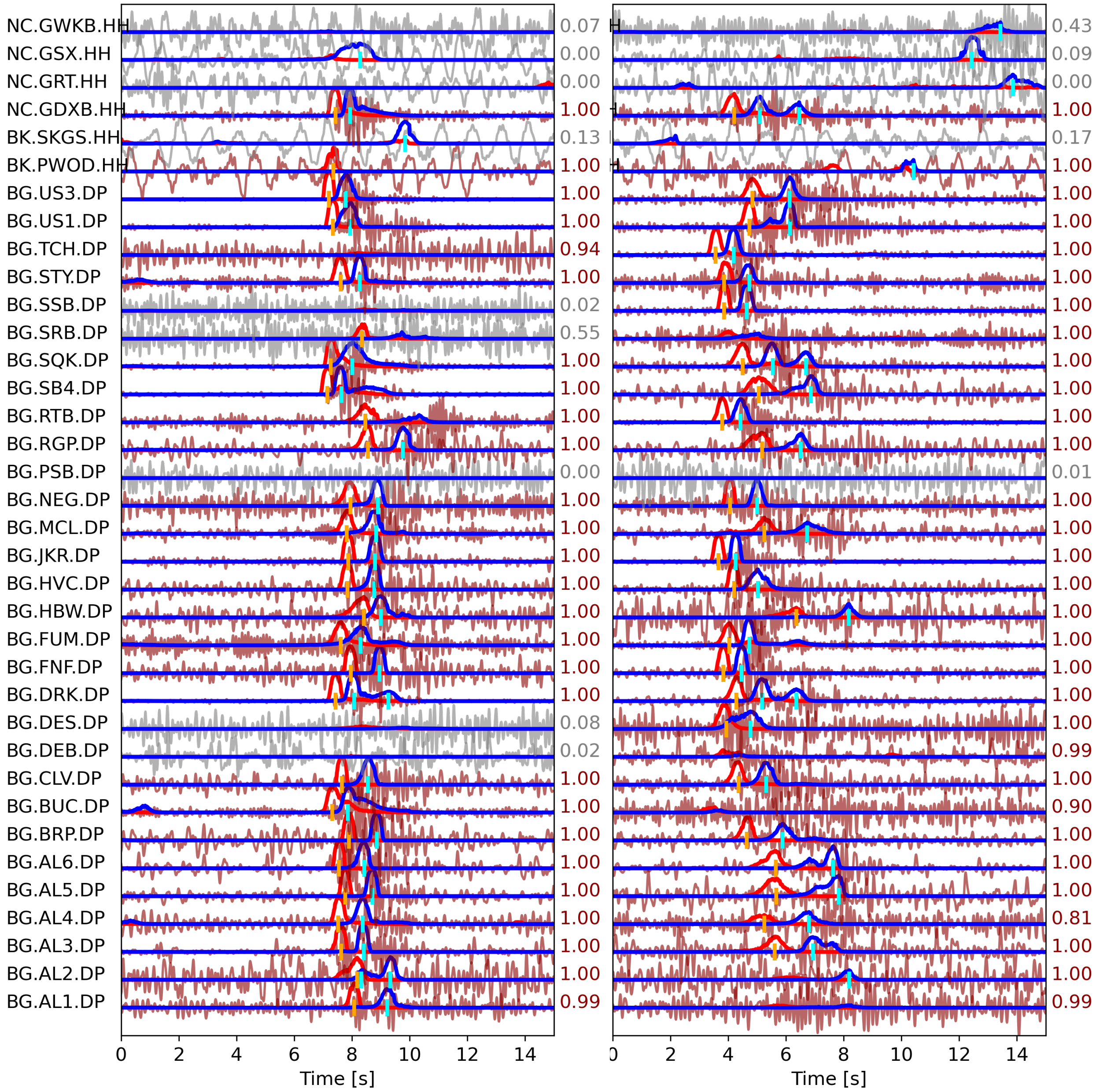

Supplementary Fig. 21: **Comparison of detection performance between QNO and PhaseNO for two microseismic events in the Geysers geothermal field.** Waveforms of all input stations are plotted. The signal probability p_i^{signal} predicted by QNO is shown at the end of each waveform. Waveforms are highlighted in red if $p_i^{\text{signal}} > 0.7$, indicating the detection of an earthquake signal by QNO at that threshold. The probability curves predicted by PhaseNO are plotted in red for P-phases and in blue for S-phases. Vertical bars indicate seismic phases determined when the probabilities are larger than a picking threshold of 0.3. QNO and PhaseNO produce consistent detection results for the event with a relatively high SNR. Conversely, for the event with a lower SNR, QNO identifies signals on more stations than PhaseNO. QNO detected these low-quality events and directly determined their locations from these input waveforms.

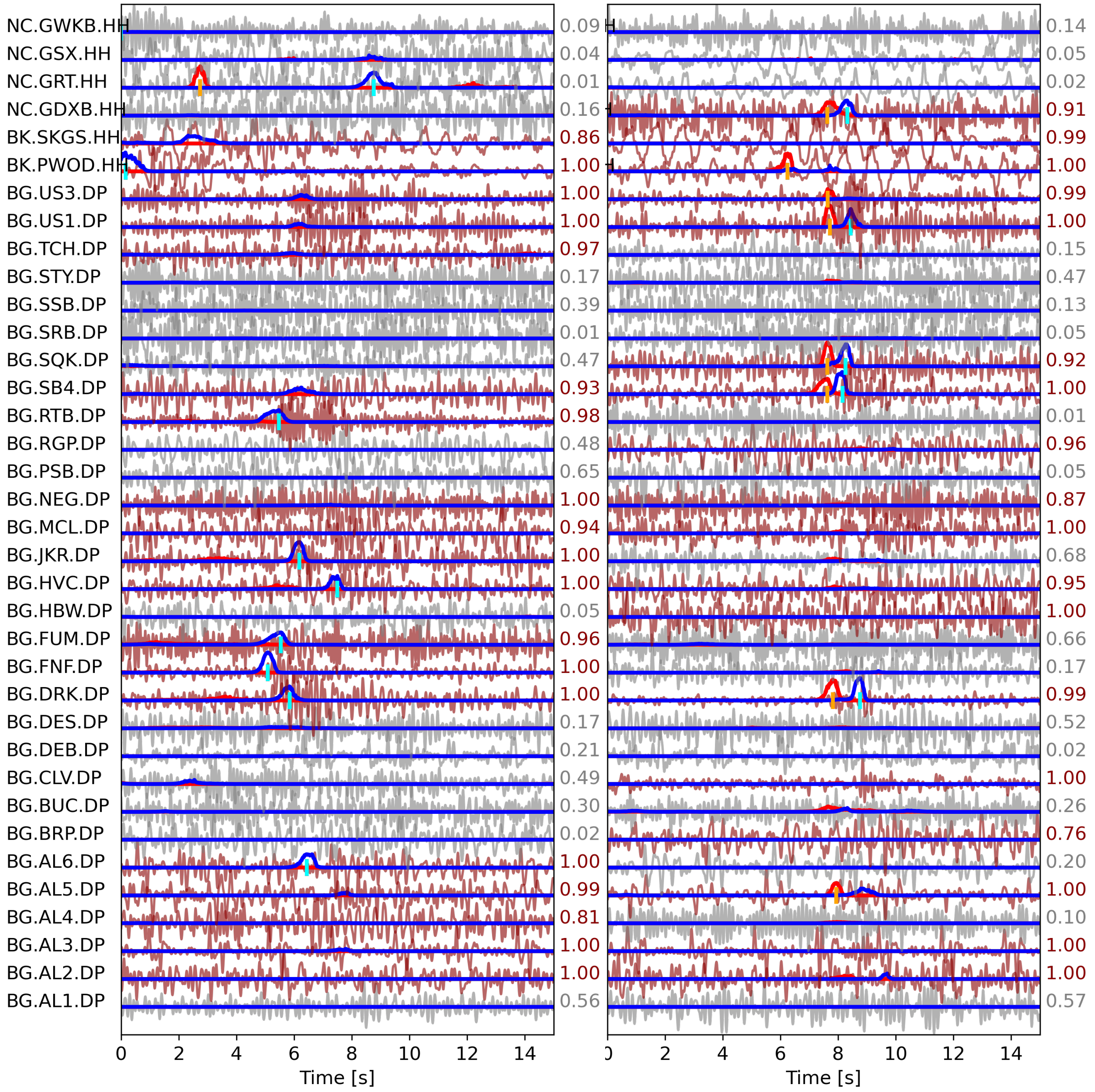

Supplementary Fig. 22: **Comparison of detection performance between QNO and PhaseNO for two microseismic events in the Geysers geothermal field.** The annotations on this figure are the same as those in the Supplementary Fig. 21.

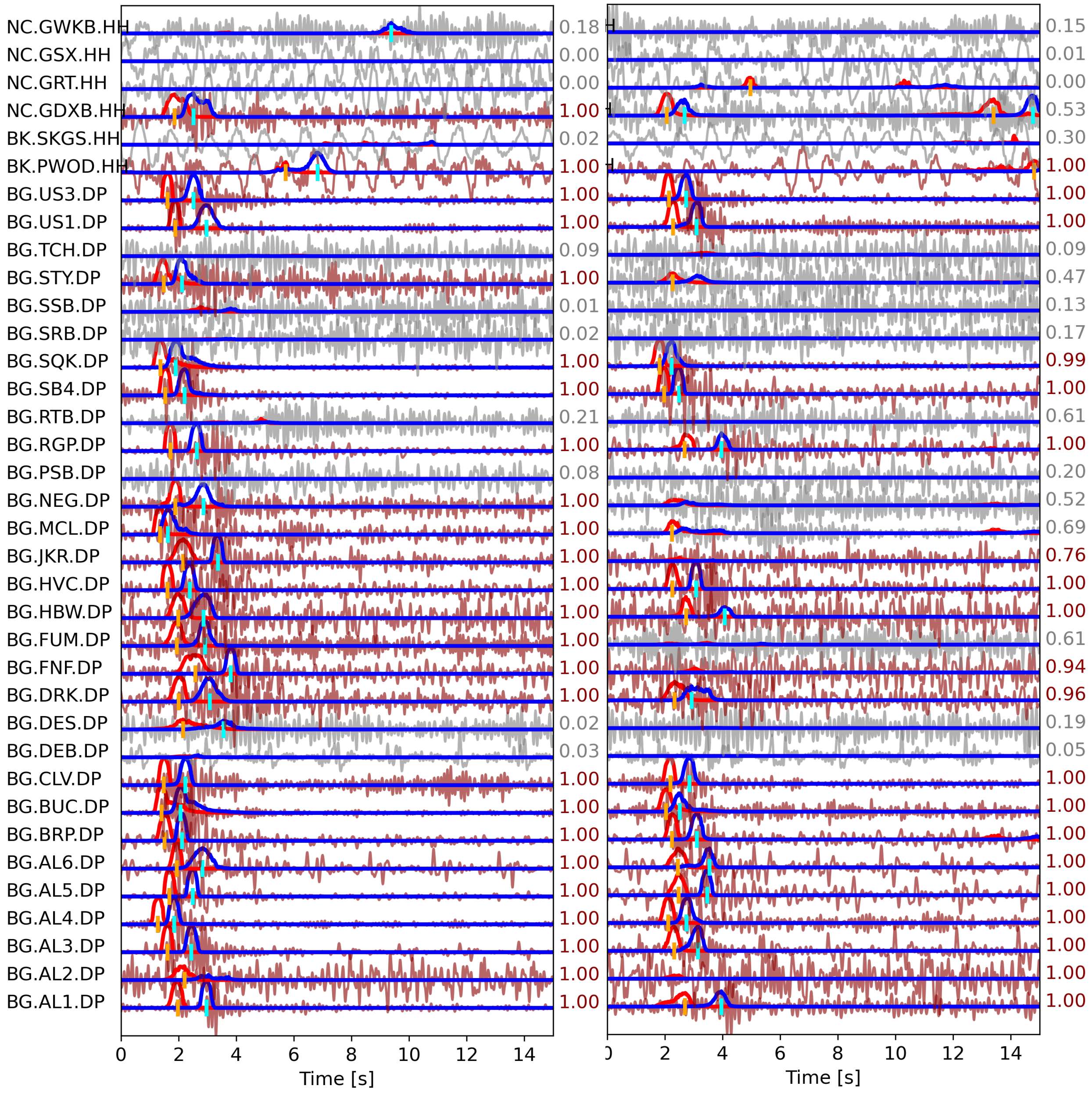

Supplementary Fig. 23: **Comparison of detection performance between QNO and PhaseNO for two microseismic events in the Geysers geothermal field.** The annotations on this figure are the same as those in the Supplementary Fig. 21.

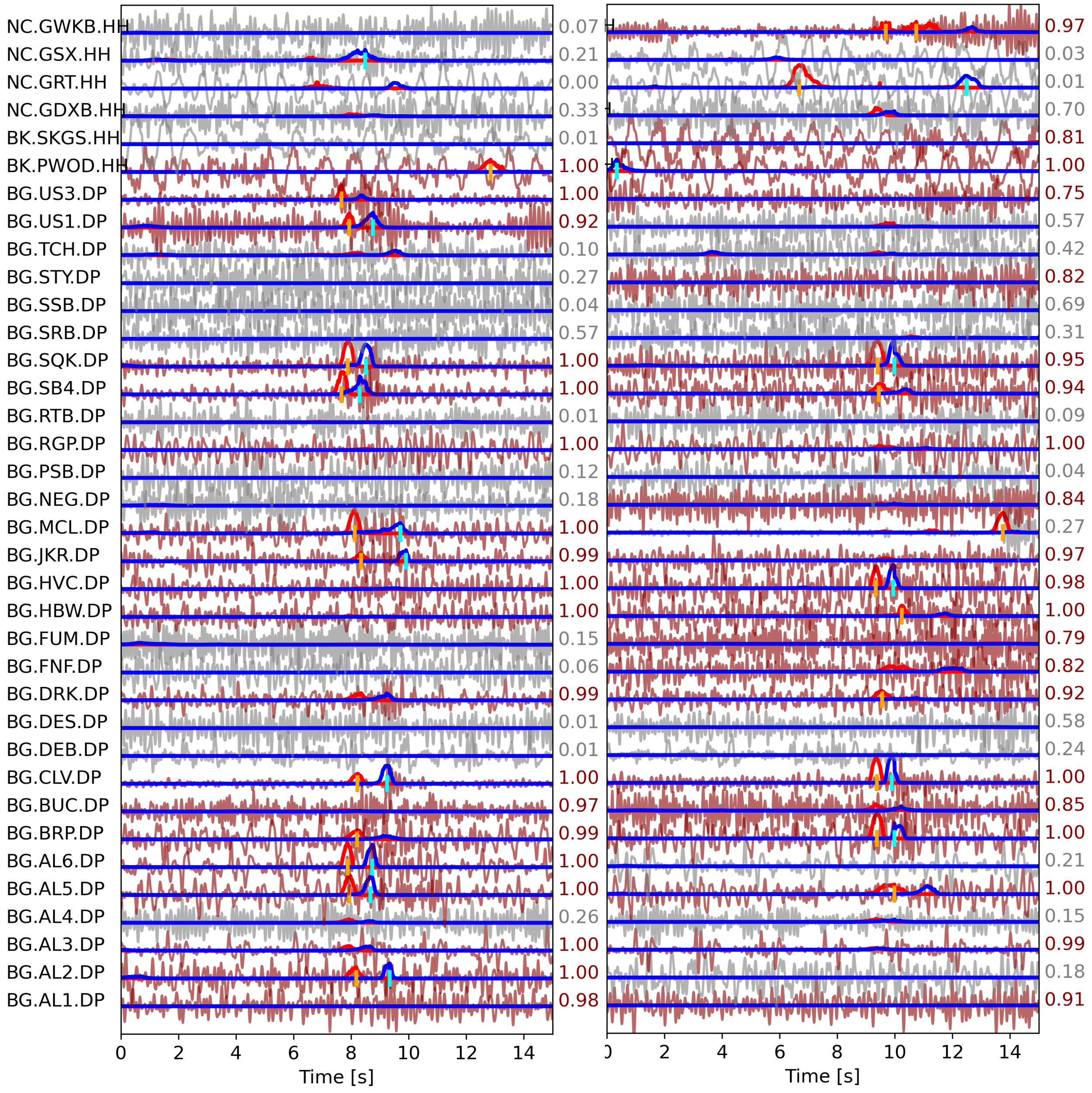

Supplementary Fig. 24: **Comparison of detection performance between QNO and PhaseNO for two microseismic events in the Geysers geothermal field.** The annotations on this figure are the same as those in the Supplementary Fig. 21.

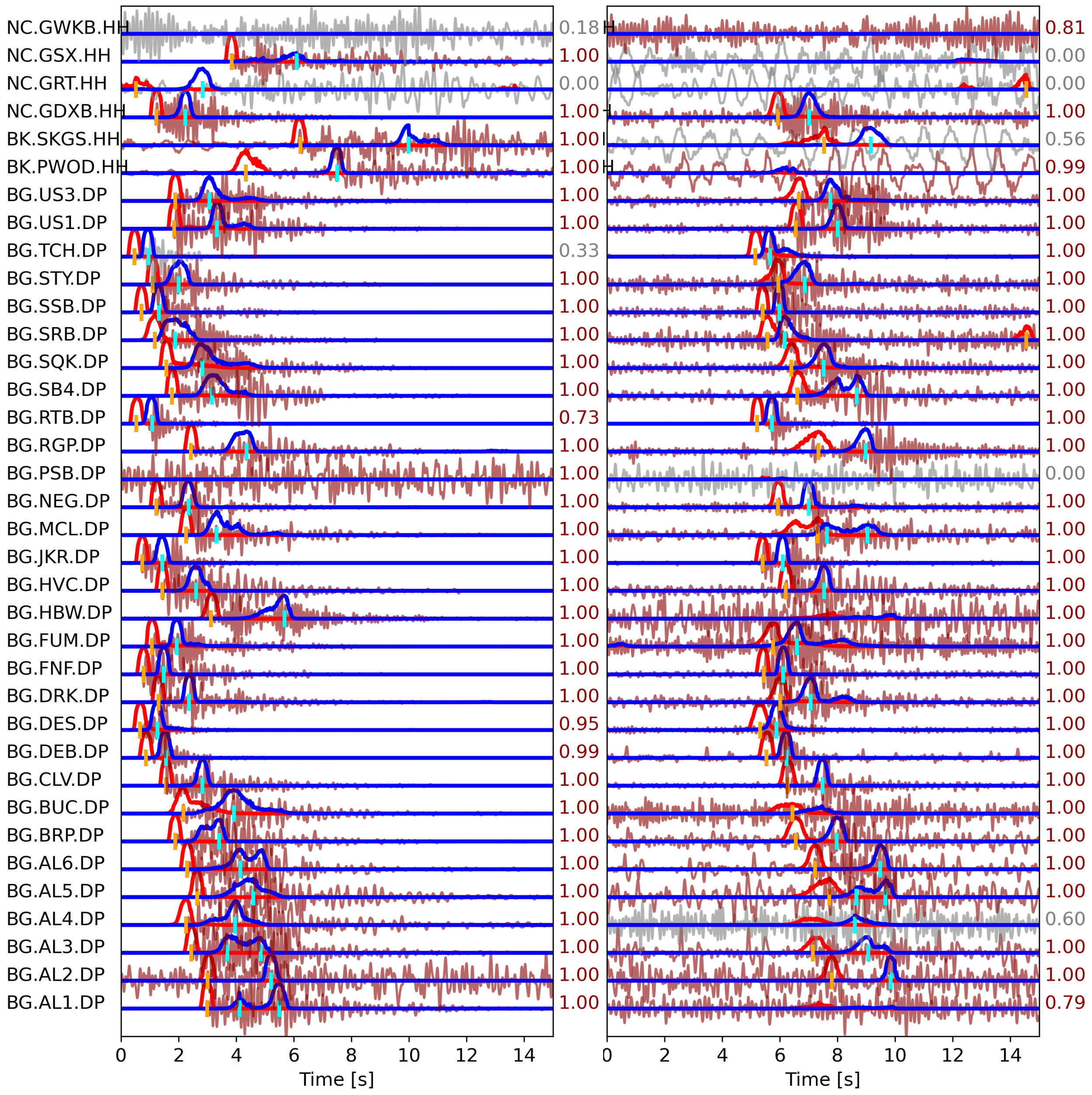

Supplementary Fig. 25: **Comparison of detection performance between QNO and PhaseNO for two microseismic events in the Geysers geothermal field.** The annotations on this figure are the same as those in the Supplementary Fig. 21.

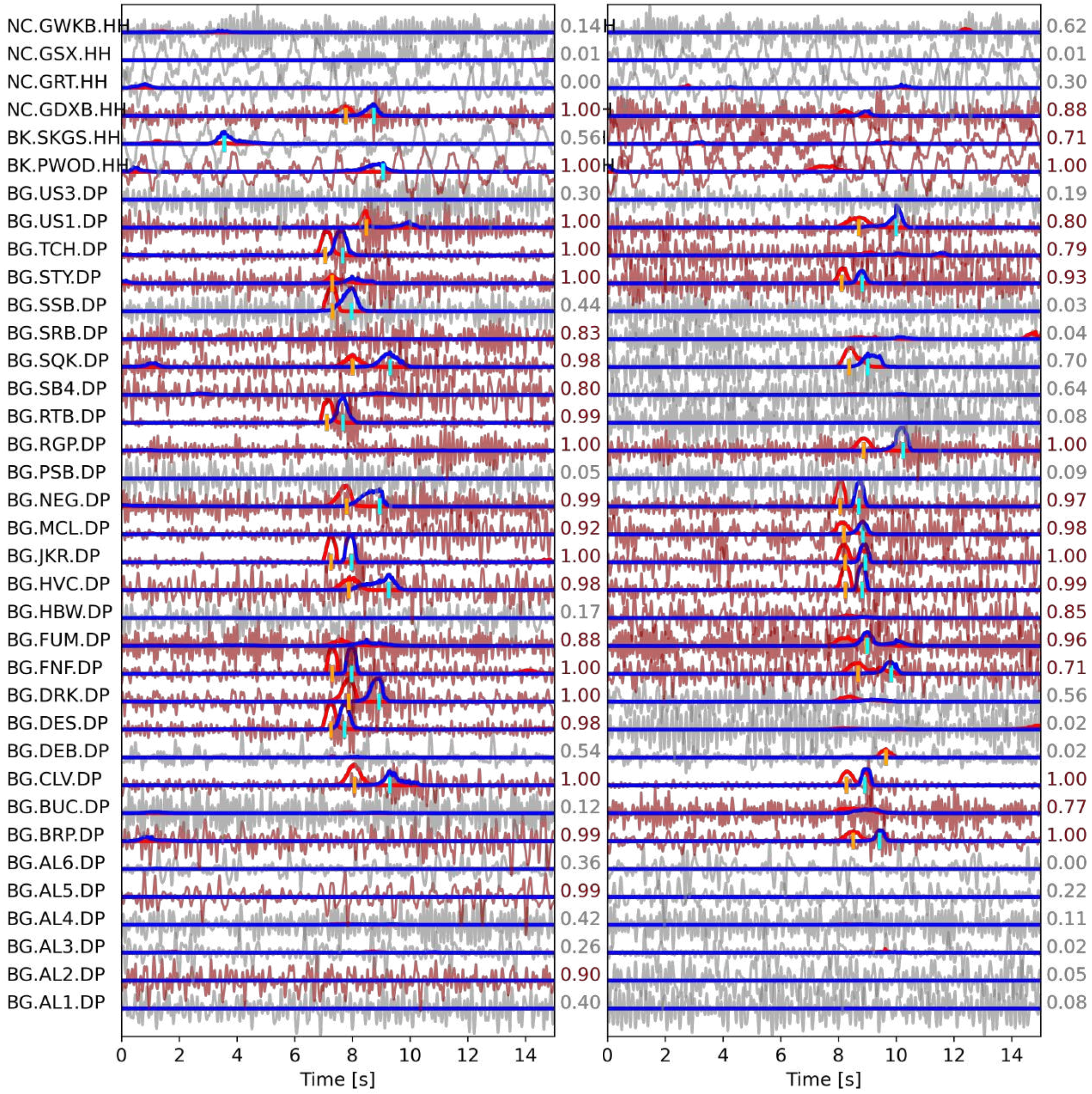

Supplementary Fig. 26: **Comparison of detection performance between QNO and PhaseNO for two microseismic events in the Geysers geothermal field.** The annotations on this figure are the same as those in the Supplementary Fig. 21.

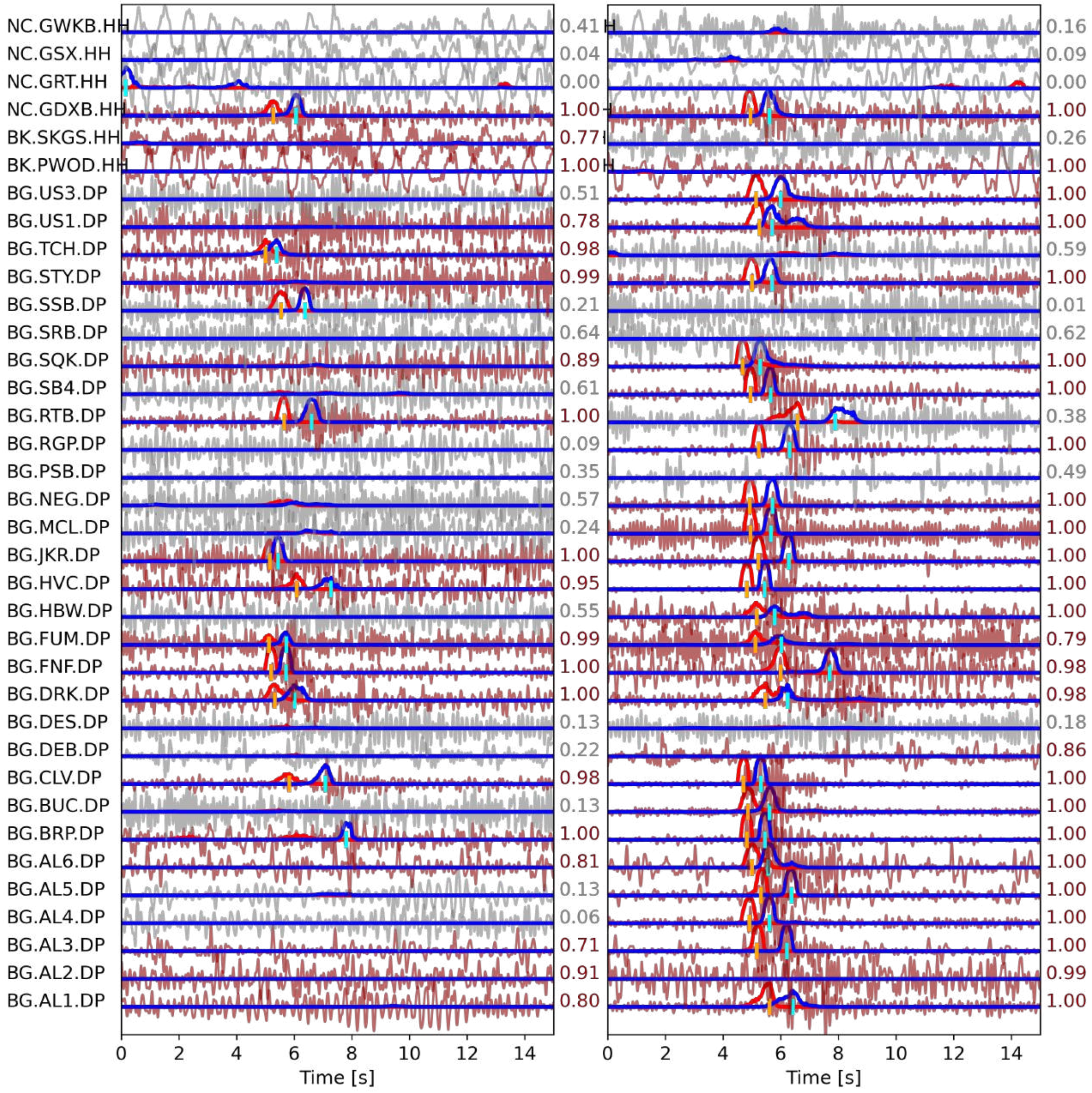

Supplementary Fig. 27: **Comparison of detection performance between QNO and PhaseNO for two microseismic events in the Geysers geothermal field.** The annotations on this figure are the same as those in the Supplementary Fig. 21.

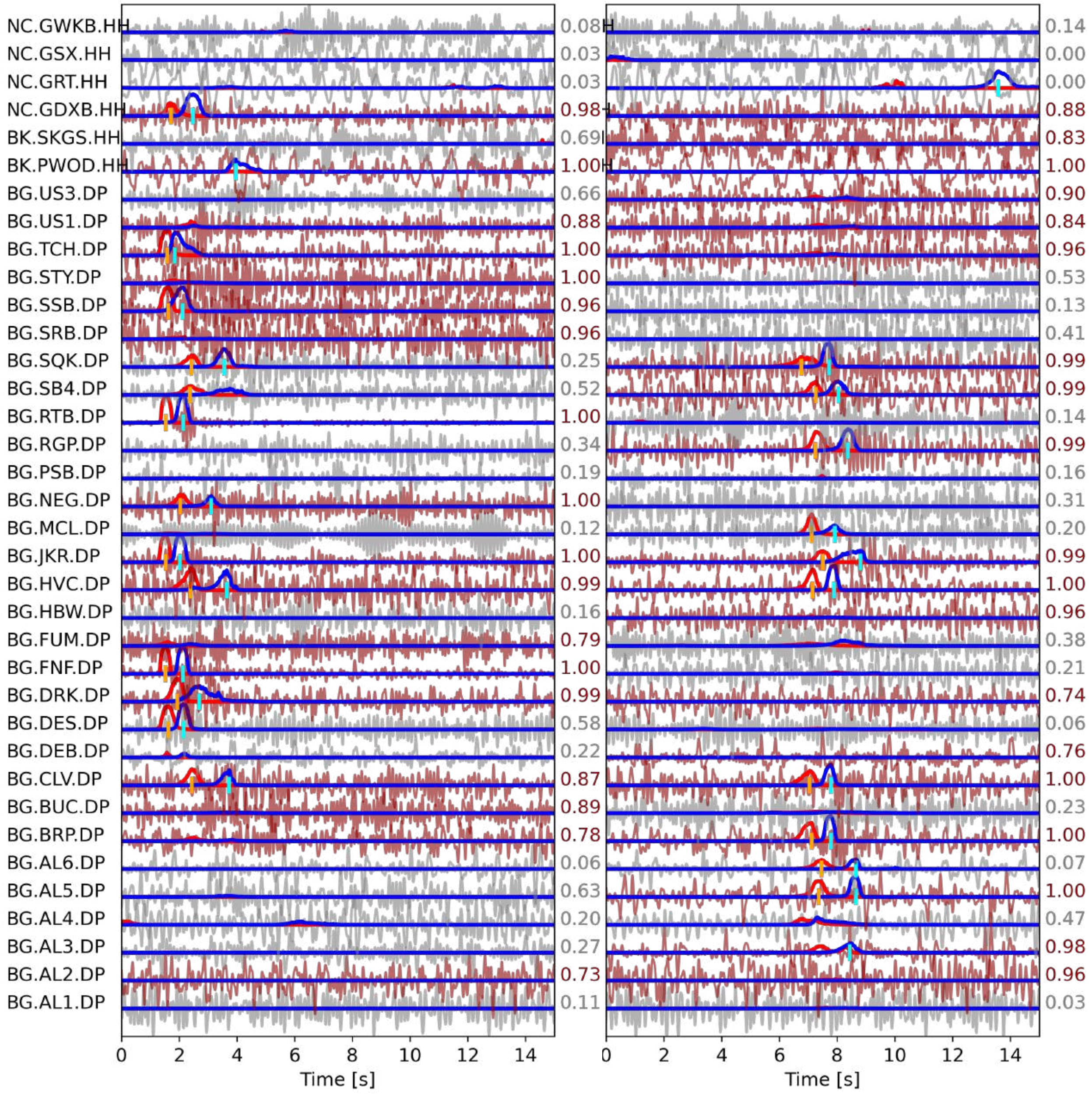

Supplementary Fig. 28: **Comparison of detection performance between QNO and PhaseNO for two microseismic events in the Geysers geothermal field.** The annotations on this figure are the same as those in the Supplementary Fig. 21.

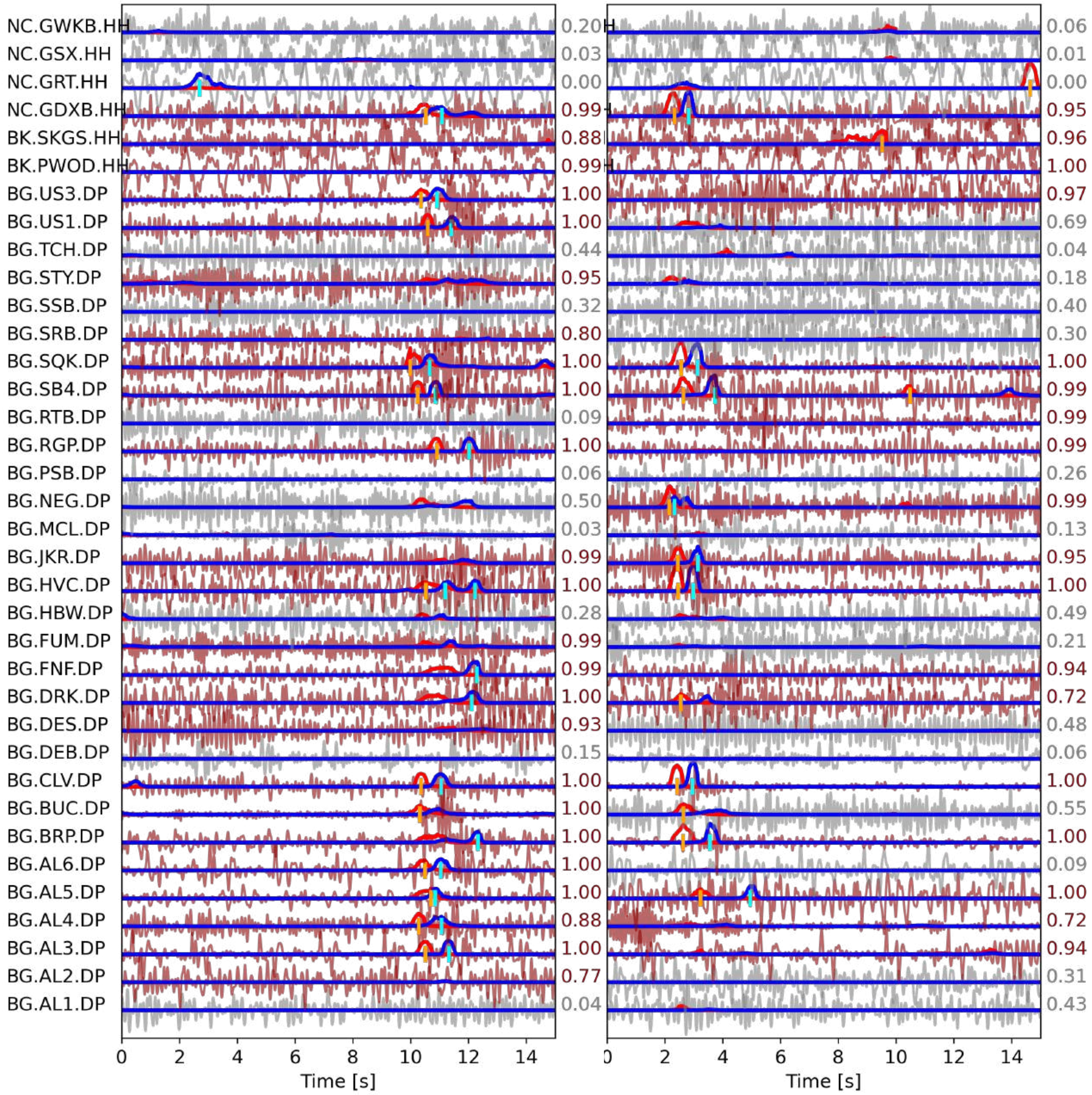

Supplementary Fig. 29: **Comparison of detection performance between QNO and PhaseNO for two microseismic events in the Geysers geothermal field.** The annotations on this figure are the same as those in the Supplementary Fig. 21.

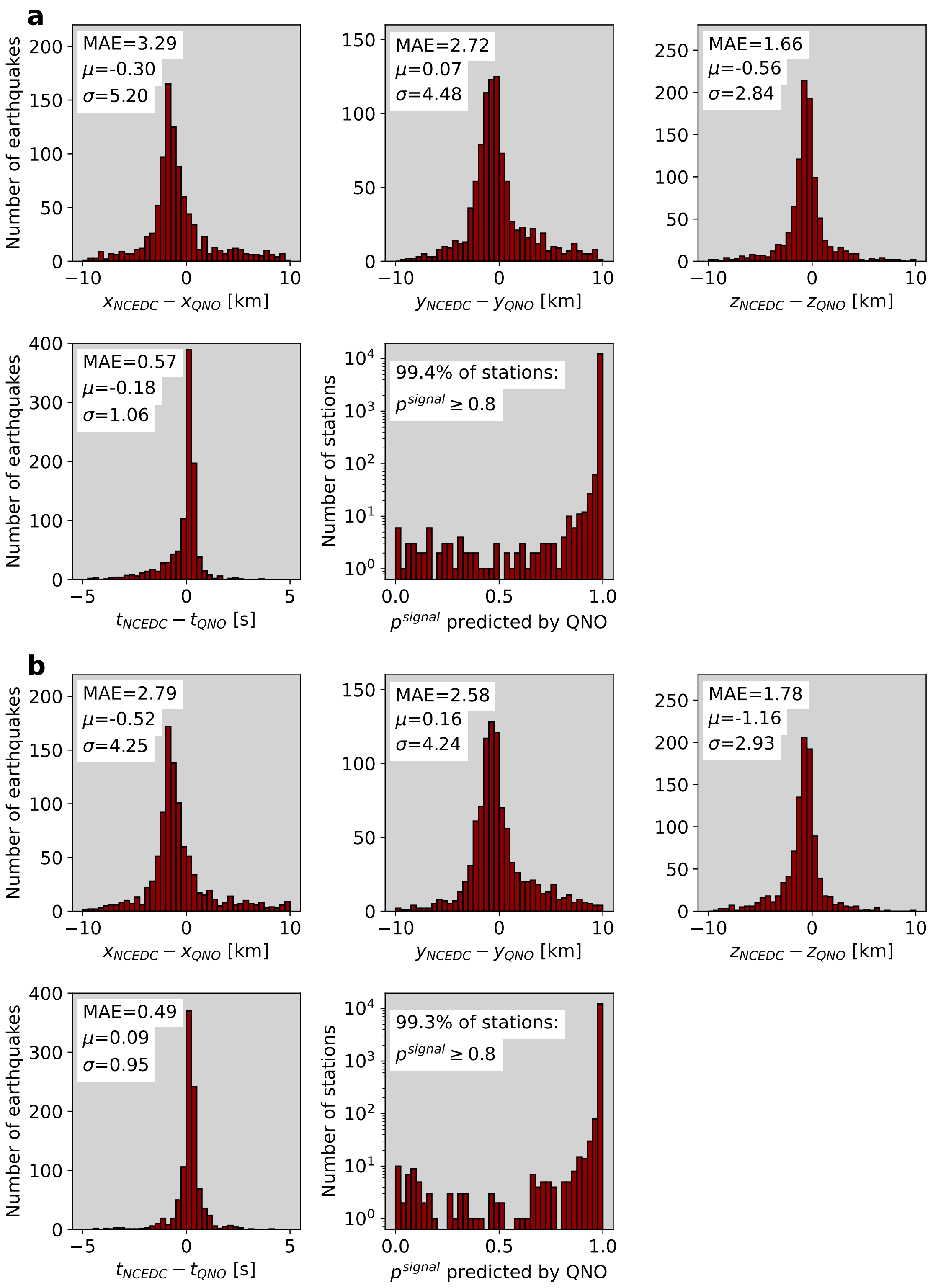

Supplementary Fig. 30: **Generalization performance of QNO evaluated on the NCEDC test dataset (Supplementary Fig. 4) with respect to the geographic distance threshold in the GNO layers.** The QNO model was trained with $D = 40\,\text{km}$. **a** Testing with $D = 20\,\text{km}$. **b** Testing with $D = 60\,\text{km}$. In both cases, the resulting histogram statistics are comparable to those obtained when testing with $D = 40\,\text{km}$ (Supplementary Fig. 5), indicating good generalization.

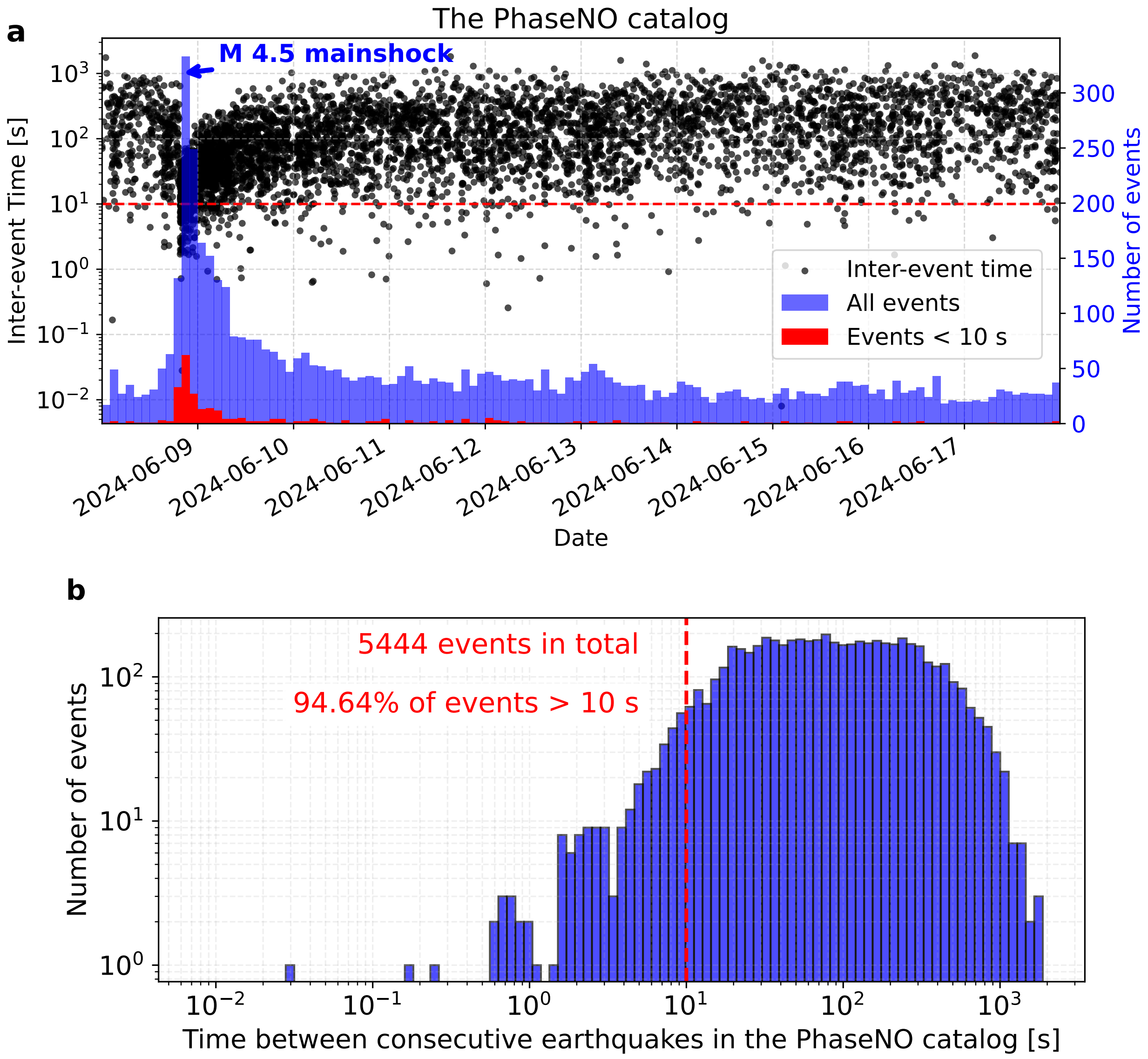

Supplementary Fig. 31: **The temporal variation of time intervals between consecutive earthquakes (inter-event times) in the Geysers geothermal catalog constructed using PhaseNO. a** Inter-event time of the catalog plotted against origin time (left vertical axis), overlaid with histograms of total event counts and counts of events with inter-event times shorter than 10 s (right vertical axis) shown in 2-hour bins. The horizontal dashed red line marks the 10 s threshold used to distinguish closely spaced events. The M 4.5 mainshock occurred at 19:34:29 UTC on 8 June 2024. **b** Log–log histogram of inter-event times. The vertical dashed red line marks the 10 s threshold used to identify closely spaced events, which are challenging for QNO models trained with a 15 s time window and applied with a 5 s overlap.

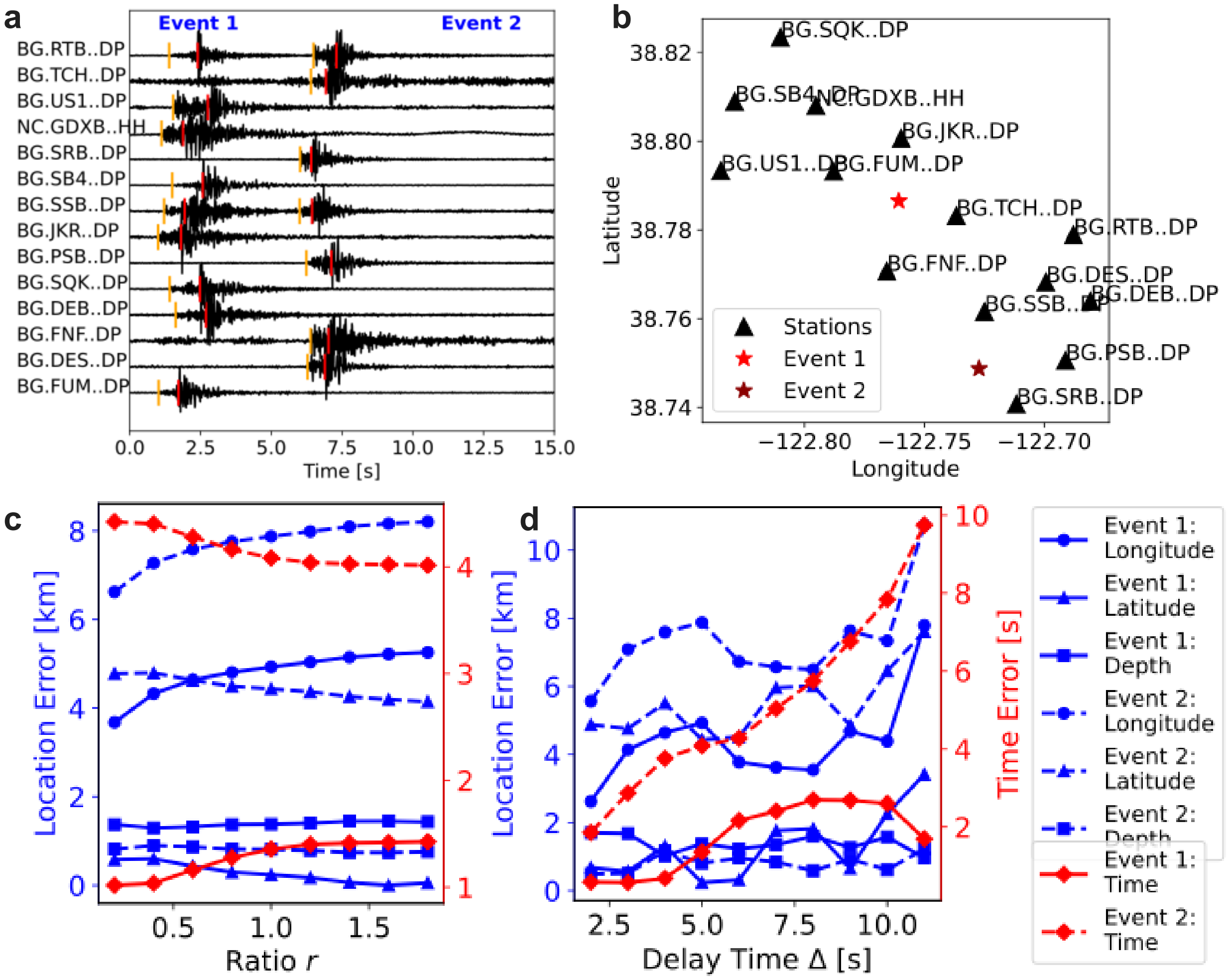

Supplementary Fig. 32: **Performance of QNO for two example events from the test dataset within a 15 s time window under varying amplitude ratios and delay times.** **a** Example of stacked waveforms with a 5 s delay between the two events and an amplitude ratio of 1. Manually picked P-phase (orange) and S-phase (red) arrival times are marked on the waveforms for reference. **b** Map showing all station locations and catalog locations of the two events. **c** Errors between QNO predictions and catalog locations for varying amplitude ratios with a fixed delay time of 5 s. **d** Errors between QNO predictions and catalog locations for varying delay times with a fixed amplitude ratio of 1.

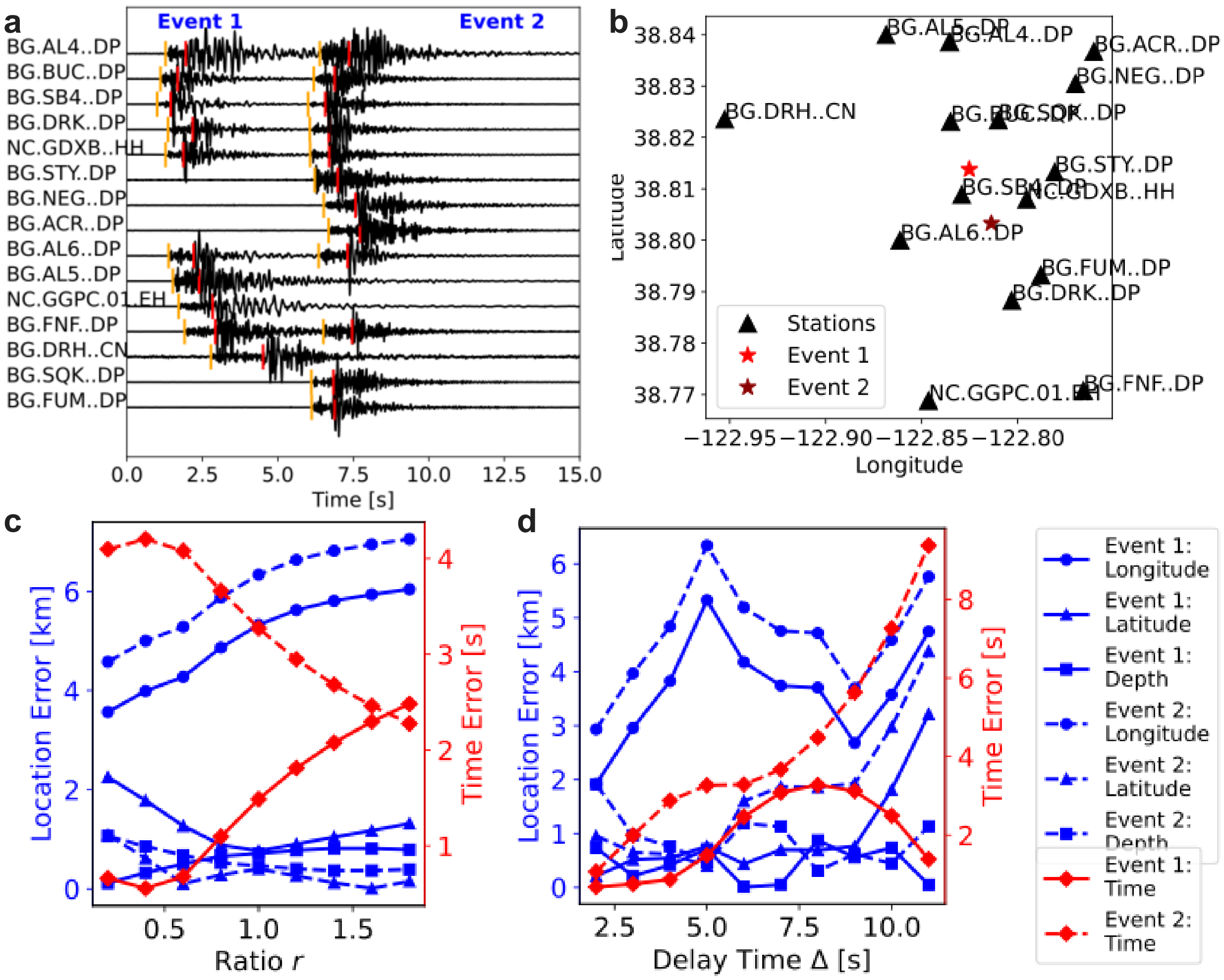

Supplementary Fig. 33: **Performance of QNO for another pair of example events from the test dataset within a 15 s time window under varying amplitude ratios and delay times.** Panel descriptions are the same as those in Supplementary Fig. 32.

Supplementary Table 1: List of stations used in the Geysers geothermal field dataset.

id	network	station	channel	longitude	latitude	elevation [m]	unit	component
0	BG	AL1	DP	-122.883	38.838	704.0	m/s	E,N,Z
1	BG	AL2	DP	-122.898	38.816	657.0	m/s	E,N,Z
2	BG	AL3	DP	-122.857	38.828	781.0	m/s	E,N,Z
3	BG	AL4	DP	-122.835	38.839	661.0	m/s	E,N,Z
4	BG	AL5	DP	-122.869	38.840	593.0	m/s	E,N,Z
5	BG	AL6	DP	-122.861	38.800	749.0	m/s	E,N,Z
6	BG	BRP	DP	-122.797	38.855	905.0	m/s	E,N,Z
7	BG	BUC	DP	-122.835	38.823	888.0	m/s	E,N,Z
8	BG	CLV	DP	-122.790	38.838	989.0	m/s	E,N,Z
9	BG	DEB	DP	-122.681	38.764	533.0	m/s	1,2,3
10	BG	DES	DP	-122.699	38.768	650.0	m/s	E,N,Z
11	BG	DRK	DP	-122.803	38.788	757.0	m/s	E,N,Z
12	BG	FNF	DP	-122.766	38.771	870.0	m/s	E,N,Z
13	BG	FUM	DP	-122.788	38.793	673.0	m/s	E,N,Z
14	BG	HBW	DP	-122.876	38.859	985.0	m/s	E,N,Z
15	BG	HVC	DP	-122.776	38.843	779.0	m/s	E,N,Z
16	BG	JKR	DP	-122.760	38.801	1067.0	m/s	E,N,Z
17	BG	MCL	DP	-122.823	38.855	961.0	m/s	E,N,Z
18	BG	NEG	DP	-122.770	38.830	922.0	m/s	E,N,Z
19	BG	PSB	DP	-122.691	38.751	697.0	m/s	1,2,3
20	BG	RGP	DP	-122.811	38.878	799.0	m/s	E,N,Z
21	BG	RTB	DP	-122.688	38.779	276.0	m/s	1,2,3
22	BG	SB4	DP	-122.829	38.809	327.0	m/s	E,N,Z
23	BG	SQK	DP	-122.810	38.823	639.0	m/s	E,N,Z
24	BG	SRB	DP	-122.712	38.741	912.0	m/s	1,2,3
25	BG	SSB	DP	-122.725	38.762	820.0	m/s	1,2,3
26	BG	STY	DP	-122.781	38.813	1112.0	m/s	E,N,Z
27	BG	TCH	DP	-122.737	38.783	951.0	m/s	E,N,Z
28	BG	US1	DP	-122.835	38.793	761.0	m/s	E,N,Z
29	BG	US3	DP	-122.840	38.805	502.0	m/s	E,N,Z
30	BK	PWOD	HH	-122.702	38.581	435.2	m/s	E,N,Z
31	BK	SKGS	HH	-123.078	38.689	637.5	m/s	E,N,Z
32	NC	GDXB	HH	-122.795	38.808	939.0	m/s	E,N,Z
33	NC	GRT	HH	-122.671	38.939	589.0	m/s	E,N,Z
34	NC	GSX	HH	-122.523	38.850	491.1	m/s	E,N,Z
35	NC	GWKB	HH	-122.492	39.053	847.6	m/s	E,N,Z
