# Supplementary material for: Microseismic monitoring with the quake neural operator
Source: Nat Commun. 2026 Jul 6;17:5677. doi: 10.1038/s41467-026-73965-6 (PMC13338072; doi:10.1038/s41467-026-73965-6)
Supplement: Supplementary file 2 — Transparent Peer Review file [file 41467_2026_73965_MOESM2_ESM.pdf]

nature portfolio

Peer Review File

Microseismic Monitoring with the Quake Neural Operator

Corresponding Author: Professor Hongyu Sun

This file contains all reviewer reports in order by version, followed by all author rebuttals in order by version.

Attachments originally included by the reviewers as part of their assessment can be found at the end of this file.

Version 0:

Reviewer comments:

Reviewer #1

(Remarks to the Author)
See attached.

(Remarks on code availability)

Reviewer #2

(Remarks to the Author)

(Remarks on code availability)
Review of "Microseismic Monitoring with Quake Neural Operator"

This manuscript introduces the Quake Neural Operator (QNO) for microseismic monitoring, aiming to detect and locate earthquakes directly from continuous seismic data without explicit phase picking. While the idea has merit, the manuscript suffers from several critical shortcomings that significantly undermine its novelty and impact. The QNO's architecture, which combines Fourier Neural Operators (FNO) for temporal data and Graph Neural Operators (GNO) for spatial data, closely mirrors that of Phase Neural Operator (PhaseNO)(Sun, et. al., 2023. Phase neural operator for multi-station picking of seismic arrivals. Geophysical Research Letters, 50, e2023GL106434. https://doi. org/10.1029/2023GL106434).

Here are some major concerns:

Incremental advancement: The QNO builds upon existing methodologies, particularly the PhaseNO, with only incremental improvements. The core concept of using neural operators for seismic event detection and location is already established. The manuscript fails to clearly articulate how QNO substantially differs from PhaseNO beyond parameter tuning and slight architectural adjustments.

Lack of novelty: The manuscript introduces a multi-task learning algorithm called the Quake Neural Operator (QNO), designed to directly detect and locate earthquakes from continuous data recorded by multiple stations in a seismic network with arbitrary geometry. However, the use of multi-task learning, combining classification and regression tasks, is not a novel concept. Such approaches are common in machine learning and have been previously explored in seismology. The manuscript does not provide sufficient evidence to demonstrate a significant advantage of this specific implementation over existing methods.

Limited Scope: The evaluation of QNO is primarily focused on the Geysers geothermal field. While this is a relevant application, the manuscript does not provide sufficient evidence to demonstrate the broad applicability of QNO to other regions or seismic settings. The specific geological and noise characteristics of the Geysers might favor QNO, and its performance may not generalize well to areas with different seismic signatures or network configurations.

Overstated claims: The abstract claims that QNO can reveal previously undetected seismic activities. However, the results presented only show a higher detection rate compared to routine catalogs and PhaseNO + GaMMA. There is no independent validation to confirm that these additional detections are indeed real events and not false positives.

Unconvincing benchmark: The comparison with PhaseNO is not entirely convincing. The manuscript mentions that QNO applies stricter criteria than PhaseNO, which could explain the higher number of detected events. Without a more detailed analysis of the trade-off between precision and recall, it is difficult to assess the true improvement offered by QNO.

Scalability and computational cost: The manuscript acknowledges that the computational cost of QNO scales quadratically with the number of stations. This is a significant limitation, especially for large seismic networks. The suggested solution of selecting a subset of stations may compromise the detection performance and undermines the advantage of using network-wide information.

Recommendation
I recommend rejecting the manuscript in its current form. The authors need to:

Clearly articulate the novel contributions of QNO and provide a more thorough comparison with existing methods, especially PhaseNO.

Provide more robust validation of the detected events, using independent datasets or techniques.

Address the limitations of QNO, including its computational cost and potential for overfitting to specific seismic settings.

Demonstrate the generalizability of QNO to other regions and seismic networks.

Without substantial revisions that address these concerns, the manuscript does not offer sufficient scientific advancement to warrant publication.

Reviewer #3

(Remarks to the Author)
The manuscript presents a novel deep learning framework, the Quake Neural Operator (QNO), for direct detection and location of microseismic events from continuous waveform data, bypassing the traditional reliance on seismic phase picking. The method integrates Fourier Neural Operators for temporal encoding and Graph Neural Operators for spatial relationships across stations, enabling it to work with arbitrary seismic network geometries. The multi-task learning framework jointly solves detection (classification) and location (regression), which improves performance by leveraging shared representations.
The model is benchmarked against state-of-the-art approaches (e.g., PhaseNO, SeismicGNN) and shows superior performance, particularly under noisy conditions.
The application to The Geysers geothermal field demonstrates QNO's capability to significantly expand earthquake catalogs beyond what existing methods provide.

Comments:

1. The QNO architecture is highly original in the field of microseismic monitoring.
While the novelty is clear, the authors could strengthen the discussion around the limitations of operator learning in handling complex or overlapping seismic events, especially when dealing with thousands of events as in the dataset mentioned.

2. The method assumes one event per 15-second window (P6, P21), which may limit applicability in aftershock-rich periods or during intense hydrofrac phase. While overlapping windows are proposed as a workaround, more discussion is needed on its limitations and potential alternatives.
Include more detail on how QNO performs with multiple closely spaced events, e.g. in the Supporting Information.

3. Extensive benchmarks against SeismicGNN and PhaseNO show QNO's superiority, especially in low SNR scenarios.
The ground-truth catalog from NCEDC is used as a reference, but with limited completeness (only 45 events in 5 days vs. > 3000 detected by QNO). This makes true precision/recall metrics ambiguous.
I would suggest to add a more detailed error analysis of false positives and false negatives, possibly via manual inspection or alignment with external catalogs, to validate the increase in detection rate.

4. Whereas the Geysers case study is compelling and demonstrates the model’s utility in geothermal monitoring, consider discussing how QNO could support real-time monitoring pipelines, especially in industrial applications (e.g., geothermal energy or carbon sequestration)

5. The manuscript is well-structured and rich in illustrations, however, figure legends (e.g., Figs. 4–9) could be more concise. Some are very long and takes whole paragraphs.

(Remarks on code availability)
The availability of code and pretrained models upon publication is essential for reproducibility and community adoption.

Version 1:

Reviewer comments:

Reviewer #1

(Remarks to the Author)
See attached "Referee_Report_NCOMMS_25_07335A.pdf".

(Remarks on code availability)

Reviewer #2

(Remarks to the Author)
One approaches this manuscript with the hopeful anticipation of a truly "novel deep learning algorithm", as boldly proclaimed by the authors, only to find a familiar landscape thinly disguised by semantic gymnastics. The Quake Neural Operator (QNO), while presented with a flourish, struggles to convincingly differentiate itself from its purported predecessors, particularly the Phase Neural Operator (PhaseNO). Indeed, I still identify this central flaw as "incremental advancement" and a "lack of novelty".
The authors declare QNO a "multi-task neural operator" that bypasses "explicit phase picking" to directly build earthquake catalogs. Yet, the very core concept of employing neural operators for seismic event detection and location is, by the authors' own admission, "already established". We are told QNO uses Fourier Neural Operators (FNO) for temporal data and Graph Neural Operators (GNO) for spatial data, much like PhaseNO. The distinctions offered—that PhaseNO is a "per-station phase picker" while QNO performs "end-to-end event detection and location", or that QNO is "multi-task" unlike PhaseNO's "single-task" approach—feel less like groundbreaking innovation and more like a mere reframing of the problem space, a slightly different configuration of existing, well-known deep learning components. As I pointed out, "multi-task learning... is not a novel concept" in machine learning, and its application in seismology has been "previously explored".
The claim of a "simpler architecture" for QNO compared to PhaseNO's U-shaped design hardly constitutes a paradigm shift. One might even wonder if "simpler" implies less capable, or merely a different flavor of an already developed methodology. While the paper touts QNO's ability to operate "without the need for explicit phase picking", it's worth noting that other deep learning approaches already strive for full automation or integrate picking with subsequent location steps. The novelty, in this regard, appears to be a matter of degree rather than a fundamental breakthrough.
Even the boasted "robust detection at low SNR" feels like an incremental gain building on the foundation of neural operators already demonstrated to enhance seismic monitoring. While a higher event count in the Geysers geothermal field is reported, the true precision and recall, especially in comparison to actual (and often incomplete) catalogs, remain ambiguous. The "significant advantage" over existing methods remains largely unconvincing, primarily because the underlying mechanisms and conceptual framework are not sufficiently distinct from prior work in the field.
In sum, this paper presents a variation on an existing theme, leveraging established neural operator architectures and multi-task learning in a slightly different configuration for seismic event monitoring. While operational refinements and performance improvements are always welcome, the work falls short of delivering the truly "novel" and "substantially different" contribution it professes, echoing the sentiment of "incremental advancement" rather than a significant leap forward.

(Remarks on code availability)

Reviewer #3

(Remarks to the Author)
The authors have addressed all my comments and those from the other reviewers in a thorough and convincing manner. I think that the revisions significantly improve the manuscript and clarify key aspects of the methodology and its applications.

Notably, the authors have added a larger number of events to the analysis - this strengthens the validation of QNO's performance and further demonstrates its robustness. The discussion on the model's applicability, including limitations and potential for real-time monitoring, has also been improved and is now more comprehensive.

The manuscript is now much stronger, both in terms of technical content and clarity of presentation.

I just have one minor point: In the supplementary materials, some figures have non-optimal formatting (oversized fonts, label overlaps). While this does not detract from the main results, improving figure quality would enhance readability. Probably the authors did not have much time to invest before the deadline for the revision.

I recommend acceptance.

(Remarks on code availability)
The code is not available but the authors claim that it will be available upon acceptance

Version 2:

Reviewer comments:

Reviewer #1

(Remarks to the Author)

(Remarks on code availability)

Open Access This Peer Review File is licensed under a Creative Commons Attribution 4.0 International License, which permits use, sharing, adaptation, distribution and reproduction in any medium or format, as long as you give appropriate credit to the original author(s) and the source, provide a link to the Creative Commons license, and indicate if changes were made.
In cases where reviewers are anonymous, credit should be given to 'Anonymous Referee' and the source.
The images or other third party material in this Peer Review File are included in the article’s Creative Commons license, unless indicated otherwise in a credit line to the material. If material is not included in the article’s Creative Commons license and your intended use is not permitted by statutory regulation or exceeds the permitted use, you will need to obtain permission directly from the copyright holder.
To view a copy of this license, visit https://creativecommons.org/licenses/by/4.0/

Response to Reviewers

(NCOMMS-25-07335)

Table of Contents

Summary:* *2

Reviewer #1* *3

Reviewer #2* *22

Reviewer #3* *29

Summary:

We thank the Editor and Reviewers for their constructive feedback. We have substantially revised the manuscript to enhance clarity, rigor, and robustness. We carefully considered all comments and added targeted analyses and clarifications where needed. The line numbers below refer to the clean revised manuscript. Major revisions include:

- We added Supplementary Note 1: Preliminaries on Neural Operators for readers unfamiliar with the topic. We also provide a detailed description of operations on the FNO and GNO layers in the Methods when describing the QNO architecture (Lines 427–458).
- We systematically study QNO's behavior when two events occur within the same time window (see Supplementary Note 3).
- We compare two data-splitting strategies for the NCEDC dataset: a temporal split by origin time and a random split via shuffled indices. Details are provided in Supplementary Note 4.
- We added a subsection in the main text, Generalization across Seismic Network Configurations, showing QNO's generalization under variations in station number, station geometries, and temporal resolution (Lines 197–215).
- We quantify the computational cost of QNO and demonstrate its suitability for real-time monitoring (Lines 291–319; section Computational Cost of QNO).
- We added five additional days to the Geysers geothermal dataset, including an M 4.5 mainshock, and benchmarked QNO and PhaseNO on the aftershock-rich sequence.
- The Supplementary Information has been substantially expanded to provide more details about the method and more comprehensive analyses of the results.
- We clarified how QNO differs from operator-learning approaches based on phase picking, i.e., PhaseNO (Lines 343-354).
- QNO software and source data are provided with this revision.

Below, we provide a point-by-point response. Reviewer comments appear in black; our responses appear in blue.

Reviewer #1

This work introduces a new algorithm to build earthquake catalogs from seismic data without phase picking. Creating such catalogs is crucial to the study of natural earthquakes, but also and especially for monitoring of seismicity induced by geothermal energy development, mining, carbon capture and storage etc. The manuscript is carefully written and discusses its results thoroughly. Following are my Primary and Secondary comments.

We thank the reviewer for the positive review and constructive feedback. We appreciate the recognition of our method's potential and the comments on clarity. We have revised the manuscript accordingly and address the primary and secondary points below.

Primary comments

31–41 I assume these are useful citations. You should also summarize to what extent the DL approaches are quantitatively more accurate than the human ones, if that is true, aside from cost and time issues.

Phase picking in seismic monitoring has traditionally been carried out by human analysts, particularly for natural earthquakes. Over the past few decades, manual picks have provided a large amount of high-quality training data for deep learning-based phase pickers. As a result, most deep learning models are trained on human-labeled data, and quantitative evaluations show that their picking accuracy is comparable to that of human analysts. Specifically, the mean absolute errors (MAEs) in P- and S-phase arrival times between human picks and a recent published deep-learning model, PhaseNO (Sun et al., 2023) are 0.05 s and 0.09 s, respectively.

Beyond arrival-time precision, deep learning models significantly enhance the detection of small earthquakes that are often missed by manual processing. For example, EQTransformer, a transformer-based phase picker, achieved more than a twofold increase in the number of detected events using only 18 stations, compared to a traditional catalog constructed from manually picked phases using 57 stations (Mousavi et al., 2020). These models also offer additional advantages such as automation, generalization to new regions, and computational efficiency.

However, most of these advances rely on phase picking, which may still miss very low signal-to-noise ratio (SNR) signals or events lacking clear phase onsets, such as microseismic signals. In such cases, template matching, which detects events by cross-correlating continuous waveforms with pre-existing templates, is often used to improve detection capability. Motivated by these limitations, this work introduces QNO to overcome the challenges of detecting and locating events that conventional picking-based methods may fail to identify.

QNO does not rely on phase picking. Instead, it detects events directly from raw waveforms, substantially improving detection capability. Since arrival times are not available, QNO estimates the origin time and location of detected events directly from the waveform data across multiple stations. In summary, QNO enables the construction of more complete earthquake catalogs directly from continuous seismic data.

We have added a brief summary in the revised manuscript to address this point. Specifically, we now include the quantitative comparison between PhaseNO and manual picks in terms of arrival-time precision, highlighting the mean absolute errors in P- and S-phase arrival times (0.05 s and 0.09 s, respectively) as evidence of comparable accuracy between deep learning models and human analysts.

Lines 40-47 of the revised manuscript: "*As an example of a deep learning-based phase picker, EQTransformer achieved more than a twofold increase in the number of detected events over the same time period, using only 18 stations, compared to a traditional catalog built from manually picked phases using 57 stations (Mousavi et al., 2020). In addition to enhanced detection capability and efficiency, deep learning models also achieve arrival-time precision comparable to that of human analysts. Specifically, the mean absolute errors (MAEs) in P- and S-phase arrival times between human picks and PhaseNO are 0.05 s and 0.09 s, respectively (Sun et al., 2023).*"

80 "neural operator architectures", will we read a summary of what that means? Most readers won't know. How is a NO different from other NNs?

We agree that a brief review of neural operators in this manuscript would benefit readers who may not be familiar with the concept. In response, we have added a review section titled "*Supplementary Note 1 Preliminaries on Neural Operators.*" at the beginning of the *Supplementary Information*.

In this new section, we explain that neural operators extend conventional neural networks by learning mappings between function spaces, rather than between finite-dimensional vectors. This makes them particularly suitable for modeling physical systems described by partial differential equations (PDEs). Here, we extend their application beyond PDE solving to seismic monitoring. We also describe how neural operators approximate integral kernel operators and how they differ structurally and conceptually from traditional neural networks. Specifically, we highlight that neural operators are designed to generalize across various input discretization and domain geometries, which makes them a compelling choice for problems with irregular or variable input structures, such as those found in seismic monitoring.

We hope this revision provides sufficient context and clarity for readers unfamiliar with neural operators.

84–121 None of this belongs in “Results”, because it’s all “Methods” exposition.

According to the journal’s article structure guidelines, “The main text of an Article should begin with a section headed Introduction [...] followed by sections headed Results, Discussion (if appropriate) and Methods (if appropriate).

Since this is a methods-focused contribution, the core of the study lies in the development of the QNO. While the Methods section (at the end) provides full details, we believe that summarizing the architecture and design of QNO at the beginning of the Results section is necessary and appropriate, as it constitutes a key outcome of the paper and serves to contextualize the subsequent experiments and evaluations.

Nevertheless, we have reviewed the organization to ensure clarity and are open to further restructuring if the reviewer and editor believe it would improve readability.

94–95 “QNO can be evaluated on varying grids without requiring model retraining” is a very strong claim, and seems unlikely on its face. Justify it.

We have revised the manuscript to clarify and justify our claim, and we now provide additional explanations and experiments to support the generalization capability of QNO without retraining.

First, each sample in our dataset is associated with a unique station geometry (i.e., a different seismic network configuration). While Figure 3 shows all stations available in the

dataset, each event is recorded by only 8 to 32 stations (Figure 2), and the station sets are largely non-overlapping across events. Moreover, during training, we randomly add noise stations to each sample; these noise stations have random spatial coordinates and real noise waveforms (from the STEAD dataset). This further increases the diversity of seismic network geometries seen by the model during training. In the Supplementary Information, we provide two examples illustrating the station distribution and waveforms for individual samples.

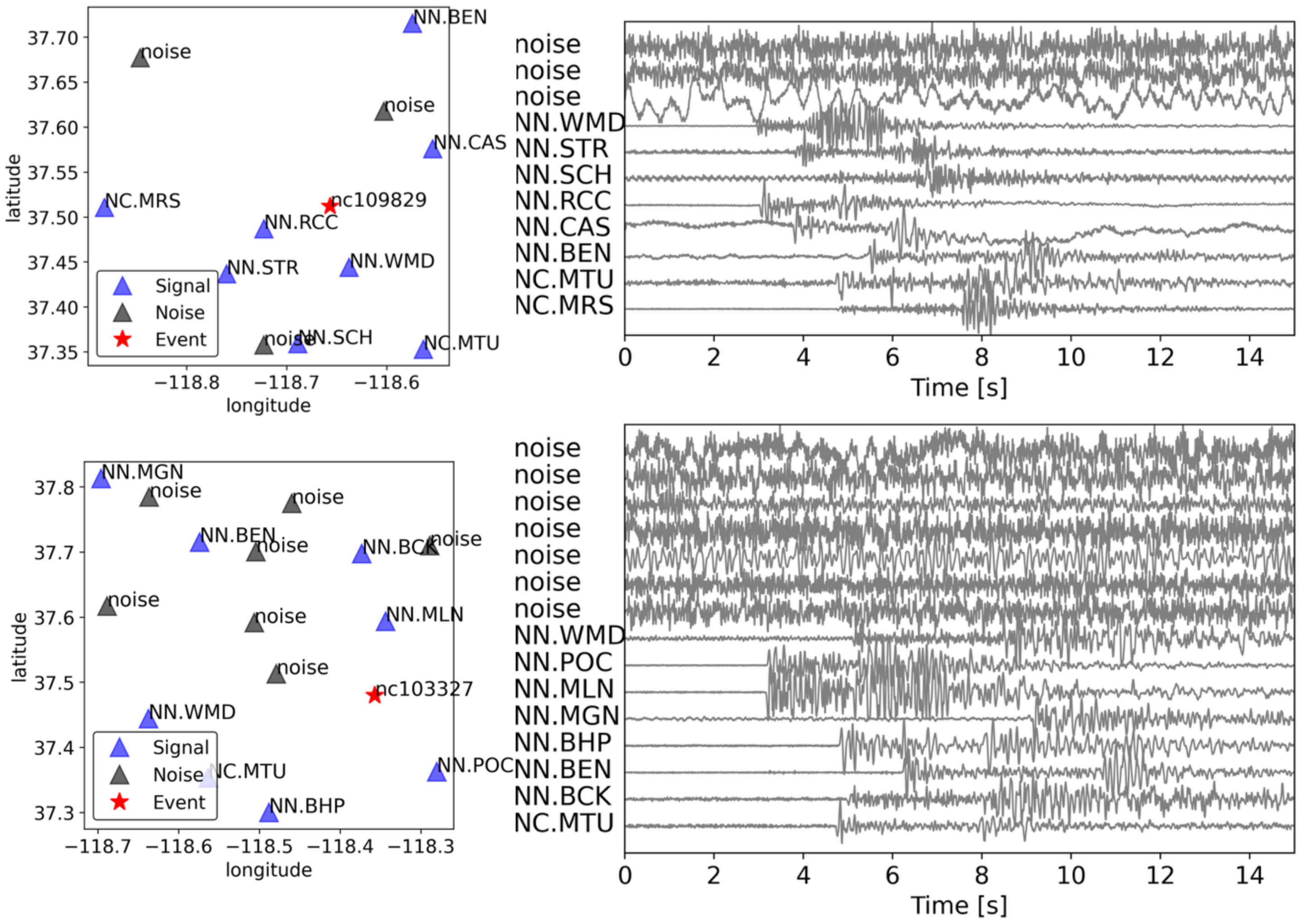

Supplementary Figs. 1 and 2: Examples of two training samples.

In the neural operator framework, the model learns a mapping between function spaces, rather than grid-fixed representations. The station geometry can be viewed as a discretization of the spatial domain, with each station serving as a sampling point of the input function. Since each sample in our dataset has a unique station geometry, yet QNO

can still detect and locate events accurately, as shown in Figures 3 and 4. This supports our claim that QNO generalizes well across spatial domains.

Regarding temporal resolution, QNO can also generalize to samples with different time sampling rates, as long as the input remains uniformly sampled (a requirement due to the use of FFT in the FNO layers). To support this claim, we added a new experiment in the revised manuscript. While our main experiments use 15-second input seismograms sampled at 100 Hz (resulting in 1500 time steps), we apply the trained QNO model, without retraining, to test inputs upsampled to 3000 time steps (i.e., a 200 Hz sampling rate). As shown in Supplementary Fig. 17, QNO maintains good performance under this higher temporal resolution, further demonstrating its generalization capability.

We have added a subsection, "*Generalization across Seismic Network Configurations*," to the Results (lines 197–215) presenting these justifications. We also added Supplementary Note 1, "*Preliminaries on Neural Operators*," to provide background and references on resolution invariance in operator learning (see Supplementary Note 1 in the Supplementary Information).

In addition, we revise the sentence here to clarify this point (Lines 106-109 of the revised manuscript): "*As a type of Neural Operator, QNO learns a resolution-invariant mapping between function spaces rather than grid-specific representations. This allows QNO to be evaluated on inputs with different spatial or temporal discretizations without requiring retraining.*"

Fig. 2 (a) The legend has 3 colors but the bars have 4.

The histogram indeed uses three categories, each corresponding to a different color in the legend. However, since the histogram bars are plotted with partial transparency, overlapping between categories in the same bin can visually produce additional blended colors. This can give the appearance of more than three distinct bar colors in some bins, even though only three are used.

(b) The "Date" bins are like 0.7 years wide.

The x-axis is labeled as "Date" and divided into 50 uniform bins across the entire time span of the dataset. As a result, each bin does not correspond to an exact calendar year but instead spans approximately 0.7 years. This was done to balance resolution and visual clarity. Source data used to plot this figure are provided.

(c) “Both training and test datasets cover a wide range of depth and magnitude” appears to be true, but not impressively so. The way the training, validation, and test datasets were sampled assumes stationarity that is dubious. Instead, re-sample by repeatedly generating uniformly random indexes into the data, with replacement for each dataset, but empty intersection between datasets. Lines 361–363 don’t justify this simple splitting.

Our original decision to split the dataset based on the time of occurrence was motivated by a practical consideration: in real-world applications, historical seismicity is often used to train models, which are then applied to detect or locate future events. Therefore, constructing the training dataset from earlier earthquakes and applying the trained model to later events is, in our opinion, a natural use case in seismology.

We would like to clarify that our use of a temporal split was intentional and not based on an assumption of stationarity. On the contrary, we adopted this strategy to reflect real-world deployment scenarios, where models are trained on past events and applied to future data that may exhibit distribution shift. This setting provides a more challenging but realistic evaluation of model generalization, as the underlying data distribution may change over time due to evolving network conditions, noise levels, or catalog completeness.

Following the reviewer’s suggestion, we now adopt a random shuffling strategy to construct the training, validation, and test datasets. Specifically, we randomly permute the indices of all available events and select the number of events in each subset to match those shown in Figure 2 of the main text, ensuring no overlap between them. This random split mitigates the effects of temporal distribution shift and ensures that all three datasets are drawn from the same overall distribution (see Supplementary Fig. 4).

We have retrained QNO using the randomly sampled training set and evaluated its performance on the corresponding test set. As shown in Supplementary Fig. 5, the model achieves better performance under the random split, consistent with expectations since the distribution shift between training and test sets is reduced.

We have added a section, “*Comparison of data-splitting strategies for the NCEDC dataset*,” that explains the rationale for both strategies and discusses the implications of distributional shift (see Supplementary Note 4 in the Supplementary Information).

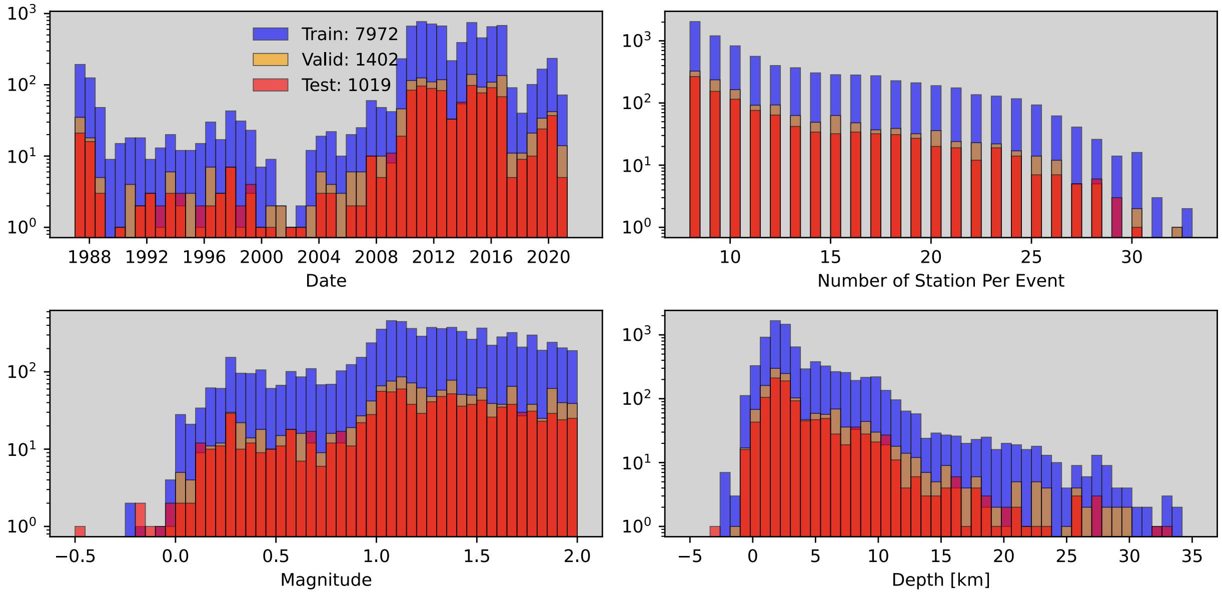

Supplementary Fig. 4: NCEDC seismic dataset split using randomly shuffled indices.

(d) Why are some depths negative? If "depth" is referenced to sea level, then say so. Also address at Line 409.

The "depth" values are referenced to sea level. In some regions of California, seismic events occur at elevations above sea level due to high topography (e.g., mountain ranges). In such cases, earthquake locations above sea level are recorded with negative depth values. We have clarified this at Lines 497-499 in the revised manuscript:

"Depths are referenced to sea level; negative values indicate earthquakes occurring above sea level in high-elevation regions (e.g., mountains in California)."

Fig. 3 is hard to interpret because one can't tell how many markers overlap each other. Instead, make 3 plots side-by-side with station markers and land-sea & state boundaries, without topographic coloring, and overlay contours of the KDE of the QNO and NCEDC results in the 1st 2 plots, and of the difference of KDEs in the 3rd plot.

Following the reviewer's guidance, we generated a new figure showing side-by-side contour plots of kernel density estimates (KDEs) for the NCEDC catalog and QNO-predicted epicenters, as well as their difference. This visualization highlights spatial patterns and potential biases in location predictions. The updated KDE figure is currently included as Supplementary Fig. 6 in the Supplementary Information.

In the main text, we chose to present individual epicentral locations to be consistent with the standard visualization style in earthquake monitoring studies. However, we fully agree that the original overlay of catalog and predicted locations in a single panel was suboptimal. In the revised Figure 3, we now use three panels to separately show:
(a) the NCEDC catalog locations, (b) the QNO-predicted locations, and (c) the location residuals as arrows pointing from catalog to predicted epicenters. If the reviewer believes that the KDE-based visualization should appear in the main text, we will consider moving it accordingly.

In addition, the source data used to plot Figure 3 are provided with the revised manuscript. Readers interested in further analysis can download these data if the manuscript is published.

Fig. 5 (a) The caption refers to “probabilities predicted by QNO and PhaseNO” but the plot legend shows only “PhaseNO” and “manual”.
(b) Do “Z, N, E” denote depth, north and east components, or what? Say in Caption.
(c) Why is time error not monotonic vs SNR? Discuss.

(a) As noted in the caption, the probabilities predicted by QNO are not included in the plot legend but are displayed in red beneath the corresponding station names: “*The probabilities (p) predicted by QNO for each input station are displayed in red beneath the corresponding station names.*”

(b) We have revised the caption to clarify the meaning of “Z”, “N”, and “E” as follows: “'Z', 'N', and 'E' indicate the vertical, north-south, and east-west components of the three-component seismic waveform at each station.”

(c) We thank the reviewer for the insightful question. While the overall error increases with decreasing SNR, they do not follow a strictly monotonic trend. This is likely due to the use of real, structured noise recordings, which contain non-Gaussian features such as microseisms and site effects that may interfere with waveform features in complex ways. Since QNO integrates information nonlinearly across multiple stations, certain noise realizations may degrade some stations more than others, leading to locally smaller or larger errors. To better understand this effect, we repeated the same experiment using idealized Gaussian noise. The results (now added as Supplementary Fig. 16 in Supplementary Information) show a more monotonic increase in error, supporting our interpretation. We have added this discussion to the revised manuscript, Lines 185-196:

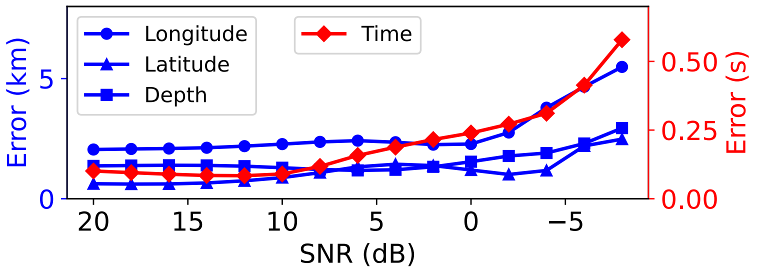

Supplementary Fig. 16: Detection and location performance for event nc73384250 under varying Gaussian noise levels.

"Interestingly, the predicted time and location errors do not always exhibit a strictly monotonic increase as the SNR decreases. This non-monotonic behavior is likely due to the use of real noise recordings, which may contain microseisms, site effects, or instrument-related transients that introduce structured perturbations into the waveforms. Since noise is added at the waveform level and the QNO model integrates information nonlinearly across multiple stations, certain noise realizations may interfere less destructively with feature extraction, resulting in locally smaller errors. To further investigate this effect, we repeated the experiment using idealized Gaussian noise with the same SNR levels. The results in the Supplementary Fig. 17 show a more monotonic increase in errors, confirming that structured real noise can introduce irregular, non-monotonic effects on performance. This observation highlights QNO's robustness to diverse noise structures and its ability to leverage inter-station correlations, even under complex real-world conditions."

Fig. 7 Same comment as for Fig. 3, above.

We generated a new figure showing side-by-side contour plots of KDEs for the NCEDC catalog and QNO-predicted epicenters, as well as their difference. The updated KDE figure is currently included as Supplementary Fig. 19 in the Supplementary Information.

273–274 "this hyperparameter can be optimized based on the inter-event times from past seismic history" —say more about this. Do you mean "optimize" in the loose sense that one can obtain better results by some empirical hyperparameter assignment, or in the tight sense that a certain hyperparameter value minimizes some loss function?

We agree that "optimize" is not meaningful in this context, and we planned to use "select": Since there is no ground truth or loss function to guide this choice, the inter-event time threshold can be selected empirically based on past seismic history.

However, during revision, the entire paragraph has been deleted. Instead, we discuss the selection of waveform length in more detail. Please refer to Lines 384-394 in the revised manuscript.

292–293 “enabling the trained QNO to generalize to various seismic network geometries” is ambiguous. Do you mean that it can be trained and operate on any arbitrary geometry, or that it can be trained on one arbitrary geometry and operate on another arbitrary geometry? The latter seems much less likely so, worth emphasizing and explaining if it’s the case. And then, how different must the geometries be before results degrade?

To address your question: we mean that QNO can be trained on a wide range of station geometries and then successfully applied to unseen, arbitrary geometries at test time without retraining. This generalization ability arises from the fact that QNO, as a neural operator, learns a mapping between function spaces, where each input sample (i.e., the set of seismograms recorded at specific station locations) represents a distinct discretization of the same underlying function. In this view, station geometries serve as spatial sampling schemes, and QNO's prediction is conditioned on these variable sampling layouts.

To ensure the model learns from diverse geometries, we construct our training dataset such that each event is recorded by a different subset of 8–32 stations randomly selected from a larger network (see a collection of all stations on Figure 3). Additionally, we introduce further variability by injecting noise-only stations with randomized positions and real noise waveforms from STEAD into each training sample. This design exposes the model to a wide spectrum of station layouts during training, allowing it to generalize to new, unseen configurations during inference.

As for the question of how different the geometries can be before performance degrades: while we did not conduct a formal ablation study quantifying performance drop as a function of geometric dissimilarity, the real test dataset naturally contains highly diverse station layouts, including station combinations and spacing that are not explicitly seen during training. The consistent performance of QNO across this test set (see Figures 3 and 4) provides empirical evidence that the model is robust to substantial variations in geometry.

We have revised the manuscript accordingly. Please see the new subsection “*Generalization across Seismic Network Configurations*” which illustrate model behavior

under different network configurations (Lines 197–215 in the revised manuscript) and Supplementary Figs. 1 and 2 for input variability.

Secondary comments

1 “Microseismic Monitoring with Quake Neural Operator”→“Microseismic Monitoring with the Quake Neural Operator” to signify what you’re introducing.

Corrected.

5 “seismic phase picking” i.e., manually, or how? State in Abstract.

The text has been clarified: “*Traditional techniques rely heavily on manual or automated seismic phase picking*”.

12 “Our model” → “We show that our model”, if you show that.

Corrected to “We show that our model”

15 “over 3,000”, give the number.

A specific number is provided here. Note that we have added five additional days to this geothermal dataset, including an M4.5 mainshock, resulting in a richer collection of events.

15–16 “significantly surpassing”, state in the Abstract how we know the events are real.

The abstract has been revised: “*Approximately 59% of the detected events are also identified by PhaseNO, and manual inspection of tens of additional events confirms their seismic origin.*”

19 “industry applications” yes, but in the Abstract name a few that you mean.

The sentence has been revised: “*The broad application of QNO holds the potential to reveal previously undetected seismic activities, offering a more complete understanding of subsurface processes that are critical to both academic research and industry applications, including geothermal energy development, mining, and carbon capture and storage.*”

64–66 “as these approaches rely on the waveforms of detected events, they are not directly applicable to continuous data streams” is hard to understand. Including waveforms doesn’t imply discontinuities. Do you mean waveforms should be omitted simply to keep data streams smaller?

We agree that the original sentence (“*as these approaches rely on the waveforms of detected events…*”) may have caused confusion. Our intended meaning was not that the inclusion of waveforms implies discontinuity, but rather that these methods assume event waveforms have already been extracted from continuous recordings. To clarify this point, we have revised the sentence in the manuscript as follows (Lines 76–78 in the revised manuscript):

“*Furthermore, these approaches assume that the waveforms of seismic events have already been extracted from continuous recordings. As such, they are not designed to operate directly on raw continuous data streams where events have not yet been detected.*”

This change more accurately reflects the distinction between pre-segmented event waveforms and raw continuous data and addresses your concern.

93 “location” → “location and time”.

It has been corrected to “*earthquake location and origin time*”

106 “multiple stations with irregular geometries” → “multiple stations at irregularly distributed locations”, unless you mean each of the stations is weirdly shaped.

It has been corrected to “*multiple stations at irregularly distributed locations*”.

108–110 “Each input tensor contains six channels: three corresponding to the three-component waveforms and three corresponding to the station locations (x_i, y_i, z_i)” should be clearer. The locations share a single index i, so it seems none of x, y, z can vary independently from the other 2. Part of Fig. 1 suggests the input has the form $((f_{ji}(t) = f_j(x_i, y_i, z_i, t))_{j=1}^{3})_{i=1}^{n}$ that is a tensor of type 2 and shape $3 \times n$ for each t. It’s likely that you mean that location information is also input, that would imply a tensor $(f_{1i}(t), f_{2i}(t), f_{3i}(t), x_i, y_i, z_i)_{i=1}^{n}$ of type 2 and shape $6 \times n$ for each t, that is ambiguous in Fig. 1. Clarify.

Yes, we mean that, for each t, the input is a tensor of shape $6 \times n$, i.e., $(f_{1i}(t), f_{2i}(t), f_{3i}(t), x_i, y_i, z_i)_{i=1}^{n}$. The last three channels encode station coordinates and are constant in t: each channel repeats the corresponding coordinate (x_i, y_i, or z_i) along the time axis, so each coordinate channel has shape $1 \times n$ and is concatenated with the three waveform channels along the channel dimension to form the $6 \times n$ input. To make this explicit, we revised Figure 1 to indicate that the x_i, y_i, or z_i channels are time-invariant (e.g., traces are constant along the time axis), so readers can see that each coordinate channel remains constant over time.

111, 380 “15 seconds” at what sampling rate 1/Δt? Is it 100 Hz (l. 178)?

As a neural operator designed for functional mapping, the input function can be discretized at different sampling rates. This means that a well-trained neural operator can be applied directly to inputs sampled at rates different from those used during training. However, the physical domain size must remain fixed. This is why we specify the total input duration as 15 seconds, without mentioning the sampling rate at this point. In the numerical examples that follow, as you have pointed out, we provide the actual sampling rates used in the training datasets and the case study.

During revision, we have added a numerical example by upsampling all test dataset inputs from 1500 to 3000 time steps (corresponding to increasing the sampling rate from 100 Hz to 200 Hz), and directly evaluate the QNO model trained on the original 1500-point inputs. As shown in Supplementary Fig. 17 in the Supplementary Information, the model maintains strong performance under higher temporal resolution, highlighting its generalization capability at different temporal resolutions.

120 “noise” → “estimated noise”, at least the 1st time you introduce this. One never knows the true noise.

It has been corrected to “estimated noise”.

120–121 “mean across the station axis, consisting of four channels: $\hat{x}$, $\hat{y}$, $\hat{z}$, and $\hat{t}$ →“mean of four channels $\hat{x}_i$, $\hat{y}_i$, $\hat{z}_i$, and $\hat{t}_i$ across the station axis” (if correct), to avoid the trivial misinterpretation that the earthquake locations would be means of station locations; but it’s questionable if you mean that each station generates its own ($\hat{x}_i$, $\hat{y}_i$, $\hat{z}_i$, $\hat{t}_i$). If you don’t mean that, then it’s questionable if ($\hat{x}$, $\hat{y}$, $\hat{z}$, $\hat{t}$) is really a mean.

There is potential ambiguity in our original phrasing. Our intention was not to imply that the earthquake location is the mean of the physical station coordinates, but rather that each station produces a latent feature vector that contributes to the final prediction. Specifically, each station outputs a representation from which a location estimate is derived, and we apply mean pooling across the station dimension to aggregate these into a single prediction ($\hat{x}$, $\hat{y}$, $\hat{z}$, $\hat{t}$). To clarify this, we have revised the sentence to:

"The final location output ($\hat{x}$, $\hat{y}$, $\hat{z}$, $\hat{t}$) is computed by applying mean pooling across the station axis to the predicted location features."

We hope this resolves the ambiguity and makes the aggregation process clearer.

Fig. 1 Define FNO and GNO in the caption.

To define FNO and GNO, we have added the following sentence to the caption:

"The model integrates two basic neural operators: Fourier Neural Operators (FNOs) extract temporal features from individual station waveforms, while Graph Neural Operators (GNOs) capture spatial relationships across stations via a message-passing framework."

128 "in their best status" means what?

We agree that this wording was unclear. Our intended meaning was that each baseline model was evaluated using the checkpoint that achieved the best performance on the validation set during training. To clarify this, we have revised the sentence in the manuscript to:

Line 140-141 in the revised manuscript: *"In addition, each baseline model was evaluated using the checkpoint that achieved the best validation performance during training."*

We hope this revision resolves the ambiguity.

134 "multi-layer perception", did you mean "multi-layer perceptron"?

Thank you for pointing this out. This was a typographical error. We intended to refer to the standard neural network architecture "multi-layer perceptron" We have corrected the term accordingly.

137 “much accurate” → “much more accurate” (presumably).

This has been corrected to “more accurate” in the revised manuscript.

219 “similar high” → “similarly high”.

This has been corrected.

Table 1 Explain parenthetic numbers in caption.

We have added the following to the table caption: “Parenthetic values indicate the probability threshold used to classify a recording as containing an earthquake signal.”

We have revised the table caption to include the following clarification: “*Parenthetic values indicate the probability threshold used to classify a recording as containing an earthquake signal.*”

257 “multi-stations” → “multiple stations”.

This has been corrected.

Fig. 8 “PhaseNO did not find picks on many stations that contain earthquake signals classified by QNO”, make this a specific quantified statement.

We have revised the statement in the caption of Figure 8 to provide a specific quantified description. The updated sentence now reads:

“In the left and right panels, PhaseNO did not find picks on 10 and 8 stations, respectively, whose waveforms were classified by QNO as containing earthquake signals.”

This change clarifies the comparison by explicitly stating the number of stations involved in each case.

267 “deep-learning” → “the deep-learning”.

Corrected.

283 Is “multi-task” the same as “multi-objective”?

Thank you for your insightful question. We agree that multi-task learning and multi-objective learning are related, but they represent distinct concepts in machine learning.

Multi-task learning refers to training a single model to perform multiple related prediction tasks simultaneously by sharing representations across tasks. In our case, the model jointly learns (1) a classification task (predicting whether each station contains an earthquake signal) and (2) a regression task (predicting the earthquake's location and origin time). This is a standard multi-task learning setup.

Multi-objective learning, on the other hand, typically refers to optimizing multiple objectives that may be inherently conflicting, such as maximizing accuracy while minimizing model complexity, or balancing precision and recall. These objectives often require explicit trade-off management, such as Pareto optimization or scalarization techniques.

We acknowledge that multi-task learning can also involve implicit trade-offs between tasks, especially when their loss gradients compete. However, unlike multi-objective optimization, the goal in multi-task learning is to leverage shared structure across tasks to improve overall performance, rather than explicitly balancing competing objectives.

Therefore, we believe that "multi-task learning" is the appropriate term to describe our framework.

288 "time series" → "time coordinate".

Corrected.

291 "operator" → "operators".

Corrected.

295 For clarity, write "$\mathbf{p} = (\mathbf{p}_i)_{i=1}^{n} \in \mathbb{R}^{n\times 2}$" here, and ...

Corrected.

297 ... "$\mathbf{p}_i \in \mathbb{R}^2$" here.

Corrected.

306–307 “using Fourier transforms”, mention how you handle that the t-series is not periodic, so a jump from f (t_m) to f (t_1) would always imply some spurious fat-tail spectrum.

We agree that applying Fourier transforms to non-periodic time series can lead to spurious high-frequency components due to boundary discontinuities, resulting in a fat-tail spectrum. To mitigate this, we apply zero-padding of 50 points to both ends of the input time series. Furthermore, as noted in (Li et al., 2020a), high-frequency modes are more difficult to learn. Therefore, in each FNO layer, we retain only the first M lowest-frequency modes and truncate the rest. This spectral truncation effectively suppresses potential artifacts caused by non-periodicity. We have clarified this in the revised manuscript (Lines 427–432).

Reference:

Li, Zongyi, Nikola Kovachki, Kamyar Azizzadenesheli, Burigede Liu, Kaushik Bhattacharya, Andrew Stuart, and Anima Anandkumar. "Fourier neural operator for parametric partial differential equations." arXiv preprint arXiv:2010.08895 (2020).

320 “respectively” → “respectively for k = 3”, since it’s a new paragraph.

Corrected.

325 “Gaussian Error Linear Unit”, explain briefly.

We have added a brief explanation of the Gaussian Error Linear Unit (GELU) in the revised manuscript to clarify its role. Specifically, we now state that GELU is a smooth activation function that applies a probabilistic gating mechanism, approximating the input multiplied by the cumulative distribution function of a standard normal distribution (Hendrycks and Gimpel, 2016). Compared to standard ReLU, GELU improves gradient flow and has been shown to enhance performance in various deep learning applications. The revised sentence in the manuscript now reads:

“Nonlinearity is introduced in all FNO layers using the Gaussian Error Linear Unit (Hendrycks and Gimpel, 2016), which applies a smooth, probabilistic gating mechanism that approximates the input multiplied by the cumulative distribution function of a standard normal distribution.”

337 “we suggest” is very odd. You should just say what you did.

We agree that “We suggest selecting” should be corrected to “We select”. However, this paragraph was deleted during revision, and the relevant information is now presented on Lines 384-392 of the revised manuscript.

334 “and β” —There really is no reason to have 2 weights, correct?

You are correct that, mathematically, using both α and β introduces redundancy, since only the relative weighting between the two tasks matters. We have revised the manuscript to include only α in the loss function, which serve as a tunable hyperparameter to control the trade-off between classification and regression performance. We have revised the text accordingly for clarity (Lines 478-482).

353 “is the true values” → “contains the true values”.

Corrected.

386 “straight” → “straightforward”.

Corrected.

388–389 “we typically use” —Say precisely what you did for this work.

“we typically use” has been corrected to “we use”.

424 “Adaptive Moment Estimation method” needs a citation and a brief summary here.

We have revised the sentence to include the citation (Kingma and Ba, 2014) and a brief description of the Adam optimizer. Specifically, we now state that Adam is a gradient-based optimization method that adaptively adjusts learning rates using estimates of first and second moments of the gradients. The revised sentence has been included in the manuscript (Lines 567-568).

Reference:

Kingma, D.P. and Ba, J., 2014. Adam: A method for stochastic optimization. arXiv preprint arXiv:1412.6980.

425 “$10e^{-4}$” → “10^{-4}” (unless you really do mean 10 exp(−4)) (and similarly if such a typo recurs).

It has been corrected to “10^{-4}”.

We thank the reviewer again for the thoughtful review and constructive comments that helped improve the clarity and rigor of the manuscript.

Reviewer #2

Reviewer #2 (Remarks on code availability):

Review of "Microseismic Monitoring with Quake Neural Operator"

This manuscript introduces the Quake Neural Operator (QNO) for microseismic monitoring, aiming to detect and locate earthquakes directly from continuous seismic data without explicit phase picking. While the idea has merit, the manuscript suffers from several critical shortcomings that significantly undermine its novelty and impact.
The QNO's architecture, which combines Fourier Neural Operators (FNO) for temporal data and Graph Neural Operators (GNO) for spatial data, closely mirrors that of Phase Neural Operator (PhaseNO)(Sun, et. al., 2023. Phase neural operator for multi-station picking of seismic arrivals. Geophysical Research Letters, 50, e2023GL106434. https://doi. org/10.1029/2023GL106434).

We appreciate the concern and clarify that, although both QNO and PhaseNO leverage operator-learning components (FNO/GNO), their rationale, task, outputs and empirical behavior differ substantially.

(1) Problem and rationale:

- PhaseNO is a per-station phase picker (P/S arrivals). Pickers can struggle on very low-SNR or emergent onsets where clear phase onsets are unavailable (see Figure 5 and Supplementary Fig. 7-16 on various examples).
- QNO performs end-to-end event detection and location directly from continuous multi-station, waveforms without phase picking, estimating both the presence of an event on each station and its location $(\hat{x}, \hat{y}, \hat{z}, \hat{t})$ in a single unified model.

(2) Learning objectives:

- PhaseNO is a single-task model supervised by phase labels.
- QNO is multi-task, combining classification (probabilities of signal or noise at each input station) and regression of event-level information $\hat{x}, \hat{y}, \hat{z}, \hat{t}$ of all input stations) with a joint loss; no phase labels are required.

(3) Architecture difference:

- PhaseNO uses a U-shaped architecture where input and output share the same temporal sampling.
- QNO uses a simpler architecture with a graph-level feature extractor that aggregates information across stations to produce event-level predictions and

supports arbitrary network geometries. QNO yields robust performance at 0 dB SNR where PhaseNO fails (see Figure 5; Supplementary Figs. 7–16).

(4) Performance:

- Across the reported experiments, QNO shows robust detection at low SNR and accurate localization, addressing key failure modes of pick-based pipelines. Comparative results with PhaseNO are provided on the NCEDC test set across Northern California and the Geysers geothermal field; QNO outperforms PhaseNO across the tested settings (see Figure 5; Supplementary Figs. 7–16; Figures 6-8; Supplementary Figs. 20-28).

While these distinctions were noted in the original submission, the revision further clarifies and strengthens QNO's contributions; we clarify how QNO differs from PhaseNO in the discussion section (Lines 343-354); a summary of major changes appears at the start of this response. In particular, QNO's simpler architecture yields much lower computational cost, which we quantify in the revised manuscript, enabling real-time monitoring (see Results subsection "Computational cost of QNO" on Lines 291–319 and Figure 9).

Here are some major concerns:

Incremental advancement: The QNO builds upon existing methodologies, particularly the PhaseNO, with only incremental improvements. The core concept of using neural operators for seismic event detection and location is already established. The manuscript fails to clearly articulate how QNO substantially differs from PhaseNO beyond parameter tuning and slight architectural adjustments.

We respectfully disagree. QNO is not a parameter tweak of PhaseNO; it reframes the task (event detection + localization, not picking) and supervision (multi-task, no phase labels). To avoid redundancy with our reply above, we briefly recap the substantive differences and new evidence:

(1) The conceptual difference between PhaseNO and QNO is fundamental: PhaseNO performs per-station P/S picking; QNO performs end-to-end detection + location from continuous multi-station data without picks.

(2) PhaseNO is single-task using phase labels; QNO is multi-task (station-level classification + event level regression) with a joint loss, requiring no phase labels.

(3) QNO provides robust low-SNR detection (incl. 0 dB) and accurate location, with consistent gains over PhaseNO on NCEDC (Northern California) and the Geysers sequence. We also report computational cost demonstrating near-real-time feasibility of QNO (see Lines 291–319 and Figure 9).

Lack of novelty: The manuscript introduces a multi-task learning algorithm called the Quake Neural Operator (QNO), designed to directly detect and locate earthquakes from continuous data recorded by multiple stations in a seismic network with arbitrary geometry. However, the use of multi-task learning, combining classification and regression tasks, is not a novel concept. Such approaches are common in machine learning and have been previously explored in seismology. The manuscript does not provide sufficient evidence to demonstrate a significant advantage of this specific implementation over existing methods.

While multi-task learning is common in machine learning, its use with neural operators for direct detection and location of earthquakes is, to our knowledge, new in seismology and geophysics. The primary motivation of this work is to overcome a key limitation of picking based seismic monitoring workflows, whose performance degrades on low-SNR microseismic signals with indistinct onsets (see Figure 5; Supplementary Figs. 7-16 with the evaluation of PhaseNO at SNRs of 20, 10, and 0 dB). Furthermore, without picking to estimate earthquake locations in conventional travel-time based methods, QNO predicts the locations of detected events directly from input multi-station full-waveform data. Learning detection and location jointly enables us to build earthquake catalogs directly from continuous data. This also address a key limitation of full-waveform earthquake location algorithms (whether AI-based or classical) which typically operate only after an event has been detected and are not designed to run effectively on continuous streams.

Moreover, the manuscript provides extensive comparisons with baselines (PhaseNO and SeismicGNN) to demonstrate QNO's performances. If there are prior works that simultaneously detect and locate events directly from continuous multi-station data without picks, using operator learning on dynamic irregular seismic networks, we would appreciate the specific citations and will discuss them explicitly.

Limited Scope: The evaluation of QNO is primarily focused on the Geysers geothermal field. While this is a relevant application, the manuscript does not provide sufficient evidence to demonstrate the broad applicability of QNO to other regions or seismic settings. The specific geological and noise characteristics of the Geysers might favor QNO, and its performance may not generalize well to areas with different seismic signatures or network configurations.

We evaluated QNO in two settings: (1) the NCEDC test set across Northern California under a temporal split (train on past, test on future, see Figures 3-5; Supplementary Figs.

4-17), and (2) the Geysers geothermal dataset. In this revision, we include an M4.5 mainshock and its aftershocks, demonstrating applicability to a broader range of signals beyond the microseismicity used for training (M < 2; see Figs. 6–8 and Supplementary Figs. 18–28).

We further evaluated generalization across network configurations and temporal resolution, showing that the performance QNO remains stable under variations in station number, station geometry, and sampling rate of input seismic data. Please see the subsection "*Generalization across Seismic Network Configurations*" in the Results section (Lines 197-215 and the associated figures) of the revised manuscript.

Overstated claims: The abstract claims that QNO can reveal previously undetected seismic activities. However, the results presented only show a higher detection rate compared to routine catalogs and PhaseNO + GaMMA. There is no independent validation to confirm that these additional detections are indeed real events and not false positives.

We report QNO results under two station-level probability thresholds for confirming a signal (0.8 and 0.6). Both QNO (0.8) and QNO (0.6) catalogs show temporal patterns that closely track the PhaseNO-based enhanced catalog as an independent reference, across the 8-hour histograms in Figure 6a and the hourly detection rates in Supplementary Fig. 18, including the aftershock-rich period following the M4.5 mainshock on 9 June 2024. Because PhaseNO has been benchmarked against PhaseNet, EQTransformer, and EdgePhase (Sun et al., 2023), agreement with the PhaseNO-based catalog provides strong supporting evidence that QNO advances catalog construction beyond picking-based workflows.

We also compare event locations across four catalogs, QNO (0.8), QNO (0.6), PhaseNO, and NCEDC, and find good consistency (Figure 6b), further supporting that most additional QNO detections are genuine seismic events.

Finally, we manually reviewed a substantial number of new detections and include representative examples (Figures 7 and 8; Supplementary Figs. 20–28). These illustrate why PhaseNO + GaMMA missed them: picks are present at only a few stations and absent at many noisy stations, so the phase-association step, which requires a minimum number of picks, filters these events out.

Unconvincing benchmark: The comparison with PhaseNO is not entirely convincing. The manuscript mentions that QNO applies stricter criteria than PhaseNO, which could explain

the higher number of detected events. Without a more detailed analysis of the trade-off between precision and recall, it is difficult to assess the true improvement offered by QNO.

Precision and recall trade-offs are analyzed in Table 1 and discussed in the section "Application to Microseismic Monitoring at the Geysers Geothermal Field" (lines 264–274). We report precision and recall for QNO at two station-level probability thresholds for confirming a signal (0.8 and 0.6). The NCEDC routine catalog has been updated since our initial download and now includes 874 events, we have updated the quantitative analysis of F1 score, precision, and recall in Table 1 accordingly.

Additionally, we clarify that QNO identifies more events than PhaseNO even under a stricter detection criterion; this observation is not intended as an explanation for the higher detection rate.

Scalability and computational cost: The manuscript acknowledges that the computational cost of QNO scales quadratically with the number of stations. This is a significant limitation, especially for large seismic networks. The suggested solution of selecting a subset of stations may compromise the detection performance and undermines the advantage of using network-wide information.

This appears to be a misunderstanding. The manuscript does not make that claim, and QNO does not scale quadratically in practice. The comment likely conflates our approach with the limitation discussed in PhaseNO (Sun et al., 2023), where a fully connected pairwise graph over all stations indeed leads to quadratic cost. The idea of selecting a subset of stations is also discussed in the PhaseNO paper, not in our QNO manuscript.

In QNO, we do not use a fully connected graph. Instead, we construct the graph in the GNO layer based on a geographic distance threshold D (see Supplementary Fig. 3). Each node represents a seismic station, and edges are established between pairs of stations whose geographic distance is less than or equal to D. Self-loops (edges from a node to itself) are included. The computational cost is largely affected by the number of edges in the graph (see Figure 9), which is controlled by the relative distance between the distance among stations and the distance threshold D. For the same graph, increasing D increases the number of edges, thereby enlarging the neighborhood of each node and raising the computational cost due to additional node-to-node communications. For example, the 10 stations in the left panel of Supplementary Fig. 3 become fully connected (i.e., form a complete graph) when D is set to 60 km. In contrast, when a large number of stations are present, as in the right panel, a relatively small D can be chosen to reduce computational

cost while still preserving effective communication among stations, as evidenced by generalization when training with D =40 km and testing with D =20 km and D=60 km (see Supplementary Fig. 29. These results indicate QNO is practical for real-time monitoring under typical regional networks.

We have added a subsection, "Computational cost of QNO," to the Result and quantitatively evaluate the runtime and memory under different seismic network configurations; please see Lines 291-319 and Figure 9 in the revised manuscript.

Recommendation
I recommend rejecting the manuscript in its current form. The authors need to:

As the comments below restate earlier points, we summarize our responses briefly to avoid redundancy.

Clearly articulate the novel contributions of QNO and provide a more thorough comparison with existing methods, especially PhaseNO.

We clarified how QNO differs from PhaseNO in the discussion section (Lines 343-354). We gave a thorough comparison with PhaseNO on both NCEDC Northern California dataset and the Geysers geothermal datasets (see Results).

Provide more robust validation of the detected events, using independent datasets or techniques.

We have provided cross-validation of four catalogs: QNO (0.8), QNO (0.6), PhaseNO, and NCEDC catalogs for the Geysers geothermal datasets. PhaseNO and NCEDC catalog both serve as comparison with independent techniques. Waveforms are independent dataset as they were never seen by QNO during training.

Address the limitations of QNO, including its computational cost and potential for overfitting to specific seismic settings.

QNO does not have the computational issue, and the computational cost is low (Lines 291-319 and Figure 9). We also show the stable generalization of QNO across seismic network configurations. Please see "*Generalization across Seismic Network Configurations*" on Lines 197-215.

Demonstrate the generalizability of QNO to other regions and seismic networks.

We evaluated the performance of the same trained QNO on both NCEDC (Northern California) and Geysers; We also show the stable generalization of QNO across seismic network configurations. Please see "*Generalization across Seismic Network Configurations*" on Lines 197-215.

Without substantial revisions that address these concerns, the manuscript does not offer sufficient scientific advancement to warrant publication.

We thank the reviewer again for reviewing our manuscript and comments that helped improve the clarity of the manuscript.

Reviewer #3

Reviewer #3 (Remarks to the Author):

The manuscript presents a novel deep learning framework, the Quake Neural Operator (QNO), for direct detection and location of microseismic events from continuous waveform data, bypassing the traditional reliance on seismic phase picking. The method integrates Fourier Neural Operators for temporal encoding and Graph Neural Operators for spatial relationships across stations, enabling it to work with arbitrary seismic network geometries. The multi-task learning framework jointly solves detection (classification) and location (regression), which improves performance by leveraging shared representations. The model is benchmarked against state-of-the-art approaches (e.g., PhaseNO, SeismicGNN) and shows superior performance, particularly under noisy conditions. The application to The Geysers geothermal field demonstrates QNO's capability to significantly expand earthquake catalogs beyond what existing methods provide.

We thank Reviewer #3 for recognizing the novelty of the proposed method and the contribution of this work. We appreciate the constructive suggestions for improving the manuscript. In response, we have made substantial revisions throughout the manuscript. Detailed responses to each comment are provided below.

Comments:

1. The QNO architecture is highly original in the field of microseismic monitoring. While the novelty is clear, the authors could strengthen the discussion around the limitations of operator learning in handling complex or overlapping seismic events, especially when dealing with thousands of events as in the dataset mentioned.

During revision, we extended the analysis window by five days (2024-06-08 00:00 to 2024-06-13 00:00) to include the M_w 4.5 mainshock on 2024-06-08 19:34:29, one of the largest earthquakes in the Geysers region in the past decade, to evaluate QNO performance during the aftershock-rich period. The revised dataset now spans 10 days (2024-06-08 00:00 to 2024-06-18 00:00, see Supplementary Fig. 18). Using PhaseNO as the external benchmark, we constructed a deep-learning–enhanced catalog and analyzed how many overlapping events occurring within 10 s may have been missed by the QNO model trained with a 15 s time window and applied with a 5 s overlap.

Supplementary Fig. 30 shows the distribution of inter-event times in the PhaseNO catalog. Among the 5,444 events, 94.64% have inter-event times greater than 10 s, which are readily detected by QNO. The highest seismicity rate occurred immediately after the M_w 4.5 mainshock. Of the 292 events (5.36%) with inter-event times less than 10 s, most occurred within six hours of the mainshock, which highlights the periods that are especially challenging for a QNO trained with a 15 s window and 5 s overlap.

We quantitatively evaluated QNO performance during this aftershock-rich period by plotting hourly earthquake occurrence rates (Supplementary Fig. 18). In the hour of the mainshock, QNO with a threshold of 0.8 detected 291 events, compared with 179 events detected by PhaseNO in the same period. While QNO may have missed some closely spaced overlapping events, it still achieved a higher detection rate, likely due to its ability to identify low-SNR and low-magnitude events embedded in continuous waveforms.

We have incorporated these results into the Discussion section of the main text (lines 355-369 and 384-394). Further analysis of how QNO handles overlapping events within the same 15 s time window is provided in the response to the next comment.

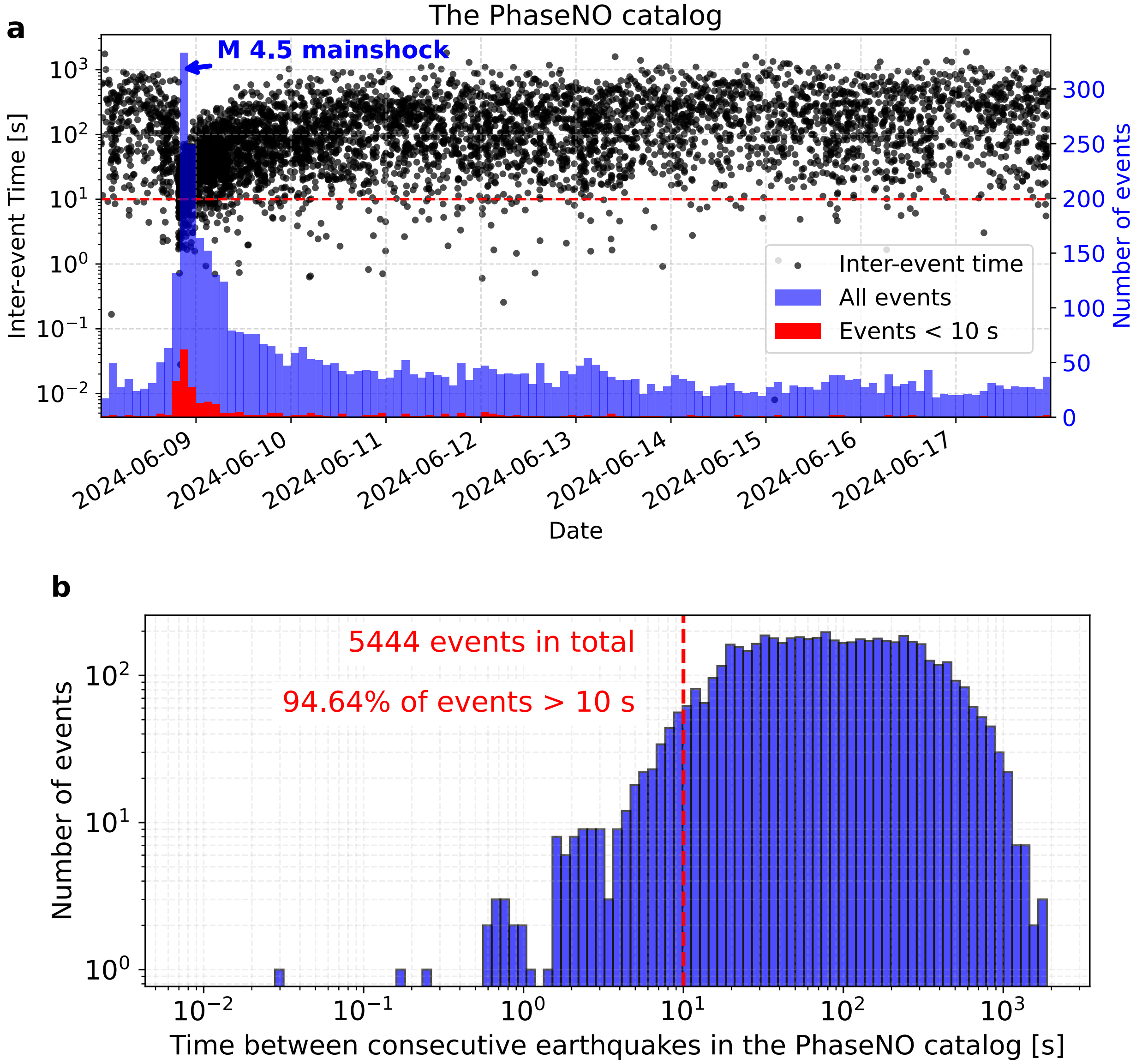

Supplementary Fig. 30: ***Time intervals between consecutive earthquakes (inter-event times) in the Geysers geothermal catalog constructed using PhaseNO***. ***a***. *Inter-event time of the catalog plotted against origin time (left vertical axis), overlaid with histograms of total event counts and counts of events with inter-event times shorter than 10 s (right vertical axis) computed in 2-hour bins. The horizontal dashed red line marks the 10 s threshold. The M 4.5 mainshock occurred at 19:34:29 UTC on 8 June 2024.* ***b***. *Log–log histogram of inter-event times. The vertical dashed red line marks the 10~s threshold used to identify closely spaced events, which are challenging for QNO models trained with a 15 s time window and applied with a 5 s overlap.*

2. The method assumes one event per 15-second window (P6, P21), which may limit applicability in aftershock-rich periods or during intense hydrofrac phase. While overlapping windows are proposed as a workaround, more discussion is needed on its limitations and potential alternatives.
Include more detail on how QNO performs with multiple closely spaced events, e.g. in the Supporting Information.

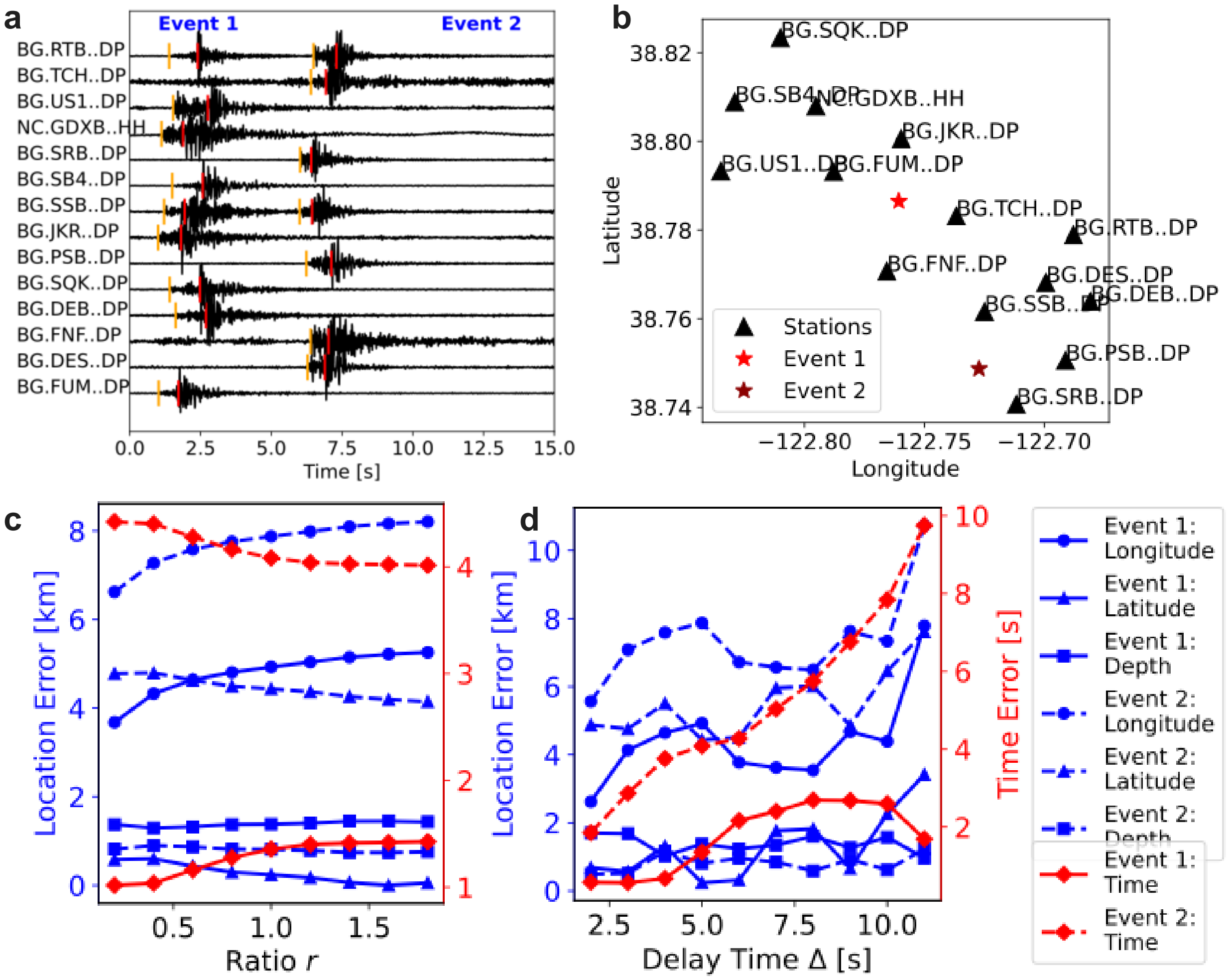

Supplementary Fig. 31: ***Performance of QNO for two example events from the test dataset within a 15 s time window, under varying amplitude ratios and delay times**. **a** Example of stacked waveforms with a 5 s delay between the two events and an amplitude ratio of 1. Manually picked P-phase (orange) and S-phase (red) arrival times are marked on the waveforms for reference. **b** Map showing all station locations and catalog locations of the two events. **c** Errors between QNO predictions and catalog locations for varying amplitude ratios with a fixed delay time of 5 s. **d** Errors between QNO predictions and catalog locations for varying delay times with a fixed amplitude ratio of 1.*

To address this comment in the revision, we manually stacked two events from the NCEDC test dataset, controlling their amplitude ratio and time interval. Not all input stations recorded clear waveforms of both events with manually picked phases, but QNO still estimated signal probabilities for all stations and located a single event within the input 15 s window. Regardless of whether the input contained one or two events, QNO accurately distinguished earthquake signals from noise; for this example, it output probabilities near 1 for all stations. The key questions then are: (1) which event's catalog location is closest to QNO's predicted location, and (2) how the presence of one event affects the location of the other.

We quantify this by controlling the amplitude ratio r and delay time Δ between two events:

$$s(t) = A_1 w_1(t) + rA_1 w_2(t - \Delta),$$

where $s(t)$ is the superposed signal, the first event has amplitude A_1 and normalized waveform $w_1(t)$, and the second event is a delayed, amplitude-scaled version of $w_2(t)$ with amplitude rA_1 and delay Δ seconds. Adjusting r and Δ allows us to control their relative strength and arrival time difference. The delay time between two events is defined as the difference between the earliest P-wave arrival times, measured across all stations that recorded each respective event.

Supplementary Fig. 31 shows the location errors of QNO for two example events from the test dataset within a 15 s time window, under varying amplitude ratios and delay times. The location and origin time predicted by QNO are compared with the catalog values for both Event 1 and Event 2 to evaluate which event is actually located by QNO when it assumes only one event is present in the input time window. In this example, the location error for Event 1 is clearly smaller than that for Event 2, indicating that QNO tends to locate the earlier-occurring event. Moreover, Event 2 has a larger azimuthal gap than Event 1, which may also contribute to its larger location errors. For both events, the longitude location error is noticeably larger than the depth, latitude, and origin time errors, likely because the range of station longitudes is small, resulting in insufficient horizontal constraints for longitude estimation.

We examined the location errors as the amplitude ratio r increased from 0.2 to 2. In the stacked waveforms, Event 2's signals are most prominent when $r = 2$, while Event 1's signals are most prominent when $r = 0.2$. The variation in origin time error reflects this pattern well, whereas Event 1's location results remain relatively unchanged. Longitude errors for both events do not strongly reflect this amplitude-ratio dependence, again likely due to insufficient horizontal station coverage.

For delay time Δ, a larger Δ results in Event 2 occupying a shorter portion of the 15 s time window. In such cases, QNO's location errors with respect to Event 2's catalog longitude, latitude, depth, and origin time increase, whereas Event 1's location results remain relatively stable. These changes are not strictly linear or monotonic, which may be due to waveform complexity, the spatial distribution of seismic stations, and the fact that not all stations recorded both events. Nevertheless, we can broadly estimate the trends and possible causes of the errors.

Due to the diversity of waveform and station characteristics, the detailed variation patterns for individual events may differ; however, the overall trend is roughly consistent: Event 1's location errors are consistently smaller than those of Event 2 and are less sensitive to changes in amplitude ratio and delay time. This indicates that when multiple events occur within the input window, QNO probably locates the first event, while the second event is often overlooked by QNO's detection component.

It is important to emphasize that, although all numerical examples in this study use a 15 s waveform length as input, this length is not fixed. By adjusting the parameter in QNO that controls the input layer size, we can use waveforms of any length. However, for a trained QNO model, the input signal length during application must match the length used during training (the input sampling rate, however, does not need to be the same). Therefore, it is entirely feasible to train multiple QNO models for different input lengths. For example, during periods of extremely high seismicity, such as in the very early aftershock stage following a large mainshock (e.g., within the first hour), a QNO model trained with a shorter time window could be used. In this stage, earthquake signals are generally clear enough for PhaseNO to pick arrival times, so it is not always necessary to use QNO, which is better suited for detecting low-SNR microseismic signals without phase picking.

In practical applications, we can also combine QNO with PhaseNO to estimate the inter-event times of an enhanced earthquake catalog, thereby determining an suitable input time-window length. This approach balances minimizing missed events with maximizing detection performance. Given QNO's improved detection rate, especially for microseismicity, we consider a small number of missed closely occurring events acceptable. For instance, in the Geysers geothermal dataset examined in this study, only 5.36% of events in the PhaseNO catalog have origin times less than 10 s after the previous event.

We have added this discussion to the Supplementary Information (Supplementary Note 3: Multi-event superposition test for QNO) and to the Discussion section (lines 370–383) of the revised manuscript.

3. Extensive benchmarks against SeismicGNN and PhaseNO show QNO's superiority, especially in low SNR scenarios. The ground-truth catalog from NCEDC is used as a reference, but with limited completeness (only 45 events in 5 days vs. > 3000 detected by QNO). This makes true precision/recall metrics ambiguous.
I would suggest to add a more detailed error analysis of false positives and false negatives, possibly via manual inspection or alignment with external catalogs, to validate the increase in detection rate.

When we initially downloaded the NCEDC catalog in August 2024, only about two months had passed since the study period, which may explain why the catalog contained only 45 events at that moment. Considering that the NCEDC catalog might have been updated in the past few months, we re-downloaded it during the revision stage. We found that for the original study period (2024-06-13 00:00 to 2024-06-18 00:00), the updated NCEDC catalog contains 280 events, making it more complete than the version we originally used.

In addition, we extended the analysis window by five days to include the M_w 4.5 mainshock on 2024-06-08 19:34:29, one of the largest earthquakes in the Geysers region in the past decade, in order to study QNO performance during the aftershock-rich period. The revised dataset now spans 10 days (2024-06-08 00:00 to 2024-06-18 00:00) and contains 874 events in the updated NCEDC catalog. We updated the Geysers case study results accordingly, including the recall and precision values in Table 1.

We could not find any publicly available enhanced earthquake catalog for the Geysers geothermal dataset in the period, so we constructed our own enhanced catalog using PhaseNO as the external benchmark. Importantly, both PhaseNO and QNO used the same stations and the same waveforms, ensuring a fair comparison between the two methods. We visually inspected many of the events newly detected by QNO (see Figures 7 and 8 in the main text, Supplementary Figs. 20–28 and Supplementary Note 2 *Additional examples comparing QNO and PhaseNO* in the Supplementary Information). For most of these newly events detected by QNO, PhaseNO had picked phases on some stations; however, the probability functions predicted by PhaseNO were relatively low in amplitude and broad in width, reflecting the low SNR and the difficulty of picking arrivals with unclear onsets for these low-magnitude events. Moreover, these events often had fewer than the minimum 16 picks per event required for association in the PhaseNO catalog, and thus were

excluded during phase association process. In contrast, QNO identified signals on many more stations than PhaseNO, allowing it to detect a substantially greater number of events.

4. Whereas the Geysers case study is compelling and demonstrates the model's utility in geothermal monitoring, consider discussing how QNO could support real-time monitoring pipelines, especially in industrial applications (e.g., geothermal energy or carbon sequestration)

We have added a subsection titled "*Computational Cost of QNO*" in the Results section (see Lines 291-319 and the newly added Figure 9 in the revised manuscript), where we discuss the computational cost and evaluate the capability of QNO for real-time monitoring.

We investigate the capability of using QNO for real-time monitoring. Although training QNO is time consuming, once the model is well trained it can be directly applied to monitor the area of interest. Figure 9 shows the computational cost of one prediction during testing, evaluated on an Apple M3 Max with results averaged over six runs. The computational cost is reported for different numbers of stations and graph configurations using only CPU with two threads. The computational time and memory usage largely depend on the number of edges and stations in a single input sample. For example, a seismic network with 20 stations and 400 edges (fully connected) in the graph requires approximately 1 s per 15 s input sample and about 2.4 GB of memory. This represents the maximum computational cost for the 20-station case, which can be reduced by decreasing the number of edges. Even with 50 stations, the maximum computational cost is 5.5 s per 15 s input sample with a memory usage of about 5.5 GB. For a real-time data stream where the system waits 10 s before processing the next prediction (with 5 s overlap between two predictions), this 5.5 s computational time allows QNO to be deployed directly for real-time monitoring using CPU with two threads.

For a given seismic network with a fixed number of stations and geometry, the number of edges is determined by the graph configuration in the GNO layer, which is constructed based on the physical distance between stations (see Supplementary Note 1 and Supplementary Fig. 3). For a given station, edges are created between that station and any other station (including itself) if the distance between the two is less than or equal to a physical distance threshold D. A D value larger than the maximum distance among stations results in a fully connected graph with a total of n^2 edges, which requires the highest computational cost. In practice, for large networks one can choose a relatively

small D to reduce computational cost while preserving effective inter-station communication, as evidenced by generalization when training with D=40 km and testing with D=20 km and D=60 km (Supplementary Fig. 29). For the 50-station network evaluated in Figure 9, the computational cost decreases substantially when the number of edges is reduced, which further enables the deployment of a trained QNO model for real-time monitoring.

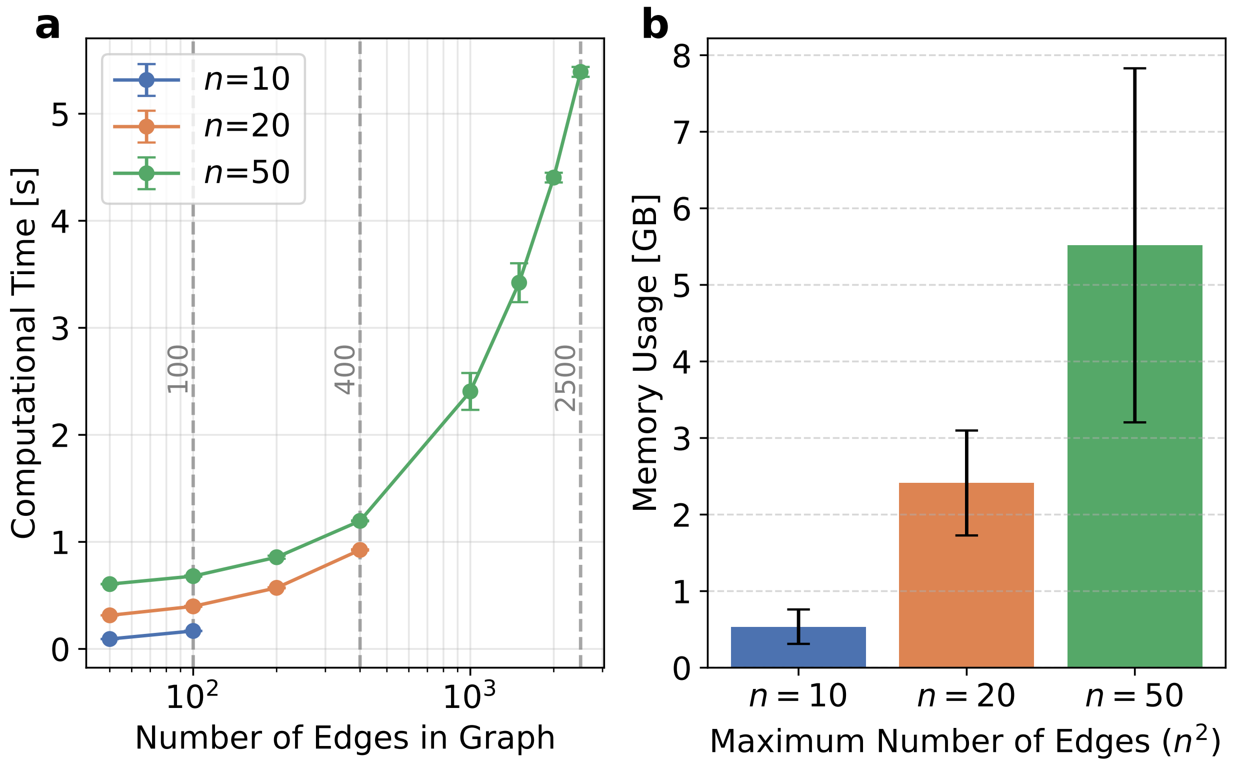

Figure 9: ***Computational cost of one prediction during testing, evaluated on an Apple M3 Max using CPU only with two threads, with results averaged over six runs*. *a*** *Computational time (mean±standard deviation) as a function of the number of edges in the graph for different numbers of stations n. Each curve corresponds to a fixed n, and dashed vertical lines indicate the maximum possible number of edges n^2 for that configuration.* ***b*** *Memory usage (mean±standard deviation) for graphs at the maximum number of edges n^2 for n = 10, 20, and 50, reported in gigabytes.*

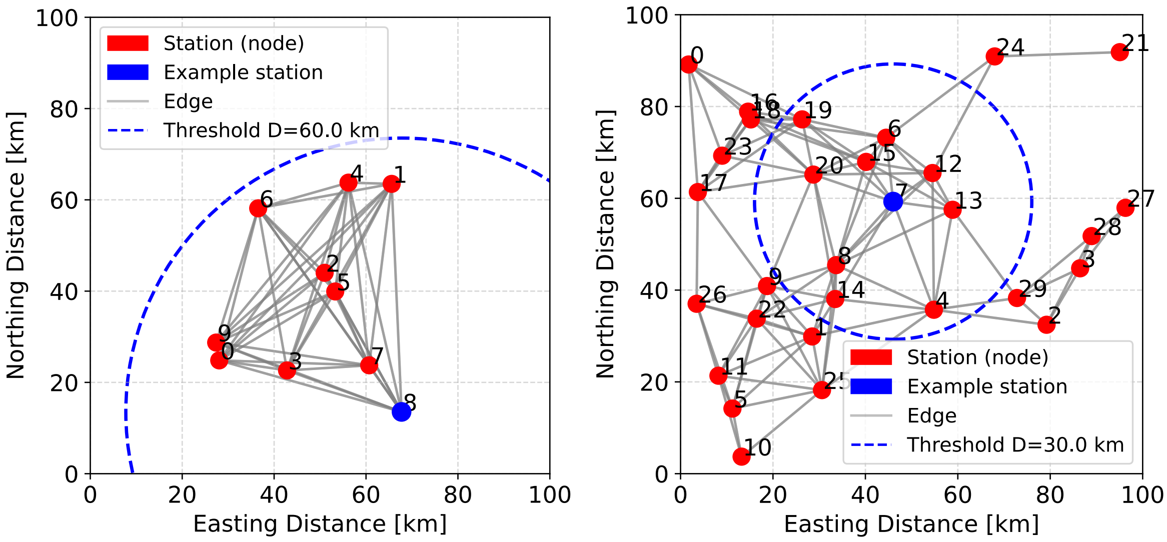

Supplementary Fig. 3: ***Illustration of graph construction in the GNO layer based on a geographic distance threshold D.*** *Each node represents a seismic station, and edges are established between pairs of stations whose geographic distance is less than or equal to D. Self-loops (edges from a node to itself) are included. The computational cost is largely affected by the number of edges in the graph (Figure 9), which is controlled by the relative distance between the distance among stations and the distance threshold D. For the same graph, increasing D increases the number of edges, thereby enlarging the neighborhood of each node and raising the computational cost due to additional node-to-node communications. For example, the 10 stations in the left panel become fully connected when D is set to 60 km. In contrast, when a large number of stations are present, as in the right panel, a relatively small D can be chosen to reduce computational cost while still preserving effective communication among stations* as evidenced by generalization when training with D=40 km and testing with D=20 km and D=60 km (Supplementary Fig. 29).

5. The manuscript is well-structured and rich in illustrations, however, figure legends (e.g., Figs. 4–9) could be more concise. Some are very long and takes whole paragraphs.

We simplified the figure legends during the revision. However, our aim was to ensure that readers can interpret the figures without having to refer exclusively to the main text. While some legends remain longer than usual, they fully comply with the journal's requirements (https://www.nature.com/documents/ncomms-submission-guide.pdf) which state:

"Figure legends should be <350 words each. They should begin with a brief title sentence for the whole figure and continue with a short statement of what is depicted in the figure, not the results (or data) of the experiment or the methods used. Legends should be detailed enough so that each figure and caption can, as far as possible, be understood in isolation from the main text."

We believe our current legends achieve this goal by providing sufficient standalone context for the reader.

Reviewer #3 (Remarks on code availability):

The availability of code and pretrained models upon publication is essential for reproducibility and community adoption.

The QNO software will be archived on GitHub and Zenodo if the manuscript is accepted; links will be provided in the “Code availability” section at publication.

We sincerely thank Reviewer #3 once again for their thoughtful review and constructive feedback, which have been very helpful in improving the quality and clarity of our manuscript.

Response to Reviewers' Comments

On Manuscript NCOMMS-25-07335A

Table of Contents

Reviewer #1 .... *1*
Reviewer #2 .... *8*
Reviewer #3 .... *15*

Reviewer #1

This work introduces a new algorithm to build earthquake catalogs from seismic data without phase picking. Creating such catalogs is crucial to the study of natural earthquakes, but also and especially for monitoring of seismicity induced by geothermal energy development, mining, carbon capture and storage etc. The authors' replies to my last report are mostly acceptable, with exceptions noted below. It is very important not to assert a result as if it were established rigorously mathematically vs merely supported empirically.

We thank the reviewer for the thorough evaluation and constructive feedback. We appreciate the recognition of the method's potential as well as the comments aimed at improving clarity and presentation. We have carefully revised the manuscript in response to these suggestions, and we address each primary and secondary point in detail below.

Primary comments

Six citations are to arXiv manuscripts, or lack publication details. Is that acceptable to the journal? Can they be replaced by citations to refereed work?

All citations to arXiv manuscripts have now been replaced with references to peer-reviewed publications wherever possible. The only exceptions are the following two works, for which no refereed versions exist and only arXiv preprints are available. Both papers are widely used and highly cited in the machine learning community (with approximately 230,458 and 9,313 citations on Google Scholar, respectively):

- Kingma, D. P., and J. Ba, 2014, Adam: A method for stochastic optimization: arXiv preprint arXiv:1412.6980.
- Hendrycks, D., and K. Gimpel, 2016, Gaussian Error Linear Units (GELUs): arXiv preprint arXiv:1606.08415.

45–47 My "extent the DL approaches are quantitatively more accurate" was addressed. Not all readers know if "0.05 s" is that small so, report MAE divided by the picked times.

In seismic phase picking, MAE is typically reported in absolute units because arrival times vary significantly across events and stations, making normalization by arrival time non-standard. To give readers a clearer sense of scale, we have added the following clarification in the manuscript:

"With a sampling rate of 100 Hz, which is commonly used for regional seismic networks, an error of 0.05 s corresponds to only five samples, which is very small relative to the total waveform duration."

This additional context helps readers appreciate the quantitative accuracy of the picks without introducing non-standard normalized metrics.

93 To my "neural operator architectures" authors replied with "we have added a review section . . . at the beginning of the Supplementary Information". So cite that.

We now cite the corresponding section in the Supplementary Information in the main text: "Furthermore, by leveraging neural operator architectures (see Supplementary Note 1), QNO adapts effectively to various seismic network geometries."

97–137 Authors disagree with my 'none of this belongs in "Results"'. While I agree with their "design of QNO . . . constitutes a key outcome", reading 40 ll. about methodology before results really saps exposition momentum. Push all but the 1st sentence to Methods.

Considering the reviewer's suggestion regarding the writing and structure of the manuscript, we have substantially shortened the description of QNO in the Results section. In particular, we removed the detailed description of the FNO and GNO components and their computational roles. We now retain only a brief introductory paragraph and a high-level summary of the architecture to provide sufficient context for

interpreting the subsequent analyses. Full technical details are now contained only in the Methods section.

We would also like to note that, in methods-focused contributions published in this journal, it is common practice to include a concise summary of the method at the beginning of the Results section to contextualize the experiments. For example, the EQTransformer paper (Mousavi et al., 2020) follows this structure.

106–108 Authors reply to my '"QNO . . . on varying grids without . . . model retraining" . . . seems unlikely' with helpful empirical results, but also by baldly asserting "QNO learns a resolution invariant mapping between function spaces", which is just another kind of very strong i.e., dubious claim.
(a) They refer to the Supplementary, but there is only one mention of "resolution" (just in the temporal sense) and no mentions of "invariant" in the Supplementary.
(b) The Supplementary (but not the main text) cites Kovachki et al., who define discretization invariant in terms of a discrete refinement $(D_n)_{n=1}^{\infty}$ of the problem domain D, requiring that $\forall \epsilon > 0\ \exists L \in N$ such that $D \subseteq$ the union of all radius-ϵ balls centered on all $x \in D_L$. This requirement is key in the op. cit. Appendix E proof of Theorem 8, but is not fulfilled by the spatial part of the authors' data domain.
(c) Furthermore, the op. cit. Definition 4 defines invariance as the risk vanishing only in the infinite sample-count L limit. For $L < \infty$ and non-fulfilling D_n as in the authors' case, resolution invariance is not guaranteed.
(d) Figures 3, 4 compare QNO to NCEDC and SeismicGNN estimates, and show that QNO can handle spatially irregularly sampled data, which are nice results, but do not really illustrate resolution-invariance.
(e) "Supplymentary" needs correct spelling.
State that the assertion, that retraining is not required, would be mathematically guaranteed given initial training data that were sufficient in the foregoing sense (which is anyway impractical), but in practice is no more than an empirical observation. See my objection below to a similar assertion about retraining.

We thank the reviewer for this detailed clarification. We have revised the manuscript accordingly.

First, we have removed the statement that "QNO learns a resolution-invariant mapping between function spaces" from the manuscript, as we agree this wording over-states the theoretical guarantees available in the current literature. We now describe our findings

more cautiously and explicitly as empirical observations based on the experiments performed in this study.

We have revised the language describing Figures 3-4 to emphasize that the results demonstrate robust empirical performance under spatial irregularity, rather than constituting evidence of resolution-invariance.

We have corrected "Supplymentary" to "Supplementary".

Fig. 2
(a) To my "the legend has 3 colors but the bars have 4" the authors replied accurately but not helpfully "overlapping between categories . . . can give the appearance of more than three . . . colors". Either don't use transparency, or (better) explain in the caption why "overlapping between categories" occurs at all. E.g., if it occurs because the bin edges cross the train/validate/test edges, then choose better bins, and transparency won't matter.

We now use a fixed bin size of one year in the first panel (event time), so that no overlap occurs between categories in this plot. In contrast, overlap naturally remains in the other panels that show the distributions of station count per event, magnitude, and depth. We therefore clarify in the caption that these panels use 60% transparency to make overlapping regions visually interpretable.

The following clarification regarding binning and overlapping has been added to the caption: "A one-year bin size is used to visualize the event time distribution without overlap between categories. Histograms of the number of stations per event, magnitude, and depth are shown using 60% transparency to make overlapping regions visually interpretable."

(b) Similarly, we don't need it to be explained why '"Date" bins are like 0.7 years wide' —It's a poor choice of bin width. Their "balance resolution and visual clarity" goal is not met.

We agree that the previous bin width was not optimal. We have now modified the bin size for the Date histogram to one year, which improves visual clarity and avoids the issues raised.

(c) Their reply to my "the way the . . . datasets were sampled assumes stationarity that is dubious" was "our . . . temporal split was . . . not based on an assumption of

stationarity . . . The underlying data distribution may change over time" that is inconsistent. "Underlying data distribution not changing over time" is the definition of stationarity. Far too many ML researchers eschew traditional jargon: just state plainly that a temporal split of the data follows common practice, but implies an assumption of stationarity (or if they prefer, implies a disregard for the question of stationarity).

We have added the following sentence to the description of the training dataset (Lines 502-503 in the revised manuscript): "following common practice, this temporal split implicitly disregards the question of stationarity"

(d) Thanks for clarifying.

Fig. 3 is improved, but the vectors are too small and clustered together to be interpret visually. Perhaps push this version to Supplementary, and in the main text just show a zoom-in about 100 × 200 km2 or so between San Luis Obispo and Monterey, which will probably be much clearer.

Following the advice, we have generated a zoom-in visualization of the region between San Luis Obispo and Monterey, where event clustering is high and the location-error vectors are noticeably longer than in other regions. This enlarged figure is now provided as Supplementary Fig. 7. In the main text, however, we prefer to retain the full-region California map because it conveys the overall spatial pattern of all events in the test dataset and highlights QNO's performance across diverse tectonic and network geometries. To improve clarity, we have updated Figure 3 by highlighting the two cities and marking the zoom-in region with a bounding box. This allows readers visually connect the main figure with the corresponding enlarged view in the Supplementary.

Fig. 5
(a) Add a caption sentence to explain the curves and lines referred in the plot legend as "PhaseNO" and "manual", since the word "manual" has not appeared since early in the manuscript. It's confusing because "compares the probabilities predicted by QNO and PhaseNO" could refer to the blue curve vs dark-blue line and red curve vs brown line, but none of these are described in the caption. In panels like "BG.SQK..DP" the lines are close to the curve medians, while in panels like "BG.NEG..DP" they aren't. The authors reply implied that their caption is adequate —but it really isn't because "compare" implies 2 things and "probabilities . . . displayed in red" is 1 thing.

We add the following to the Figure caption: "The red and blue curves show the pick probabilities predicted by PhaseNO. The two vertical lines mark the P- and S-wave arrival times from the NCEDC catalog, manually determined by seismologists (dark red for P and dark blue for S)."

The time index corresponding to the maximum probability represents the arrival time picked by PhaseNO, provided that the maximum exceeds the predefined probability threshold. Because the manual picks from the NCEDC catalog are used as training labels for PhaseNO, their agreement serves as a reference for accuracy. While these manual picks are not perfect, they are the best available labels. Therefore, small discrepancies between the manual picks and the peak of the PhaseNO probability curve are acceptable. However, as the SNR decreases, PhaseNO increasingly fails to provide reliable picks, yielding incorrect arrival times and reduced peak probabilities.

(b) Thanks for clarifying.
(c) OK.

197—215 To my request to clarify "enabling the trained QNO to generalize to various seismic network geometries" the authors again baldly assert "QNO . . . learns a mapping between function spaces", without citing Kovachki et al. (whom they do cite in the Supplementary); but the result in op. cit. depends on sufficient sampling, as I explain above. Also note that the op. cit. authors disclaim [their p. 8] "we do not address the challenging open problem of characterizing the error with respect to . . . number of training samples". The present authors correctly reply to me "QNO's prediction is conditioned on these variable sampling layouts" and describe a very good way to address the spatial sampling insufficiency using synthetic data. They do need to qualify their exaggerated claims e.g., "This enables QNO to generalize to unseen station layouts without requiring retraining" (ll. 205–206) should be "In the ideal case of spatial data sampling that is sufficiently globally dense in the sense of Kovachki et al. Definition 4, QNO would generalize to unseen station layouts without requiring retraining", or something similarly acceptably addressing any reader's justified skepticism. See my objection above to a similar assertion about retraining.

We have revised this section in accordance with your suggestion. The relevant paragraph has been updated to include an explicit citation to Kovachki et al. in the main text (in addition to the Supplementary Information), and to clarify the conditions under which generalization to unseen station layouts is theoretically expected.

"As a result, the model encounters a wide range of spatial sampling patterns during training. Each sample can be seen as a different discretization of the spatial domain. In the ideal case of spatial data sampling that is sufficiently globally dense in the sense of (Kovachki et al., 2023, Definition 4), QNO would be expected to generalize to unseen station layouts without requiring retraining. In our numerical results, the evaluation of the trained QNO performs well on unseen station layouts, as shown by its consistent performance across diverse station configurations during the test (Figure 4)."

Fig. 7 OK.

384–394 "Hyperparameter can be optimized" —Deletion is OK.

Secondary comments

1	OK.	Fig. 1	OK.	412, 414	OK.
5	OK.	140–141	OK.	438-442	Authors replied "we apply zero-padding" only to me; rather they should <u>inform</u> the readers.
13	OK.	153	OK.	430	OK.
15	OK.	156	OK.	451–454	Thanks for the lesson.
17–19	OK.	272–273	OK.	470	OK.
21–22	OK.	Table 1	OK.	478-482	OK.
76–78	OK.	340	OK.	487	OK.
106	OK.	Fig. 7	OK.	528	OK.
121	OK.	366	OK.	567	OK.
123–125	OK.	384–392	OK.	567–569	OK.
126	OK.	401	Thanks for the lesson.		
135	OK.	405	OK.		
135–137	OK.	408	OK.		

We have added a description of the zero-padding to the revised manuscript (Lines 437-441).

We thank the reviewer again for the thoughtful review and constructive comments that helped improve the clarity and rigor of the manuscript.

Reviewer #2

Reviewer #2 (Remarks on code availability):

One approaches this manuscript with the hopeful anticipation of a truly "novel deep learning algorithm", as boldly proclaimed by the authors, only to find a familiar landscape thinly disguised by semantic gymnastics. The Quake Neural Operator (QNO), while presented with a flourish, struggles to convincingly differentiate itself from its purported predecessors, particularly the Phase Neural Operator (PhaseNO). Indeed, I still identify this central flaw as "incremental advancement" and a "lack of novelty".

We appreciate the reviewer's time. However, many of the comments rely on broad claims rather than engagement with the technical details presented in either the original submission or the revised manuscript. Several remarks repeat generic statements such as "incremental advancement" or "lack of novelty" without referencing any figures, experiments, or analyses. In multiple instances, the reviewer's descriptions mischaracterize fundamental aspects of QNO that are clearly stated in the manuscript.

We would also like to bring to the attention to the Editor and all reviewers that several statements in Reviewer #2's first-round comments suggested the use of automated text generation (e.g., ChatGPT) without a close reading of the manuscript, for example, remarks such as "scalability and computational cost," which in fact referred to PhaseNO rather than QNO. Even so, we responded carefully and professionally to every point and substantially improved the clarity and positioning of the manuscript in the revised version.

Before addressing the specific points, we note that this misunderstanding from the first round remains uncorrected. In the second-round report, the reviewer does not acknowledge the factual error nor comment on the new experiments, analyses, or added sections, suggesting that QNO and PhaseNO were again conflated and that the revised manuscript was not examined closely. We also note that several second-round statements repeat earlier broad claims without engaging the technical revisions, and the report contains strongly subjective and rhetorical language despite the extensive revisions made specifically to address the reviewer's initial technical concerns.

We respectfully highlight these issues to the Editor and all reviewers because many of the reviewer's current comments rest on similar general statements rather than on technical

evaluation of the manuscript. Nonetheless, for completeness and transparency, we respond to each point below.

The authors declare QNO a "multi-task neural operator" that bypasses "explicit phase picking" to directly build earthquake catalogs. Yet, the very core concept of employing neural operators for seismic event detection and location is, by the authors' own admission, "already established". We are told QNO uses Fourier Neural Operators (FNO) for temporal data and Graph Neural Operators (GNO) for spatial data, much like PhaseNO. The distinctions offered—that PhaseNO is a "per-station phase picker" while QNO performs "end-to-end event detection and location", or that QNO is "multi-task" unlike PhaseNO's "single-task" approach—feel less like groundbreaking innovation and more like a mere reframing of the problem space, a slightly different configuration of existing, well-known deep learning components. As I pointed out, "multi-task learning... is not a novel concept" in machine learning, and its application in seismology has been "previously explored".

Reviewer #2 continued to assert that QNO is merely an incremental extension of PhaseNO, which is definitely incorrect. The PhaseNO remains a phase-picking model (Sun et al. 2023), whereas QNO fundamentally changes the paradigm by eliminating explicit picking and using the entire waveform for simultaneous detection and location. This design makes QNO particularly effective for microseismic monitoring, where many small events lack clear onsets for traditional phase picking.

Also, while QNO employs FNO and GNO layers, these are standard architectural components, much like CNNs or fully connected layers in typical neural networks, and are not claimed as the novelty themselves. Likewise, although we highlight the use of multi-task learning as one of the architectural differences between QNO and PhaseNO, we do not claim that this component alone constitutes the innovation of the method. The innovation lies in how QNO integrates these operator layers to achieve fully end-to-end detection and location without phase picking. For comparison, the EQTransformer model (S. Mostafa et al., 2020) published in *Nature Communications* also used a well-known architecture (Transformer) and was not the first deep-learning phase picker, yet it represented a valuable and widely cited advancement.

While these distinctions were noted in the original submission, the previous revision (Manuscript # NCOMMS-25-07335A) further clarifies and strengthens QNO’s contributions; specifically, we clarified how QNO differs from PhaseNO in the discussion section (Lines 343-354 in Manuscript # NCOMMS-25-07335A and Lines 336-347 in the

current version). In particular, QNO's simpler architecture yields substantially lower computational cost, which we quantified in the previous revision, enabling real-time monitoring (see Results subsection "Computational cost of QNO" and Figure 9).

Unfortunately, Reviewer #2 did not engage with the expanded discussion or the new analyses introduced in the revision, particularly those clarifying the differences between PhaseNO and QNO in terms of the "Problem and rationale" and "Performance." For completeness, we therefore reiterate key points below by restating the relevant parts of our first-round response:

(1) Problem and rationale:

- PhaseNO is a per-station phase picker (P/S arrivals). Pickers can struggle on very low-SNR or emergent onsets where clear phase onsets are unavailable (see Figure 5 and Supplementary Fig. 8-17 on various examples).
- QNO performs end-to-end event detection and location directly from continuous multi-station, waveforms without phase picking, estimating both the presence of an event on each station and its location $(\hat{x}, \hat{y}, \hat{z}, \hat{t})$ in a single unified model.

(2) Learning objectives:

- PhaseNO is a single-task model supervised by phase labels.
- QNO is multi-task, combining classification (probabilities of signal or noise at each input station) and regression of event-level information $\hat{x}, \hat{y}, \hat{z}, \hat{t}$ of all input stations) with a joint loss; no phase labels are required.

(3) Architecture difference:

- PhaseNO uses a U-shaped architecture where input and output share the same temporal sampling.
- QNO uses a simpler architecture with a graph-level feature extractor that aggregates information across stations to produce event-level predictions and supports arbitrary network geometries. QNO yields robust performance at 0 dB SNR where PhaseNO fails (see Figure 5; Supplementary Figs. 8–17).

(4) Performance:

- Across the reported experiments, QNO shows robust detection at low SNR and accurate localization, addressing key failure modes of pick-based pipelines. Comparative results with PhaseNO are provided on the NCEDC test set across Northern California and the Geysers geothermal field; QNO outperforms PhaseNO across the tested settings (see Figure 5; Supplementary Figs. 8–17; Figures 6-8; Supplementary Figs. 21-29).

The claim of a "simpler architecture" for QNO compared to PhaseNO's U-shaped design hardly constitutes a paradigm shift. One might even wonder if "simpler" implies less capable, or merely a different flavor of an already developed methodology.

Again, we do not claim that QNO's lighter architectural design alone constitutes a paradigm shift relative to PhaseNO's U-shaped architecture. It is simply one of the architectural differences between the two models. This simplified design does, however, offer practical advantages. In particular, it provides a significantly lower computational cost, which we quantify in the subsection "Computational cost of QNO" and Figure 9 added during the previous revision, and it enables QNO to operate in real-time seismic monitoring settings.

The paradigm shift lies in how QNO reframes the entire earthquake-catalog–building workflow: instead of a picking-based, sequential or automatic pipeline, QNO performs full-waveform, end-to-end seismic monitoring in a single integrated model without phase picking. This design directly addresses a fundamental challenge in microseismic monitoring, where many small-magnitude events lack clear onsets and exhibit extremely low SNR. As demonstrated in our numerical experiments, QNO successfully detects and locates events even at 0 dB SNR, conditions under which PhaseNO (the state-of-the-art phase picker, outperforming PhaseNet, EQTransformer, and EdgePhase in both detection and accuracy) fail.

Although QNO also uses FNO and GNO layers, this does not diminish its novelty. FNO and GNO, like CNNs or Transformers, can be viewed as standard architectural components; using them does not imply conceptual similarity, just as using CNNs does not make two image-processing models equivalent. We adopt FNO and GNO because they proved effective for PhaseNO's phase-picking task, but the motivation, formulation, and learning objective of QNO are fundamentally different, leading to markedly different behavior and capabilities. **In addition, because PhaseNO and QNO employ the same underlying operator layers (FNO and GNO), the substantial performance difference between the two models further underscores the benefit of reframing seismic monitoring around full-waveform inference without phase picking, rather than attributing gains to architectural components alone.**

While the paper touts QNO's ability to operate "without the need for explicit phase picking", it's worth noting that other deep learning approaches already strive for full automation or integrate picking with subsequent location steps. The novelty, in this regard, appears to be a matter of degree rather than a fundamental breakthrough.

Full automation is an active area of research in seismic monitoring, and we have reviewed recent developments in this direction in the Introduction of the original manuscript (now Lines 53-55 in the current version of the manuscript), where we also highlight the limitations of these approaches. The prior studies referenced by Reviewer #2 fall into the general category of "fully automated" workflows that integrate picking with subsequent location steps. However, these methods still depend on explicit phase picking, which remains a significant limitation for microseismic monitoring. This is because small events constitute the vast majority of microseismicity, yet these events often lack clear onsets at many stations, particularly under low signal-to-noise conditions. As a result, any workflow that relies on pick quality will struggle to construct a complete microseismic catalog.

In contrast, QNO is explicitly designed to remove this bottleneck. QNO does not follow the traditional sequential workflow of picker -> associator -> locator. Instead, it removes both picking and association entirely and replaces the full monitoring pipeline with a single operator trained directly on continuous multi-station waveform data. Unlike integrated pipelines that still rely on phase labels, QNO jointly learns station-level detection and event-level four-dimensional location within one unified operator-learning model.

Even the boasted "robust detection at low SNR" feels like an incremental gain building on the foundation of neural operators already demonstrated to enhance seismic monitoring.

The reviewer's comment appears to overlook the extensive results already included in our original submission, where we benchmarked QNO against PhaseNO on dozens of events in the test dataset. These experiments clearly demonstrate the differences in robustness under low-SNR conditions. For example, as described in the manuscript: "*We introduce real noise waveforms to the raw waveform of this event, adjusting the SNR of the resulting waveform from 20 dB to -8 dB. At an SNR of 20 dB, QNO successfully detects all earthquake signals, outputting a probability close to one for all stations, while PhaseNO accurately identifies both P- and S-wave phases. However, as the SNR decreases, the performance of PhaseNO degrades, failing to detect P phases entirely at an SNR of zero. In contrast, QNO continues to detect the event at most stations even when the SNR reaches zero and consistently determines the event's location.*"

These findings are supported by Fig. 5 and Supplementary Figs. 8–17, which collectively show that QNO fundamentally removes the bottleneck imposed by phase-picking-based workflows. Although PhaseNO improves upon PhaseNet, EQTransformer, and EdgePhase in phase picking, all of these algorithms remain phase pickers. To our knowledge, QNO is

the first method capable of constructing an earthquake catalog directly from continuous waveform data without relying on phase picking, which is a fundamentally different capability rather than an incremental improvement.

While a higher event count in the Geysers geothermal field is reported, the true precision and recall, especially in comparison to actual (and often incomplete) catalogs, remain ambiguous. The "significant advantage" over existing methods remains largely unconvincing, primarily because the underlying mechanisms and conceptual framework are not sufficiently distinct from prior work in the field.

We respectfully disagree with the reviewer's comments that our validation is "ambiguous" or "unconvincing." In both the original submission and the revised manuscript, as well as in our prior response to reviewers, we have provided multiple independent lines of evidence demonstrating the validity of QNO's detections beyond a simple count increase in the Geysers sequence.

Because the NCEDC routine catalog is known to be incomplete, especially for low-magnitude microseismicity, we benchmark QNO against a PhaseNO-based enhanced catalog constructed from the same continuous data using stricter criteria. This removes dependency on the incompleteness of the routine catalog and allows an apples-to-apples comparison.

For clarity, we reiterate the key components of our evaluation, which the reviewer does not specifically address:

1. Precision–recall analysis under two probability thresholds.
We report QNO results at station-level probability thresholds of 0.8 and 0.6. Both QNO catalogs exhibit temporal detection patterns that closely track the independently constructed PhaseNO-based enhanced catalog, across: 8-hour histograms in Figure 6a, and hourly detection rates in Supplementary Fig. 19, including the aftershock-rich window following the 9 June 2024 M4.5 mainshock. Since PhaseNO has been benchmarked against PhaseNet, EQTransformer, and EdgePhase (Sun et al., 2023), agreement with the PhaseNO-based catalog provides strong evidence for the validity of QNO's detections.

2. Cross-catalog location consistency.
We compare event locations across four catalogs: QNO (0.8), QNO (0.6), PhaseNO + GaMMA, and NCEDC routine catalog. The spatial clustering and epicentral distributions

are consistent across all four catalogs (Figure 6b), indicating that the additional QNO detections are not spurious but correspond to genuine seismic events.

3. Waveform-level manual verification.
We manually inspected a large number of QNO detections that were missed by PhaseNO + GaMMA, and provide representative examples in Figures 7–8 and Supplementary Figs. 21–29. These examples demonstrate that:

- picks are present only at a few stations,
- picks are absent or unreliable at many low-SNR stations,
- as a result, the associator eliminates these events due to insufficient picks.

QNO, in contrast, uses full-waveform multi-station information and does not rely on phase picks, allowing it to successfully detect and locate such events.

This manual verification, along with quantitative comparisons and location consistency, provides robust and convergent evidence that the additional QNO detections reflect real seismicity. Moreover, the reviewer suggests that our framework lacks conceptual distinction from prior work; however, these results directly illustrate the fundamental difference:

QNO reconstructs catalogs even when phase picks are unreliable or absent, precisely where pick-based pipelines systematically fail.

In sum, this paper presents a variation on an existing theme, leveraging established neural operator architectures and multi-task learning in a slightly different configuration for seismic event monitoring. While operational refinements and performance improvements are always welcome, the work falls short of delivering the truly "novel" and "substantially different" contribution it professes, echoing the sentiment of "incremental advancement" rather than a significant leap forward.

The reviewer's concluding remark primarily reiterates earlier broad assertions ("incremental advancement," "lack of novelty") without engaging with the specific methodological differences, experiments, and analyses presented in either the original manuscript or the revised version. Importantly, this concluding paragraph does not raise any new technical concerns beyond those already addressed in detail in our responses above.

As noted earlier, QNO differs from prior approaches, including PhaseNO, not merely in "configuration," but in problem formulation, supervision strategy, and algorithmic

capability (direct end-to-end detection and location from continuous multi-station waveforms without picks). These distinctions are supported by extensive empirical evidence, including low-SNR performance, generalization across network configurations, and cross-catalog validation.

We thank the reviewer's time again. However, we respectfully note that the reviewer's summary does not reference any figures, quantitative results, or newly added sections of the revised manuscript, and therefore appears to be a repetition of earlier general statements about the original submission rather than an evaluation of the revised work.

Reviewer #3

Reviewer #3 (Remarks to the Author):

The authors have addressed all my comments and those from the other reviewers in a thorough and convincing manner. I think that the revisions significantly improve the manuscript and clarify key aspects of the methodology and its applications.

We sincerely thank the reviewer for the thoughtful and supportive evaluation. We greatly appreciate the time and care taken to review both the original submission and the revised manuscript in detail. Your recognition that the revisions substantially improved the technical content and clarity is highly encouraging.

Notably, the authors have added a larger number of events to the analysis - this strengthens the validation of QNO's performance and further demonstrates its robustness. The discussion on the model's applicability, including limitations and potential for real-time monitoring, has also been improved and is now more comprehensive.

The manuscript is now much stronger, both in terms of technical content and clarity of presentation.

We are also grateful for your acknowledgement that the expanded analyses, increased number of events, and strengthened discussion helped clarify the methodology and its applicability. Your constructive guidance throughout the process has been invaluable.

I just have one minor point: In the supplementary materials, some figures have non-optimal formatting (oversized fonts, label overlaps). While this does not detract from the main results, improving figure quality would enhance readability. Probably the authors did not have much time to invest before the deadline for the revision.

Regarding the minor comment on figure formatting in the Supplementary Information, we have carefully polished all figures during the revision. For a few figures, particularly those showing network geometry, the station density varies greatly across the region, and some label overlap is unavoidable. In these cases, the goal is to illustrate overall geometry rather than emphasize individual station names. We minimized font sizes while ensuring compliance with journal requirements (i.e., no smaller than body-text size). In the last two Supplementary figures, slight overlap (e.g., for “depth”) remains due to these constraints, though the overlapping elements are semi-transparent and do not affect readability.

I recommend acceptance.

We thank the reviewer again for the helpful feedback and positive recommendation.

Referee report for “Microseismic Monitoring with Quake Neural Operator” (NCOMMS-25-07335)

Aimé Fournier, MIT

March 22, 2025

This work introduces a new algorithm to build earthquake catalogs from seismic data without phase picking. Creating such catalogs is crucial to the study of natural earthquakes, but also and especially for monitoring of seismicity induced by geothermal energy development, mining, carbon capture and storage etc. The manuscript is carefully written and discusses its results thoroughly. Following are my Primary and Secondary comments.

Primary comments

31–41 I assume these are useful citations. You should also summarize to what extent the DL approaches are quantitatively more accurate than the human ones, if that is true, aside from cost and time issues.

80 “neural operator architectures”, will we read a summary of what that means? Most readers won’t know. How is a NO different from other NNs?

84–121 None of this belongs in “**Results**”, because it’s all “**Methods**” exposition.

94–95 “QNO can be evaluated on varying grids without requiring model retraining” is a very strong claim, and seems unlikely on its face. Justify it.

Fig. 2
- (a) The legend has 3 colors but the bars have 4.
- (b) The “Date” bins are like 0.7 years wide.
- (c) “Both training and test datasets cover a wide range of depth and magnitude” appears to be true, but not impressively so. The way the training, validation, and test datasets were sampled assumes stationarity that is dubious. Instead, re-sample by repeatedly generating uniformly random indexes into the data, with replacement for each dataset, but empty intersection between datasets. Lines 361–363 don’t justify this simple splitting.
- (d) Why are some depths negative? If “depth” is referenced to sea level, then say so. Also address at Line 409.

Fig. 3 is hard to interpret because one can’t tell how many markers overlap each other. Instead, make 3 plots side-by-side with station markers and land-sea & state boundaries, without topographic coloring, and overlay contours of the KDE of the QNO and NCEDC results in the 1st 2 plots, and of the difference of KDEs in the 3rd plot.

Fig. 5
- (a) The caption refers to “probabilities predicted by QNO and PhaseNO” but the plot legend shows only “PhaseNO” and “manual”.

(b) Do "Z, N, E" denote depth, north and east components, or what? Say in Caption.

(c) Why is time error not monotonic vs SNR? Discuss.

Fig. 7 Same comment as for Fig. 3, above.

273–274 "this hyperparameter can be optimized based on the inter-event times from past seismic history" —say more about this. Do you mean "optimize" in the loose sense that one can obtain better results by some empirical hyperparameter assignment, or in the tight sense that a certain hyperparameter value minimizes some loss function?

292–293 "enabling the trained QNO to generalize to various seismic network geometries" is ambiguous. Do you mean that it can be trained and operate on any arbitrary geometry, or that it can be trained on one arbitrary geometry and operate on another arbitrary geometry? The latter seems much less likely so, worth emphasizing and explaining if it's the case. And then, how different must the geometries be before results degrade?

Secondary comments

1 "Microseismic Monitoring with Quake Neural Operator" → "Microseismic Monitoring with the Quake Neural Operator" to signify what you're introducing.

5 "seismic phase picking" i.e., manually, or how? State in Abstract.

12 "Our model" → "We show that our model", if you show that.

15 "over 3,000", give the number.

15–16 "significantly surpassing", state in the Abstract how we know the events are real.

19 "industry applications" yes, but in the Abstract name a few that you mean.

64–66 "as these approaches rely on the waveforms of detected events, they are not directly applicable to continuous data streams" is hard to understand. Including waveforms doesn't imply discontinuities. Do you mean waveforms should be omitted simply to keep data streams smaller?

93 "location" → "location and time".

106 "multiple stations with irregular geometries" → "multiple stations at irregularly distributed locations", unless you mean each of the stations is weirdly shaped.

108–110 "Each input tensor contains six channels: three corresponding to the three-component waveforms and three corresponding to the station locations (x_i, y_i, z_i)" should be clearer. The locations share a single index i, so it seems none of x, y, z can vary independently from the other 2. Part of Fig. 1 suggests the input has the form $((f_{ji}(t) = f_j(x_i, y_i, z_i, t))_{j=1}^{3})_{i=1}^{n}$ that is a tensor of type 2 and shape $3 \times n$ for each t. It's likely that you mean that location information is also input, that would imply a tensor $(f_{1i}(t), f_{2i}(t), f_{3i}(t), x_i, y_i, z_i))_{i=1}^{n}$ of type 2 and shape $6 \times n$ for each t, that is ambiguous in Fig. 1. Clarify.

111, 380 "15 seconds" at what sampling rate $1/\triangle t$? Is it 100 Hz (l. 178)?

120 "noise" → "estimated noise", at least the 1st time you introduce this. One never knows the true noise.

120–121 "mean across the station axis, consisting of four channels: $\hat{x}$, $\hat{y}$, $\hat{z}$, and $\hat{t}$" → "mean of four channels $\hat{x}_i$, $\hat{y}_i$, $\hat{z}_i$, and $\hat{t}_i$ across the station axis" (if correct), to avoid the trivial misinterpretation that the earthquake locations would be means of station locations; but it's questionable if you mean that each station generates its own $(\hat{x}_i, \hat{y}_i, \hat{z}_i, \hat{t}_i)$. If you don't mean that, then it's questionable if $(\hat{x}, \hat{y}, \hat{z}, \hat{t})$ is really a mean.

Fig. 1 Define FNO and GNO in the caption.

128 "in their best status" means what?

134 "multi-layer perception", did you mean "multi-layer perceptron"?

137 "much accurate" → "much more accurate" (presumably).

219 "similar high" → "similarly high".

Table 1 Explain parenthetic numbers in caption.

257 "multi-stations" → "multiple stations".

Fig. 8 "PhaseNO did not find picks on many stations that contain earthquake signals classified by QNO", make this a specific quantified statement.

267 "deep-learning" → "the deep-learning".

283 Is "multi-task" the same as "multi-objective"?

288 "time series" → "time coordinate".

291 "operator" → "operators".

295 For clarity, write "$\mathbf{p} = (\mathbf{p}_i)_{i=1}^n \in \mathbb{R}^{n\times 2}$" here, and ...

297 ... "$\mathbf{p}_i \in \mathbb{R}^2$" here.

306–307 "using Fourier transforms", mention how you handle that the t-series is not periodic, so a jump from $f(t_m)$ to $f(t_1)$ would always imply some spurious fat-tail spectrum.

320 "respectively" → "respectively for $k = 3$", since it's a new paragraph.

325 "Gaussian Error Linear Unit", explain briefly.

337 "we suggest" is very odd. You should just say what you did.

334 "and β" —There really is no reason to have 2 weights, correct?

353 "is the true values" → "contains the true values".

386 "straight" → "straightforward".

388–389 "we typically use" —Say precisely what you did for this work.

424 "Adaptive Moment Estimation method" needs a citation and a brief summary here.

425 "$10e^{-4}$" → "10^{-4}" (unless you really do mean $10\exp(-4)$) (and similarly if such a typo recurs).

Referee report for revised “Microseismic Monitoring with Quake Neural Operator” (NCOMMS-25-07335A)

Aimé Fournier, MIT

September 28, 2025

This work introduces a new algorithm to build earthquake catalogs from seismic data without phase picking. Creating such catalogs is crucial to the study of natural earthquakes, but also and especially for monitoring of seismicity induced by geothermal energy development, mining, carbon capture and storage etc. The authors’ replies to my last report are mostly acceptable, with exceptions noted below. It is very important not to assert a result as if it were established rigorously mathematically vs merely supported empirically.

Primary comments

Six citations are to arXiv manuscripts, or lack publication details. Is that acceptable to the journal? Can they be replaced by citations to refereed work?

45–47 My “extent the DL approaches are quantitatively more accurate” was addressed. Not all readers know if “0.05 s” is that small so, report MAE divided by the picked times.

93 To my “neural operator architectures” authors replied with “we have added a review section … at the beginning of the Supplementary Information”. So cite that.

97–137 Authors disagree with my ‘none of this belongs in “**Results**”’. While I agree with their “design of QNO … constitutes a key outcome”, reading 40 ll. about methodology before results really saps exposition momentum. Push all but the 1st sentence to **Methods**.

106–108 Authors reply to my ‘“QNO … on varying grids without … model retraining” … seems unlikely’ with helpful empirical results, but also by baldly asserting “QNO learns a resolution-invariant mapping between function spaces”, which is just another kind of very strong i.e., dubious claim.

(a) They refer to the Supplementary, but there is only one mention of “resolution” (just in the temporal sense) and no mentions of “invariant” in the Supplementary.

(b) The Supplementary (but not the main text) cites Kovachki et al., who define *discretization invariant* in terms of a *discrete refinement* $(D_n)_{n=1}^{\infty}$ of the problem domain D, requiring that $\forall\epsilon > 0\ \exists L \in \mathbb{N}$ such that $D \subseteq$ the union of all radius-ϵ balls centered on all $x \in D_L$. This requirement is key in the *op. cit.* Appendix E proof of Theorem 8, but is not fulfilled by the spatial part of the authors’ data domain.

(c) Furthermore, the *op. cit.* Definition 4 defines *invariance* as the risk vanishing only in the infinite sample-count L limit. For $L < \infty$ and non-fulfilling D_n as in the authors’ case, resolution invariance is not guaranteed.

(d) Figures 3, 4 compare QNO to NCEDC and SeismicGNN estimates, and show that QNO can handle spatially irregularly sampled data, which are nice results, but do not really illustrate resolution-invariance.

(e) "Supplymentary" needs correct spelling.

State that the assertion, that retraining is not required, would be mathematically guaranteed given initial training data that were sufficient in the foregoing sense (which is anyway impractical), but in practice is no more than an empirical observation. See my objection below to a similar assertion about retraining.

Fig. 2 (a) To my "the legend has 3 colors but the bars have 4" the authors replied accurately but not helpfully "overlapping between categories ... can give the appearance of more than three ... colors". Either don't use transparency, or (better) explain in the caption why "overlapping between categories" occurs at all. E.g., if it occurs because the bin edges cross the train/validate/test edges, then choose better bins, and transparency won't matter.

(b) Similarly, we don't need it to be explained why '"Date" bins are like 0.7 years wide' —It's a poor choice of bin width. Their "balance resolution and visual clarity" goal is not met.

(c) Their reply to my "the way the ... datasets were sampled assumes stationarity that is dubious" was "our ... temporal split was ... not based on an assumption of stationarity ... The underlying data distribution may change over time" that is inconsistent. "Underlying data distribution not changing over time" is the definition of stationarity. Far too many ML researchers eschew traditional jargon: just state plainly that a temporal split of the data follows common practice, but implies an assumption of stationarity (or if they prefer, implies a disregard for the question of stationarity).

(d) Thanks for clarifying.

Fig. 3 is improved, but the vectors are too small and clustered together to be interpret visually. Perhaps push this version to Supplementary, and in the main text just show a zoom-in about 100×200 km^2 or so between San Luis Obispo and Monterey, which will probably be much clearer.

Fig. 5 (a) Add a caption sentence to explain the curves and lines referred in the plot legend as "PhaseNO" and "manual", since the word "manual" has not appeared since early in the manuscript. It's confusing because "compares the probabilities predicted by QNO and PhaseNO" could refer to the blue curve vs dark-blue line and red curve vs brown line, but none of these are described in the caption. In panels like "BG.SQK..DP" the lines are close to the curve medians, while in panels like "BG.NEG..DP" they aren't. The authors reply implied that their caption is adequate —but it really isn't because "compare" implies 2 things and "probabilities ... displayed in red" is 1 thing.

(b) Thanks for clarifying.

(c) OK.

197—215 To my request to clarify "enabling the trained QNO to generalize to various seismic network geometries" the authors again baldly assert "QNO ... learns a mapping between function spaces", without citing Kovachki et al. (whom they do cite in the Supplementary); but the result in *op. cit.* depends on sufficient sampling, as I explain above. Also note that the

op. cit. authors disclaim [their p. 8] "we do not address the challenging open problem of characterizing the error with respect to … number of training samples". The present authors correctly reply to me "QNO's prediction is conditioned on these variable sampling layouts" and describe a very good way to address the spatial sampling insufficiency using synthetic data. They do need to qualify their exaggerated claims e.g.,

"This enables QNO to generalize to unseen station layouts without requiring retraining" (ll. 205–206)	should be	"In the ideal case of spatial data sampling that is sufficiently globally dense in the sense of Kovachki et al. Definition 4, QNO would generalize to unseen station layouts without requiring retraining",

or something similarly acceptably addressing any reader's justified skepticism. See my objection above to a similar assertion about retraining.

Fig. 7 OK.

384–394 "Hyperparameter can be optimized" —Deletion is OK.

Secondary comments

1 OK.

5 OK.

13 OK.

15 OK.

17–19 OK.

21–22 OK.

76–78 OK.

106 OK.

121 OK.

123–125 OK.

126 OK.

135 OK.

135–137 OK.

Fig. 1 OK.

140–141 OK.

153 OK.

156 OK.

272–273 OK.

Table 1 OK.

340 OK.

Fig. 7 OK.

366 OK.

384–392 OK.

401 Thanks for the lesson.

405 OK.

408 OK.

412, 414 OK.

438-442 Authors replied "we apply zero-padding" only to me; rather they should inform the readers.

430 OK.

451–454 Thanks for the lesson.

470 OK.

478-482 OK.

487 OK.

528 OK.

567 OK.

567–569 OK.

Referee report for revised "Microseismic Monitoring with Quake Neural Operator" (NCOMMS-25-07335A)

Aimé Fournier, MIT

September 28, 2025

This work introduces a new algorithm to build earthquake catalogs from seismic data without phase picking. Creating such catalogs is crucial to the study of natural earthquakes, but also and especially for monitoring of seismicity induced by geothermal energy development, mining, carbon capture and storage etc. The authors' replies to my last report are mostly acceptable, with exceptions noted below. It is very important not to assert a result as if it were established rigorously mathematically vs merely supported empirically.

Primary comments

Six citations are to arXiv manuscripts, or lack publication details. Is that acceptable to the journal? Can they be replaced by citations to refereed work?

45–47 My "extent the DL approaches are quantitatively more accurate" was addressed. Not all readers know if "0.05 s" is that small so, report MAE divided by the picked times.

93 To my "neural operator architectures" authors replied with "we have added a review section ... at the beginning of the Supplementary Information". So cite that.

97–137 Authors disagree with my 'none of this belongs in "**Results**"'. While I agree with their "design of QNO ... constitutes a key outcome", reading 40 ll. about methodology before results really saps exposition momentum. Push all but the 1st sentence to **Methods**.

106–108 Authors reply to my '"QNO ... on varying grids without ... model retraining" ... seems unlikely' with helpful empirical results, but also by baldly asserting "QNO learns a resolution-invariant mapping between function spaces", which is just another kind of very strong i.e., dubious claim.

(a) They refer to the Supplementary, but there is only one mention of "resolution" (just in the temporal sense) and no mentions of "invariant" in the Supplementary.

(b) The Supplementary (but not the main text) cites Kovachki et al., who define *discretization invariant* in terms of a *discrete refinement* $(D_n)_{n=1}^{\infty}$ of the problem domain D, requiring that $\forall \epsilon > 0\ \exists L \in \mathbb{N}$ such that $D \subseteq$ the union of all radius-ϵ balls centered on all $x \in D_L$. This requirement is key in the *op. cit.* Appendix E proof of Theorem 8, but is not fulfilled by the spatial part of the authors' data domain.

(c) Furthermore, the *op. cit.* Definition 4 defines *invariance* as the risk vanishing only in the infinite sample-count L limit. For $L < \infty$ and non-fulfilling D_n as in the authors' case, resolution invariance is not guaranteed.

(d) Figures 3, 4 compare QNO to NCEDC and SeismicGNN estimates, and show that QNO can handle spatially irregularly sampled data, which are nice results, but do not really illustrate resolution-invariance.

(e) "Supplymentary" needs correct spelling.

State that the assertion, that retraining is not required, would be mathematically guaranteed given initial training data that were sufficient in the foregoing sense (which is anyway impractical), but in practice is no more than an empirical observation. See my objection below to a similar assertion about retraining.

Fig. 2 (a) To my "the legend has 3 colors but the bars have 4" the authors replied accurately but not helpfully "overlapping between categories ... can give the appearance of more than three ... colors". Either don't use transparency, or (better) explain in the caption why "overlapping between categories" occurs at all. E.g., if it occurs because the bin edges cross the train/validate/test edges, then choose better bins, and transparency won't matter.

(b) Similarly, we don't need it to be explained why '"Date" bins are like 0.7 years wide' —It's a poor choice of bin width. Their "balance resolution and visual clarity" goal is not met.

(c) Their reply to my "the way the ... datasets were sampled assumes stationarity that is dubious" was "our ... temporal split was ... not based on an assumption of stationarity ... The underlying data distribution may change over time" that is inconsistent. "Underlying data distribution not changing over time" is the definition of stationarity. Far too many ML researchers eschew traditional jargon: just state plainly that a temporal split of the data follows common practice, but implies an assumption of stationarity (or if they prefer, implies a disregard for the question of stationarity).

(d) Thanks for clarifying.

Fig. 3 is improved, but the vectors are too small and clustered together to be interpret visually. Perhaps push this version to Supplementary, and in the main text just show a zoom-in about 100×200 km^2 or so between San Luis Obispo and Monterey, which will probably be much clearer.

Fig. 5 (a) Add a caption sentence to explain the curves and lines referred in the plot legend as "PhaseNO" and "manual", since the word "manual" has not appeared since early in the manuscript. It's confusing because "compares the probabilities predicted by QNO and PhaseNO" could refer to the blue curve vs dark-blue line and red curve vs brown line, but none of these are described in the caption. In panels like "BG.SQK..DP" the lines are close to the curve medians, while in panels like "BG.NEG..DP" they aren't. The authors reply implied that their caption is adequate —but it really isn't because "compare" implies 2 things and "probabilities ... displayed in red" is 1 thing.

(b) Thanks for clarifying.

(c) OK.

197—215 To my request to clarify "enabling the trained QNO to generalize to various seismic network geometries" the authors again baldly assert "QNO ... learns a mapping between function spaces", without citing Kovachki et al. (whom they do cite in the Supplementary); but the result in *op. cit.* depends on sufficient sampling, as I explain above. Also note that the

op. cit. authors disclaim [their p. 8] “we do not address the challenging open problem of characterizing the error with respect to … number of training samples”. The present authors correctly reply to me “QNO’s prediction is conditioned on these variable sampling layouts” and describe a very good way to address the spatial sampling insufficiency using synthetic data. They do need to qualify their exaggerated claims e.g.,

“This enables QNO to generalize to unseen station layouts without requiring retraining” (ll. 205–206)	should be	“In the ideal case of spatial data sampling that is sufficiently globally dense in the sense of Kovachki et al. Definition 4, QNO would generalize to unseen station layouts without requiring retraining”,

or something similarly acceptably addressing any reader’s justified skepticism. See my objection above to a similar assertion about retraining.

Fig. 7 OK.

384–394 “Hyperparameter can be optimized” —Deletion is OK.

Secondary comments

1 OK.

5 OK.

13 OK.

15 OK.

17–19 OK.

21–22 OK.

76–78 OK.

106 OK.

121 OK.

123–125 OK.

126 OK.

135 OK.

135–137 OK.

Fig. 1 OK.

140–141 OK.

153 OK.

156 OK.

272–273 OK.

Table 1 OK.

340 OK.

Fig. 7 OK.

366 OK.

384–392 OK.

401 Thanks for the lesson.

405 OK.

408 OK.

412, 414 OK.

438-442 Authors replied “we apply zero-padding” only to me; rather they should inform the readers.

430 OK.

451–454 Thanks for the lesson.

470 OK.

478-482 OK.

487 OK.

528 OK.

567 OK.

567–569 OK.

Referee report for revised “Microseismic Monitoring with Quake Neural Operator” (NCOMMS-25-07335B)

Aimé Fournier, MIT

March 6, 2026

This work introduces a new algorithm to build earthquake catalogs from seismic data without phase picking. Creating such catalogs is crucial to the study of natural earthquakes, but also and especially for monitoring of seismicity induced by geothermal energy development, mining, carbon capture and storage etc. I recommend to accept the current revision for publication.

Comments

The authors’ replies to my last report are acceptable. I also note several revised passages that convincingly establish the novelty and impact of their work.

- Ll 168–170 “as the SNR decreases, the performance of PhaseNO degrades, failing to detect P phases entirely at an SNR of zero. In contrast, QNO continues to detect the event at most stations even when the SNR reaches zero and consistently determines the event’s location.”
- Ll 274–279 “QNO successfully identified 17 earthquake signals out of 36 input stations for both events . . . PhaseNO only made picks on a few input waveforms . . . low-quality events . . . filtered out during association . . . Even if the association threshold is lowered, the uncertainties in phase picking caused by strong noise would still propagate to the subsequent location step.”
- Ll. 337–342 “PhaseNO is formulated as a per-station picking task that outputs P/S arrival times and relies on labeled picks for supervision; its outputs are station-level and are typically passed to a downstream associator/locator. In contrast, QNO is an end-to-end, event-level model that operates directly on continuous, multi-station wave-forms without picks, jointly learning detection (classification) and location . . . under a single multi-task objective.”
